# Integrating chemical, genetic, and feasibility assessments for anti-tubercular target validation

**DOI:** 10.1038/s44321-026-00415-7

**Published:** 2026-04-07

**Authors:** Dirk Schnappinger, Steven J Berthel, Helena I M Boshoff, Inna V Krieger, Paridhi Sukheja, Saswati Panda, Siddhant Rath, Kelsey Briggs, Xuelin Bian, Sari Rasheed, Laura A T Cleghorn, Sandeep Ghorpade, Dirk A Lamprecht, Nader Fotouhi, Rolf Müller, Carl Nathan, Tanya Parish, Kyu Rhee, Peter Warner, Case W McNamara, Jeremy M Rock, James C Sacchettini, Valerie Mizrahi

**Affiliations:** 1 Department of Microbiology and Immunology, Weill Cornell Medicine, New York, NY 10065 USA; 2 Panorama Global, Seattle, WA USA; 3 Tuberculosis Research Section, Laboratory of Clinical Immunology and Microbiology, National Institute of Allergy and Infectious Diseases (NIAID), National Institutes of Health (NIH), Bethesda, MD 20892 USA; 4 Department of Biochemistry and Biophysics, Texas A&M University, College Station, TX USA; 5 Calibr-Skaggs Institute for Innovative Medicines, A Division of Scripps Research, La Jolla, CA USA; 6 Gates Medical Research Institute, Cambridge, MA USA; 7 Helmholtz Institute for Pharmaceutical Research Saarland (HIPS), Helmholtz Centre for Infection Research (HZI), PharmaScienceHub (PSH), Saarland University Campus, 66123 Saarbrücken, Germany; 8 Drug Discovery Unit, Division of Biological Chemistry and Drug Discovery, School of Life Sciences, University of Dundee, Dundee, DD1 5EH UK; 9 Holistic Drug Discovery and Development (H3D) Centre, Department of Chemistry, University of Cape Town, Rondebosch, 7701 South Africa; 10 TB Alliance: Global Alliance for Tuberculosis Drug Development, New York, NY USA; 11 Department of Tropical Disease Biology, Liverpool School of Tropical Medicine, Pembroke Place, Liverpool, L3 5QA UK; 12 Division of Infectious Diseases, Weill Department of Medicine, Weill Cornell Medicine, New York, NY 10021 USA; 13 Gates Foundation, Seattle, WA USA; 14 Laboratory of Host-Pathogen Biology, The Rockefeller University, New York, NY 10065 USA; 15 Molecular Mycobacteriology Research Unit, Institute of Infectious Disease and Molecular Medicine & Department of Pathology, University of Cape Town, Cape Town, South Africa

**Keywords:** Antibacterial Drug Development, Target Assessment, Tuberculosis, Database, Microbiology, Virology & Host Pathogen Interaction

## Abstract

Despite the approval of two first-in-class anti-tuberculars over the past two decades, the global burden of tuberculosis (TB) remains unacceptably high, in part due to the emergence and spread of drug-resistant strains of *Mycobacterium tuberculosis* (*Mtb*). This review summarizes advances and ongoing challenges in anti-TB drug discovery, focusing on identifying and validating novel targets. Highlighted is a framework developed by the TB Drug Accelerator (TBDA) consortium for target validation in *Mtb*. Two computational platforms, DAIKON and PARSNIP, allow the systematic evaluation of targets across multiple dimensions, including chemical validation, genetic essentiality, vulnerability, and the feasibility to identify drug-like molecules for a target of interest. Case studies of Pks13 and NadE illustrate how these parameters guide target prioritization and risk assessment. By integrating these metrics, the framework enables dynamic, transparent target ranking, supporting development of both pan-TB and treatment-shortening regimens. This paradigm is adaptable to other bacterial pathogens and is designed to improve evidence-based decision-making in antibacterial drug discovery.

## Abstract (web summary)

In this review, Dirk Schnappinger, Valerie Mizrahi and colleagues discuss recent advances in Tuberculosis drug discovery, highlighting a systematic target-validation framework that integrates genetic, chemical, and computational evidence to prioritize therapeutic targets in *Mycobacterium tuberculosis*.

## Graphical abstract

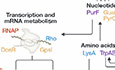

## Introduction

Over the past two decades, four first-in-class antibiotics have reached the clinic: Bedaquiline (BDQ) and Delamanid (DLM), both conditionally approved for the treatment of drug-resistant (DR) tuberculosis (TB), and Lefamulin and Gepotidacin, approved for community-acquired pneumonia and urinary tract infection, respectively (Covvey and Guarascio, 2022; Keam, 2025; Zumla et al, 2014). BDQ and DLM (as well as pretomanid, another nitroimidazole in clinical use (Showalter, 2020)) were developed specifically to treat TB, leading to a shorter, less toxic, all oral regimen for DR-TB (Conradie et al, 2020). Despite these advances, TB claimed ~1.5 million lives in 2024. Of these ~27% were lost due to infection with multidrug-resistant (MDR) or rifampicin-resistant (RR) strains of *Mtb* (W.H.O., 2025). At a minimum, decreasing the global burden of TB will require continued innovation to (i) create additional antibiotics leading to even shorter, more accessible drug regimens; (ii) develop accurate, rapid and affordable point-of-care tests for diagnosing TB infection and disease and for detecting drug resistance; and (iii) develop an effective vaccine that provides protection across a range of age groups beyond that afforded by the BCG vaccine.

Development of BDQ and DLM began with phenotypic screening (Andries et al, 2005; Matsumoto et al, 2006), a strategy that uses inhibition of bacterial growth to identify candidate molecules for drug development. In fact, almost all antibiotics currently approved for clinical use were developed by optimizing whole-cell-active compounds identified in phenotypic screens. There is every reason to continue to pursue this well-validated approach to antibiotic discovery. Among the innovations that promise to further enhance the productivity of this approach are media that more closely mimic the conditions bacterial pathogens encounter in vivo (Pethe et al, 2010), screens against intracellular *Mtb* (Christophe et al, 2009; VanderVen et al, 2015), an expansion of the accessible chemical space, and genetic approaches to steer hits towards molecules with desired mechanisms of action (MOA) (Abrahams et al, 2012; Bond et al, 2025; Johnson et al, 2019).

However, the mechanisms by which hits from phenotypic screens inhibit bacterial growth vary widely in both complexity and clinical utility. Some molecules, such as BDQ, act by inhibiting a single, essential protein or protein complex (in this case, ATP synthase (Guo et al, 2021)). Others, like pyrazinamide (PZA), exert their effects through mechanisms that may not depend on engagement of a single target protein (Lamont et al, 2020). Importantly, many compounds identified from phenotypic screens lack specificity and are broadly toxic. Identifying a hit molecule’s MOA, ideally including its molecular target, can thus help focus resources on the hits with the greatest therapeutic potential.

Launching antibiotic development by conducting high-throughput screens against isolated targets has been challenging, primarily because in vitro screens do not select for physicochemical properties that enable active compounds to accumulate within the bacterial cell (Payne et al, 2007; Silver, 2011). Nevertheless, there are now several candidates stemming from target-based screens in (pre)clinical development (https://www.newtbdrugs.org/). The first key step in target-based drug discovery is to select a target that is susceptible to inhibition by a drug-like molecule and whose inactivation achieves the desired therapeutic effect.

Here, we discuss the target assessment strategy developed by the TB Drug Accelerator (TBDA), a collaborative network dedicated to discovering and developing new drugs and regimens for TB (Aldridge et al, 2021). Central to this strategy is DAIKON (Rath et al, 2023), an integrated project-tracking database featuring a target-centric interface and automated scoring capabilities. To broaden access beyond the TBDA, a companion tool—PARSNIP (Protein Assessment and Ranking System for Novel Input from Public users)—has recently been developed. PARSNIP offers the same assessment modules as DAIKON but is designed for public use and excludes proprietary data (For simplicity, we primarily refer to PARSNIP, but all statements made about PARSNIP also apply to DAIKON. The PARSNIP source code is available in the GitHub repository (https://github.com/sidxz/parsnip), which also provides the link to the current live version of the application). A central goal of both tools is to complement existing strategies developed for other pathogens (Godinez-Macias et al, 2025) by providing a systematic, consistent, and transparent framework for target evaluation and prioritization.

## Goals of TB drug development

The primary objectives of TB drug development are to: (i) develop universal or ‘pan-TB’ regimens that are entirely or largely unencumbered by existing drug resistance, and (ii) substantially shorten the duration of treatment to less than two months without compromising efficacy or safety. While distinct, these goals are interlinked. First, DR-TB remains a stubbornly persistent problem globally (Farhat et al, 2024). Recent estimates indicate that in 2024, 390,000 people developed and 150,000 succumbed to RR or MDR-TB (W.H.O., 2025). Following a period of slow decline, the global incidence of RR/MDR-TB has remained stable since 2020. However, the introduction of new and repurposed drugs to treat MDR-TB (Nyang’wa et al, 2022) has imposed new selective pressures on *Mtb*, resulting in ongoing evolution of the genotypic drug resistance landscape. This necessitated updating the definition of extensively DR-TB (XDR-TB), which is now defined as MDR-TB that is also resistant to a late-generation fluoroquinolone (moxifloxacin or levofloxacin) and at least one of the newer drugs, BDQ or linezolid (W.H.O., 2022). The first goal of TB drug development thus faces a ‘moving goalpost’, which must be borne in mind when developing new TB regimens. The importance of identifying compounds that are active against drug-sensitive and DR strains of *Mtb* representative of the diversity of strains in circulation globally places a premium on mechanistic novelty in regimen development. This in turn influences which targets are selected, prioritized and potentially combined, and why.

Second, the goal of treatment-shortening is the subject of ongoing research and debate. A recent perspective on treatment-shortening strategies offered short- and long-term options, categorized according to the ease and complexity of implementation and their associated trade-offs (Dartois et al, 2025). A central pillar of these strategies is recognition of the importance of differentiating “easy-to-treat” (ETT) from “hard-to-treat” (HTT) TB cases (Dartois et al, 2025). In an idealized scenario that builds on the “stratified medicine” approach proposed by Rada Savic and colleagues (Imperial et al, 2018), drug regimen, doses and treatment duration would be optimized at the level of the individual patient according to composite signatures of host- and pathogen-derived biomarkers that (i) can distinguish ETT and HTT at baseline and guide the composition of the drug regimen; (ii) are indicative of treatment response allowing determination of when it is safe to stop treatment; and (iii) allow identification of asymptomatic (subclinical) TB cases. Importantly, these strategies recognize the different phenotypes adopted by *Mtb* during the transmission-infection-disease cycle. Phenotypic heterogeneity further compounds these challenges as subpopulations of genetically identical bacilli adapt differentially to the dynamic environments encountered during transmission, infection and disease. This includes the varied forms and degrees of organ pathology that can evolve and co-exist along the spectrum of disease states from asymptomatic to advanced TB (Chung et al, 2022; Warner et al, 2025; Zaidi et al, 2023). These issues must be considered when evaluating both new and existing targets.

Within the TBDA, key features of the target candidate profile (TCP) for compounds with treatment-shortening potential are an ability to (i) disrupt cellular processes essential for *Mtb* survival in a non- or slowly replicating state in which it displays phenotypic drug tolerance (Helaine et al, 2024), and (ii) reach and penetrate the bacilli located in complex host microenvironments (Prideaux et al, 2015), most notably, caseum, the cellular debris found at the necrotic core of a tuberculous lesion (Sarathy and Dartois, 2020). Advancement of compounds demonstrating this profile has been facilitated by the development of a caseum surrogate model (Sarathy et al, 2023) in which *Mtb* is strictly nonreplicating (Sarathy, 2024). Such tools provide an important adjunct to the powerful genetic approaches that are being used to identify the most vulnerable targets in *Mtb* under host-relevant conditions (Bosch et al, 2024). Here, “vulnerability” refers not to the ease of achieving inhibition of the target with a given chemical compound, but the fitness cost to the organism of incomplete inhibition, as typically achieved with drugs whose binding is reversible.

## Combining multiple features to assess targets for TB drug development

**Figure 1:**
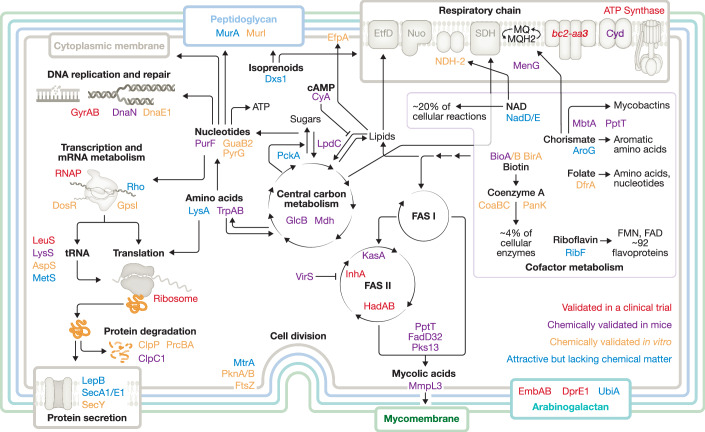
Pathway and target map. This graphic depicts pathways (bold) containing *Mtb* targets for which inhibitors or activators exist that (i) inhibit growth of *Mtb* and for which (ii) multiple lines of evidence support that growth inhibition is mediated via engagement of the target listed. Targets are considered clinically validated when compounds have shown activity in an EBA (early bactericidal activity) study. Detailed information on each target in this map is provided in Supplementary Table 1.

Ultimately, validation of a bacterial drug target requires evidence that its engagement by a drug candidate contributes to clinical efficacy, as evidenced, at a minimum, in a meaningful reduction in bacterial load. In *Mtb*, RNA polymerase (Wehrli, 1983), the ribosome (Kumar et al, 2021), InhA (Sankar et al, 2024), EmbAB (Telenti et al, 1997), DNA gyrase (Sarathy et al, 2019a), LeuS (Diacon et al, 2024), ATP synthase (Sarathy et al, 2019b), cytochrome *bc*_1_-*aa*_3_ oxidase (Janssen et al, 2025), HadAB (MRC, 1985) and DprE1 (Heinrich et al, 2025) currently meet this standard for clinical validation based on drugs with clinical efficacy that act on these targets (Fig. 1, Table EV1). Drug candidates that act on other *Mtb* proteins are at higher risk of failure during clinical development owing to a lack of data that mitigate this risk. Furthermore, when developing new compounds that target clinically validated targets, it is essential to carefully consider the MOA of existing drugs. For instance, fluoroquinolones exert their effect by converting DNA gyrase into a DNA-cleaving enzyme (Aldred et al, 2014), a DNA-damaging mechanism that is particularly important for their efficacy against caseum-adapted *Mtb* (Ashwath et al, 2024). DNA gyrase inhibitors that lack such DNA-damaging activity may therefore not achieve the same clinical outcomes as fluoroquinolones.

The following sections outline strategies developed by the TBDA to mitigate the risk of failure by considering multiple target features including (i) evaluating efficacy by assessing the impact of target modulation in preclinical studies, (ii) addressing the feasibility of discovering potent small-molecule modulators, and (iii) minimizing toxicity through careful target selection.

### Target features determining impact

#### Chemical target validation

Chemical validation is the demonstration that a drug-like molecule can engage a target in live *Mtb* and inhibit the pathogen’s growth or kill it. Such validation is highly desirable for establishing a target’s suitability for TB drug development. Consequently, whenever selective small-molecule inhibitors (or activators) are available, their activity profile in vitro, ex vivo and in vivo then drives that target’s impact assessment. To determine which *Mtb* subpopulations present in patients might be affected via modulation of a target’s function, candidate molecules are ideally analyzed under a range of in vitro conditions. These should capture impact on both replicating *Mtb* (grown with in vivo relevant carbon sources) and nonreplicating (NR) *Mtb*. NR models include exposure to low pH (Early et al, 2019), hypoxia (Rao et al, 2008; Wayne and Hayes, 1996), multiple concurrent stresses (Gold et al, 2021), model aerosol fluid (Mishra et al, 2025) and adaptation to rabbit caseum (Sarathy and Dartois, 2020) or caseum surrogate (Sarathy, 2024; Sarathy et al, 2023). The target assessment algorithm in PARSNIP assigns higher scores to targets if their engagement affects *Mtb* under multiple conditions, with particular emphasis on activity against caseum-adapted *Mtb*, given its critical role in achieving cure for HTT patients (Dartois et al, 2025). Naturally, targets for which engagement has been shown to affect *Mtb* during infection, e.g., in a mouse model of TB, also score higher than those lacking in vivo proof of concept.

**Figure 2:**
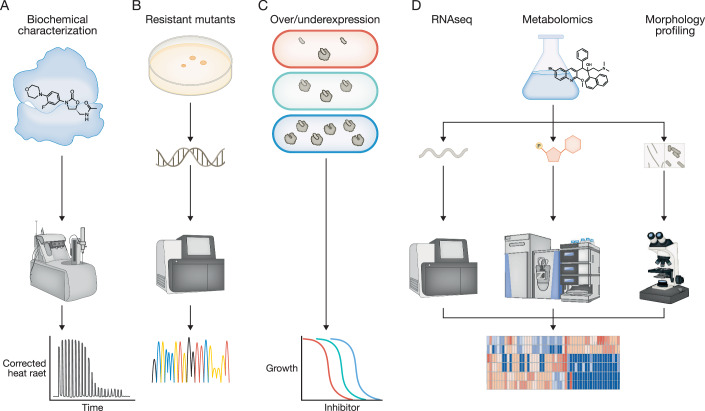
Experimental strategies to support ascertainment of on-target activity of a drug candidate. On-target activity can be provided by demonstrating that (**A**) the candidate molecule interacts with the purified target (e.g., via isothermal titration calorimetry), (**B**) mutations in the target encoding can cause resistance, (**C**) variation in expression of the target cause the expected change in susceptibility to growth inhibition, and (**D**) exposure to the candidate molecule causes expected changes in the transcriptome, metabolome or cell morphology.

While chemical validation is essential, it is equally important to confirm that a candidate molecule’s effect on *Mtb* is truly due to engagement of the cognate target. Evidence supporting on-target activity can include (i) demonstration of binding or inhibition of the purified target; (ii) isolation of preservation-of- function resistance-conferring mutations in the gene encoding the target or participating in its synthesis; (iii) changes in levels of target expression that correspond to predicted shifts in compound potency; and (iv) proof that exposure to the molecule perturbs the expected pathway, as shown, for example, by RNAseq, metabolomics or morphologic profiling (Fig. 2). In PARSNIP, a combination of in vitro and in vivo evidence is required to classify a compound as acting on target.

#### Genetic target validation

When engagement of a target by a candidate molecule with demonstrated on-target activity has been shown to attenuate *Mtb* in an animal model of TB then PARSNIP does not require genetic evidence to evaluate a target. However, these types of data are only available for a small number of targets (Fig. 1, Table EV1). For all other targets, PARSNIP therefore takes genetic data into account, acknowledging that genetic inactivation does not always recapitulate pharmacological inhibition. For instance, genetic silencing reduces target protein abundance, whereas a small molecule may selectively inhibit only one functional domain of the protein, leaving scaffolding or non-catalytic roles intact. This distinction is exemplified by the *Mtb* ClpP2 protease subunit, where chaperone-binding activity is essential but proteolytic activity is dispensable (d’Andrea et al, 2022). In addition, genetic knockdown resembles the effect of a noncompetitive inhibitor, while small molecules can exert diverse biochemical effects ranging from antagonism to agonism. Examples for the latter include V-59 (Wilburn et al, 2022) and the clinical candidate, GSK286, which are thought to primarily block cholesterol catabolism in *Mtb* by putatively acting as agonists of the adenylyl cyclase, CyA (Rv1625c) (Brown et al, 2023).

Nearly all approved antibiotics act on the products of genes that are essential in vitro. This may reflect an ascertainment bias, since almost all antibiotics were first identified by their effects on the pathogen in vitro. Nonetheless, the historical record makes the products of in vitro-essential genes logical candidates for drug discovery. While most essential genes are conserved across strains, there are isolate-specific exceptions (Bosch et al, 2021; Carey et al, 2018). Furthermore, essentiality is context dependent: some genes dispensable in culture become critical during infection, reflecting the unique nutrient milieu and immune pressures of the host. For example, over 200 genes that are dispensable in standard laboratory media have been shown by transposon mutagenesis to be required by *Mtb* for growth in mice (Sassetti and Rubin, 2003; Smith et al, 2022). Certain *Mtb* genes are essential in vitro but dispensable in mice because *Mtb* can salvage needed metabolites from the host. For example, NADH precursors render the *nadABC* genes non-essential during infection despite being essential for growth in standard liquid media (Boshoff et al, 2008; Vilcheze et al, 2010). Differences in available carbon sources during infection can also render some in vitro inhibitors ineffective in vivo (Pethe et al, 2010). Therefore, confirming a target’s essentiality under infection-relevant conditions and across diverse clinical isolates is critical (Bateson et al, 2022). Genetic ablation of target function in established infections has validated the in vivo essentiality of proteins involved in respiration, carbon metabolism, transcription, and biosynthetic pathways (Beites et al, 2019; Botella et al, 2017; Evans et al, 2016; Hasenoehrl et al, 2019; Kim et al, 2013; Marrero et al, 2010; Puckett et al, 2014; Woong Park et al, 2011). Genetic evidence that inactivation attenuates *Mtb* during infection earns the gene a higher score in PARSNIP than for genes that lack such evidence.

Antibiotics rarely achieve complete target inhibition. Consequently, binary genetic definitions of essentiality fail to identify targets that inflict major fitness costs even with partial inhibition. As noted earlier, this relationship is captured by the concept of *gene vulnerability*, which describes how reductions in gene expression translate to fitness loss, treating essentiality as a quantitative spectrum. Here, the term “vulnerable” is used interchangeably to characterize essential genes and their encoded targets. Vulnerable targets are promising because partial inhibition can impair growth or survival, whereas invulnerable targets require near-total inhibition, raising the bar for drug discovery. Early vulnerability studies leveraged inducible promoters or targeted proteolysis (Evans et al, 2016; Wei et al, 2011) but were not easily scalable. The advent of CRISPR interference (CRISPRi) enabled systematic, tunable knockdown of nearly every gene in the *Mtb* genome, revealing that essential genes encode targets that span a range of vulnerabilities, from encoding highly sensitive “weak links” such as RNAP and InhA—the targets of rifampicin and isoniazid, respectively—to remarkably invulnerable enzymes. The latter include CoaA/PanK and peptide deformylase, whose invulnerability may help explain why multiple drug discovery campaigns against them have failed (Bosch et al, 2021). Importantly, both essentiality and vulnerability can vary with strain background and growth conditions. This underscores the need to extend vulnerability profiling beyond laboratory reference strains and standard in vitro assay conditions to infection-relevant environments to increase the likelihood that prioritized targets will be vulnerable during the pathophysiologic stresses that *Mtb* encounters in vivo.

Vulnerability is a powerful metric for prioritizing drug targets, complementing essentiality and druggability. Thus, the proteins encoded by vulnerable genes score higher in PARSNIP than those encoded by invulnerable ones. However, even invulnerable targets can be exploited if irreversible inhibition and other favorable pharmacological properties can be achieved. For example, BioA, an enzyme required for the synthesis of biotin and essential for growth in biotin-limited conditions, is highly invulnerable to partial inhibition (Woong Park et al, 2011) but can be targeted with amiclenomycin, an irreversible covalent inhibitor, to block growth of *Mtb* (Shi et al, 2011).

In summary, the TBDA leverages genetic data to assess targets that currently lack chemical matter capable of target engagement during infection or for which no suitable chemical probes exist. The consortium prioritizes candidates for resource-intensive biochemical screening by (i) systematically evaluating gene essentiality and vulnerability across a range of infection-relevant conditions and (ii) investigating the impact of genetically inactivating specific *Mtb* genes during infection. Several of the targets that are currently prioritized by the TBDA consortium are illustrated in Fig. 1 and described in more detail in Table EV1.

### Feasibility features

Isocitrate lyase (ICL) is an example of a biologically well-validated target that nevertheless failed in drug development. Although the causes of failure are often complex, the small, highly polar active site of ICL likely prevented screens from yielding enzyme inhibitors with whole-cell activity (Bhusal et al, 2017; Wellington and Hung, 2018). The purpose of a feasibility assessment is therefore to estimate the likelihood that a target will yield small molecules that can be advanced to a developable series without major safety liabilities. In PARSNIP, this assessment is structured into four independently scored modules: (1) suitability for biochemical screening, (2) structure-based drug discovery potential, (3) progressibility, and (4) safety. Scoring is based primarily on currently available data and assets, such as validated biochemical assays and high-quality structural information, rather than prospects for future development. Targets are prioritized if there are existing whole-cell-active modulators that demonstrate on-target activity or have entered clinical studies. Compounds are prioritized once they have met in vitro, in vivo, and clinical safety criteria.

*Module 1* evaluates the feasibility of performing a biochemical screen by assessing the availability of purified protein, validated assays, and results of prior screening campaigns. Information about orthologs, especially from related mycobacteria, is considered when *Mtb*-specific data are lacking. *Module 2* examines the resources available for virtual screening or rational design, focusing on the quality and availability of *Mtb* protein structures, ideally with co-crystallized ligands, or binding pocket analyses of apo structures using predictive tools (Borrel et al, 2015; Le Guilloux et al, 2009). As in *Module 1*, orthologous data supplement *Mtb*-specific gaps. The goal is to determine if structure-based approaches can complement or substitute for empirical screening. *Module 3* considers progressibility—prospects for the advancement of compounds targeting the protein or its close homologs. This includes whether the target belongs to a well-established “druggable” class, whether assays exist to confirm on-target activity in *Mtb*, and how far previous hits have progressed. When *Mtb* data are sparse, assessment again draws from information on orthologs and related systems. This module thus provides a performance history for the target or target class. *Module 4*, safety, addresses potential off-target liabilities—particularly the presence and role of human orthologs and the consequences of their inhibition. Whenever possible, target safety is judged by success in clearing hurdles in vitro, in animal models and in clinical barriers.

To summarize overall feasibility, a composite score is calculated by integrating results from all four feasibility modules for each target. However, the scoring system is flexible and can be adapted for specific use cases. For example, if the primary interest is in “screen-for-hits” drug discovery, the structure-based module may be omitted.

### Integrating impact and feasibility

Impact and feasibility assessments result in numerical scores that can be combined in a straightforward manner, with adjustable weighting factors for individual features (chemical validation, genetic validation, feasibility). The scoring methodology also accounts for logical hierarchies among questions. For example, if a high-priority criterion is met—such as the identification of a potent small-molecule inhibitor—certain follow-up questions become redundant and are not considered when calculating the final score. Similarly, if a chemical inhibitor of the target exists, genetic validation information becomes less relevant. If an *Mtb* target structure is available, ortholog structure questions are not scored. Although these streamlining rules may not always be universally applicable, they are embedded in the calculation process for efficiency and clarity.

**Figure 3:**
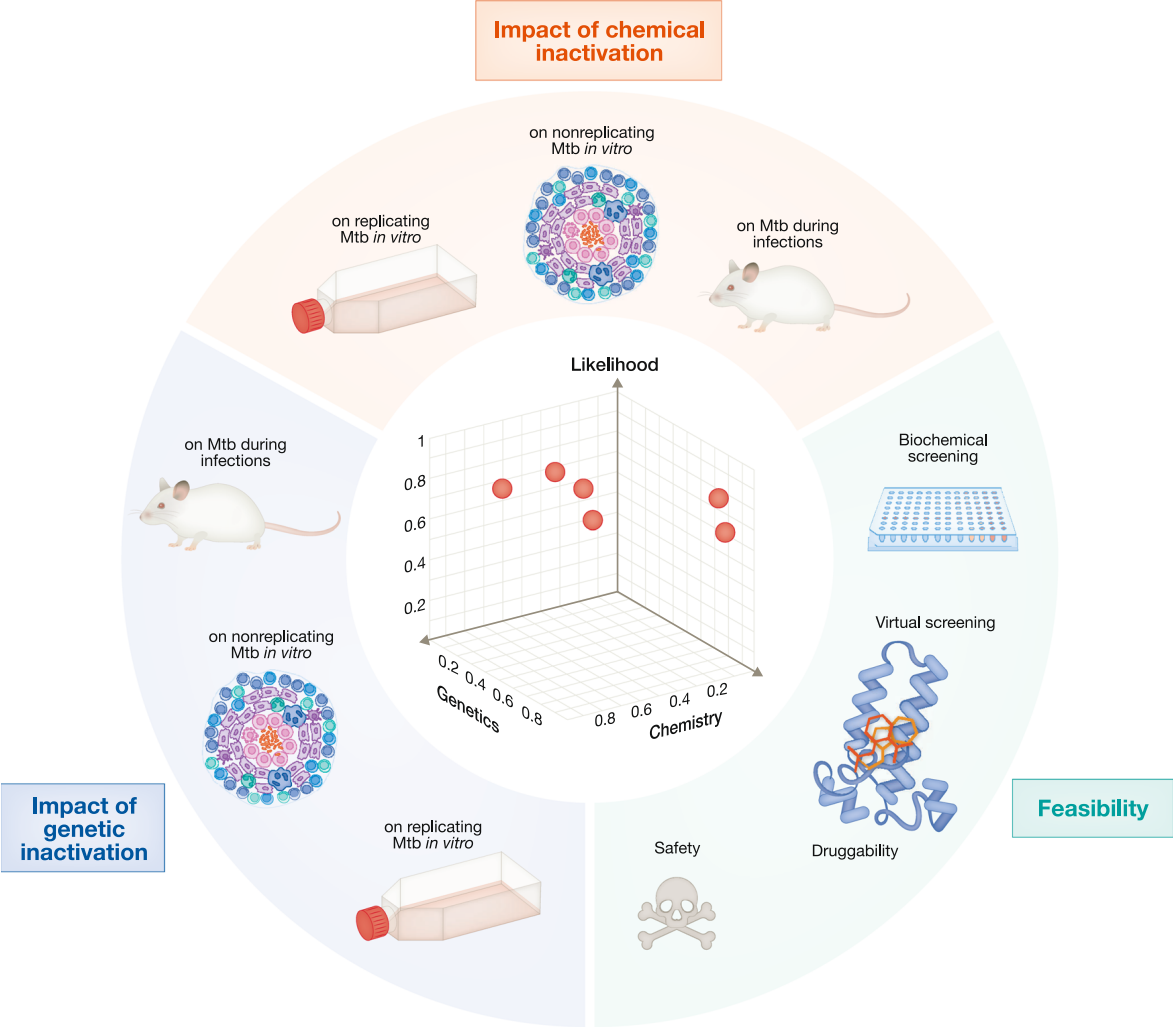
Integrating impact and feasibility assessments. This figure illustrates integration of impact assessment derived from (i) chemical and (ii) genetic inactivation of a target with an assessment of (iii) the likelihood that drug-like small molecules can be identified for this target. Scores derived from each assessment are displayed in a 3D space in which the x, y, and z axes represent the impact derived from chemical inactivation, genetic inactivation and feasibility. The 3D space intended to provide an illustration without providing information about specific targets. An annotated version of this graph providing information for specific targets is available on the PARSNIP website.

Once scores for impact and feasibility are normalized (on a 0 to 1 scale), targets can be visualized in two or three dimensions. Figure 3 illustrates a three-dimensional visualization in which individual axes denote the impact of chemical or genetic target inactivation and feasibility. Presenting data from chemical and genetic inactivation on separate axes enhances the resolution for targets where only genetic inactivation information is available. When both types of data are combined on a single axis, targets with only genetic data are visually compressed into a limited area due to the greater weighting of chemical inactivation scores. Scores are dynamic; as new information becomes available, a target’s position in the map can shift accordingly.

The following sections describe case studies of targets situated in distinct regions of the target assessment map, both of which are currently pursued by the TBDA consortium. Pks13 was initially selected based on early indications of druggability, while NadE is being pursued on the grounds of genetic inactivation data that suggest a substantial impact on *Mtb* viability.

## Case study 1: Pks13, a novel druggable target

Polyketide synthase 13 (Pks13) is a type I polyketide synthase that catalyzes the final condensation step in mycolic acid biosynthesis, joining meromycolyl and α-chain precursors to produce the long-chain mycolic acids. These are the most abundant lipids in the outermost layer of the *Mtb* cell envelope and they confer structural integrity and impermeability. As a result, Pks13 is essential for *Mtb* viability (DeJesus et al, 2017; Gavalda et al, 2014) and helps sustain resistance to host immune defenses and antibiotics (Xia et al, 2023). Inhibition of Pks13 also leads to the toxic accumulation of fatty acid precursors within the cell, creating multifaceted stresses that further enhances bactericidal activity (Kim et al, 2023). Reflecting these functions, Pks13 has emerged as a highly vulnerable target in CRISPRi genome-wide depletion studies. The frequency of spontaneous resistance to several Pks13 inhibitors is substantially lower than for FDA-approved cell wall inhibitors like isoniazid or ethambutol, suggesting a higher barrier to the development of resistance for at least some Pks13 inhibitors (Krieger et al, 2024; Krieger et al, 2025).

Pks13 is found only in mycobacteria and corynebacteria, with no close homologs in humans or most commensal bacteria (Xia et al, 2023). This specificity ensures excellent species selectivity for specific inhibitors of Pks13, minimizing risks of host toxicity and disruption of the microbiome. Structural analyses of Pks13’s individual domains and of the full-length protein (Kim et al, 2023) have revealed multiple druggable binding sites, providing a strong foundation for rational drug development. Most recently, CMX410 was identified as a covalent Sulfur(VI) fluoride exchange (SuFEx) inhibitor that irreversibly inactivates the catalytic serine (Ser801) within the acyltransferase domain. CMX410 displays low-nanomolar potency against drug-sensitive, MDR, and XDR *Mtb* isolates and retains efficacy in persistence models and mouse infection studies (Krieger et al, 2025). Earlier studies laid important groundwork: resistance to antitubercular thiophenes was first linked to mutations in *pks13*, suggesting that Pks13 could be a druggable target (Ioerger et al, 2013; Wilson et al, 2013); the activity of benzofurans such as TAM16 mapped to the thioesterase (TE) domain (Wilson et al, 2022); coumestans provided a chemically distinct series optimized to mitigate hERG liability (Lun et al, 2021; Zhang et al, 2022); and DNA-encoded libraries expanded ligand diversity for the TE-domain (Krieger et al, 2024).

Despite this promise, several hurdles remain for clinical development. The compound’s access to Pks13 may be limited by the enzyme’s intracellular location and its hydrophobic substrate binding environment. The compound may struggle to reach Mtb in caseous granulomas, where drug diffusion is poor. Resistance may arise through mutations in *pks13* or the activation of compensatory pathways. Optimizing pharmacokinetics and safety, along with identifying synergistic companion agents, will be essential to unlock the full clinical potential and treatment-shortening promise of Pks13 inhibitors. Nevertheless, Pks13 stands out as a compelling new drug target for TB owing to its essential role in mycolic acid biosynthesis, vulnerability, lack of human homologs, and tractability to inhibition by small molecules.

## Case study 2: NadE, a genetically prioritized target without chemical inhibitors

NAD(H) is a critical cofactor not only for metabolic redox reactions but also as an allosteric regulator, a substrate for NAD^+^-dependent DNA ligases, sirtuin-like enzymes, and ADP-ribosyltransferases, enzymes responsible for post-translational modification of several key proteins (Burton et al, 2009; Raffaelli et al, 2004). In *Mtb*, the Sir2-like protein Rv1151c requires NAD+ for deacylating lysines in proteins such as MbtA (Vergnolle et al, 2016), isocitrate lyase (Bi et al, 2017), histone-like proteins (Anand et al, 2017), acyl-CoA synthases (Hayden et al, 2013; Nambi et al, 2013; Xu et al, 2011; Yang et al, 2015), and the transcriptional regulator DosR (Bi et al, 2018; Yang et al, 2018), thereby impacting mycobactin biosynthesis, epigenetic regulation, central metabolic flux, and the dormancy response. Moreover, NAD(H) is the precursor of NADP(H), which provides the ultimate reductant for many anti-oxidant pathways in *Mtb*.

Mycobacteria synthesize NAD^+^ de novo from aspartate or recycle it from host-derived nicotinamide and nicotinic acid (Boshoff et al, 2008). The final steps of NAD^+^ biosynthesis, catalyzed by NaMN adenylyltransferase (NadD) and NAD synthetase (NadE), are shared across both the recycling and de novo pathways. Unable to import NAD^+^ from their environment, mycobacteria depend on NadD and NadE to generate NAD^+^. Depletion of either enzyme therefore causes a dramatic loss of viability in vitro (Kim et al, 2013; Rodionova et al, 2014; Sharma et al, 2023). NadE depletion, in particular, is rapidly bactericidal for *Mtb* in both actively growing and nonreplicating states, and during both acute and chronic phases of mouse infection, suggesting treatment-shortening potential (Kim et al, 2013).

In the absence of chemical inhibitors, target validation of NadE and NadD relies on predicting the effects of pharmacological inhibition. It is therefore significant that partial genetic depletion of NadE is sufficient to arrest *Mtb* growth. CRISPRi-mediated titration of NadD and NadE revealed that these enzymes are much more vulnerable to depletion in *Mtb* than in *E. coli* or *B. subtilis* (Bosch et al, 2021). Metabolomic profiling demonstrated that a modest threefold reduction in NAD(H) pools induces adaptive metabolic remodeling and glyoxylate shunt activation, leading to bacteriostasis. During infection, this adaptation synergizes with the antimicrobial effects of host-derived itaconic acid (Priya et al, 2025), which affects the glyoxylate shunt via isocitrate lyase inhibition. In addition, depletion of NAD+ compromises DNA repair necessary to combat oxidative DNA damage inflicted by host cell defenses. Reduction of NAD+ pools also compromises Rv1151c-dependent activation of metabolic programs known to be essential in vivo, including mycobactin biosynthesis and transcriptional control of the DosR/S/T “dormancy” regulon. More pronounced NadE inhibition catastrophically reduces bacterial respiratory capacity, eradicating both replicating and nonreplicating bacilli. NAD(H) pool levels are tightly correlated with viability, and complete growth inhibition coincides with a tenfold drop in NAD(H) levels (Sharma et al, 2023).

Humans possess redundant NAD^+^ biosynthesis pathways (Tempel et al, 2007), reducing the risk of toxicity for inhibitors of NadE (and NadD) from *Mtb*. Nevertheless, inhibitor development will require careful assessment of human enzyme inhibition as well as off-target effects likely driven by ligands that engage the ATP or glutamine-binding pockets. The human NAD synthetase, like *Mtb* NadE, is an octameric glutamine-dependent NAD synthetase, but the orthologs only share 23% amino acid identity. The reaction coupling mechanisms between domains are different in the human and *Mtb* enzymes. Differences in nicotinic acid adenine dinucleotide binding residues as well as in a loop in the synthetase domain also offer the prospect of designing selective inhibitors of the Mtb enzyme (Chuenchor et al, 2020; LaRonde-LeBlanc et al, 2009).

In summary, targeting NadE has all the hallmarks of a treatment shortening strategy in that tubercle bacilli in every host microenvironment and every metabolic state tested are vulnerable to its inhibition, and such inhibitors are expected to further synergize with host defenses. Unfortunately, despite the target being well enabled for biochemical screens and having a pocket that is considered druggable, inhibitors reported to date have demonstrated only modest potency for inhibiting the enzyme and for inhibiting growth of *Mtb* (Boshoff et al, 2008; Wang et al, 2017). More potent compounds are required as tools for chemical validation of NadE during different stages of the disease and in different pathological environments encountered in vivo.

## Outlook

Antibacterial drug discovery is challenged by the dynamic complexity of bacterial pathogenesis, the rapid emergence of resistance, the permeability barrier of bacterial cell envelopes and the ability of bacterial pathogens to persist in diverse physiological states. Adding to these challenges in the case of *Mtb* is this organism’s remarkable ability to metabolize xenobiotics (Singh et al, 2023) and to expel many drugs through efflux (Remm et al, 2022). Over the 13 years of its existence, the TBDA consortium has worked on developing and refining strategies to identify, validate, and translate promising targets into clinically effective therapies. Recent years have seen substantial progress toward this goal, driven by omics analyses, high throughput screening technologies, and efficient use of in vivo models. The target assessment framework described here applies computational tools to integrate and visualize a target’s feasibility and impact features. Such an approach offers broad applicability to other infectious diseases and stands to gain from expanding genome-wide druggability assessments (Halder et al, 2025) for bacterial pathogens. Moving from bench to bedside will require a deeper understanding of the biological mechanisms governing treatment duration and a systematic evaluation of novel targets in the context of existing drug combinations. This suggests the need for an additional axis for prioritization that scores how well an inhibitor of a given target is likely to work in combination with inhibitors of other targets to shorten treatment. However, it will be challenging to know how early in drug development to give weight to this given the uncertainty in how much the answer will depend on specific properties of the compound series independent of the biological target or pathway, and what the other drugs in the regimen will be.

### Pending issues

- Which target and regimen properties determine treatment shortening in humans, and how early in discovery can these properties be predicted?
- How should feasibility metrics (screenability, structure availability, safety, progressibility) be weighed against biological impact when prioritizing targets?
- How can platforms like DAIKON and PARSNIP be generalized and adapted for other pathogens to accelerate antibacterial drug discovery more broadly?

## Supplementary information

Table EV1

## Glossary

**Caseum** and **caseous granulomas**: Caseum is cheese-like, dead tissue found at the center of some TB lesions in the lung. These “caseous granulomas” can shelter bacteria in hard-to-reach pockets where several drugs penetrate poorly. Adaptation to NR persistence in caseum also changes the metabolic state of *Mtb*, which profoundly reduces its susceptibility to most antibiotics.
**CRISPR interference (CRISPRi)**: A gene-silencing technique derived from CRISPR technology that allows the expression of individual genes to be “dialed down” to study how important they are for growth and survival.
**Druggability**: Describes how likely it is that a target, such as a protein, can be modulated effectively and safely by a drug-like small-molecule compound. Druggability assessments generally include evaluating whether a target protein has a well-defined pocket where a compound can bind with sufficient affinity and selectivity.
**hERG liability**: In drug discovery, “hERG liability” means that a compound blocks the KCNH2/Kv11.1 channel, which is crucial for repolarizing the cardiac action potential and thus for normal QT interval and rhythm. Inhibition of this channel can cause sudden death and hERG assays are widely used as safety pharmacology screens in drug discovery.
**Phenotypic screening**: A drug discovery approach that searches for compounds that, in the case of antibacterial drug discovery, can kill or stop the growth of bacteria, without knowing in advance exactly which cellular function they disrupt.
**SuFEx inhibitor**: SuFEx stands for sulfur(VI) fluoride exchange, a class of robust yet selectively activatable click reactions where an S–F bond on a sulfur center is replaced by an incoming nucleophile such as Ser, Thr, Tyr, Lys, His or Cys on a protein. “SuFEx inhibitor” refers to inhibitors discovered or optimized using SuFEx click chemistry.
**Target candidate profile (TCP)**: A predefined list of properties that a drug candidate should possess (for example, activity against nonreplicating bacteria or good penetration into lesions) to achieve a specific clinical goal, such as shortening treatment.
**Target-based screening**: A drug discovery approach that tests compounds directly against a specific bacterial protein known to be required by the bacterial pathogen to cause disease.
**Tuberculosis (TB)**: An infectious disease caused by *Mtb* that most often affects the lungs and when present in clinically active form, is often deadly if not treated properly. *Mtb* spreads through the air when a person with TB of the lungs coughs, sneezes, sings, shouts or even breathes normally. Depending on the drug susceptibility profile of the Mtb strain present in a patient, TB is classified as drug-susceptible (DS-TB); drug-resistant (DR-TB, caused by bacteria that no longer respond to one or more of the standard TB medicines, making treatment longer, more complex, more toxic, more expensive, and often less effective); multidrug-resistant (MDR-TB, which is resistant to at least isoniazid and rifampicin, two of the most powerful first-line TB drugs); rifampicin-resistant (RR-TB, in which the bacteria are resistant to rifampicin, one of the key TB drugs); or extensively drug-resistant (XDR-TB, a severe form of DR-TB where the bacteria are resistant to rifampicin and isoniazid, to certain fluoroquinolones, and to at least one of the newer TB drugs such as bedaquiline or linezolid).

## References

[CR1] Abrahams GL, Kumar A, Savvi S, Hung AW, Wen S, Abell C, Barry 3rdCE, Sherman DR, Boshoff HI, Mizrahi V (2012) Pathway-selective sensitization of Mycobacterium tuberculosis for target-based whole-cell screening. Chem Biol 19:844–854 22840772 10.1016/j.chembiol.2012.05.020 PMC3421836

[CR2] Aldred KJ, Kerns RJ, Osheroff N (2014) Mechanism of quinolone action and resistance. Biochemistry 53:1565–1574 24576155 10.1021/bi5000564 PMC3985860

[CR3] Aldridge BB, Barros-Aguirre D, Barry 3rdCE, Bates RH, Berthel SJ, Boshoff HI, Chibale K, Chu XJ, Cooper CB, Dartois V et al (2021) The Tuberculosis Drug Accelerator at year 10: what have we learned? Nat Med 27:1333–1337 34226736 10.1038/s41591-021-01442-2 PMC10478072

[CR4] Anand C, Garg R, Ghosh S, Nagaraja V (2017) A Sir2 family protein Rv1151c deacetylates HU to alter its DNA binding mode in Mycobacterium tuberculosis. Biochem Biophys Res Commun 493:1204–1209 28935371 10.1016/j.bbrc.2017.09.087

[CR5] Andries K, Verhasselt P, Guillemont J, Gohlmann HW, Neefs JM, Winkler H, Van Gestel J, Timmerman P, Zhu M, Lee E et al (2005) A diarylquinoline drug active on the ATP synthase of Mycobacterium tuberculosis. Science 307:223–227 15591164 10.1126/science.1106753

[CR6] Ashwath P, Osiecki P, Weiner D, Via LE, Sarathy JP (2024) Role of DNA double-strand break formation in gyrase inhibitor-mediated killing of nonreplicating persistent Mycobacterium tuberculosis in caseum. ACS Infect Dis 10:3631–3639 39315541 10.1021/acsinfecdis.4c00499 PMC11474946

[CR7] Bateson A, Ortiz Canseco J, McHugh TD, Witney AA, Feuerriegel S, Merker M, Kohl TA, Utpatel C, Niemann S, Andres S et al (2022) Ancient and recent differences in the intrinsic susceptibility of Mycobacterium tuberculosis complex to pretomanid. J Antimicrob Chemother 77:1685–1693 35260883 10.1093/jac/dkac070 PMC9155602

[CR8] Beites T, O’Brien K, Tiwari D, Engelhart CA, Walters S, Andrews J, Yang HJ, Sutphen ML, Weiner DM, Dayao EK et al (2019) Plasticity of the Mycobacterium tuberculosis respiratory chain and its impact on tuberculosis drug development. Nat Commun 10:4970 31672993 10.1038/s41467-019-12956-2 PMC6823465

[CR9] Bhusal RP, Bashiri G, Kwai BXC, Sperry J, Leung IKH (2017) Targeting isocitrate lyase for the treatment of latent tuberculosis. Drug Discov Today 22:1008–1016 28458043 10.1016/j.drudis.2017.04.012

[CR10] Bi J, Gou Z, Zhou F, Chen Y, Gan J, Liu J, Wang H, Zhang X (2018) Acetylation of lysine 182 inhibits the ability of Mycobacterium tuberculosis DosR to bind DNA and regulate gene expression during hypoxia. Emerg Microbes Infect 7:108 29899473 10.1038/s41426-018-0112-3 PMC5999986

[CR11] Bi J, Wang Y, Yu H, Qian X, Wang H, Liu J, Zhang X (2017) Modulation of central carbon metabolism by acetylation of isocitrate lyase in Mycobacterium tuberculosis. Sci Rep 7:44826 28322251 10.1038/srep44826 PMC5359664

[CR12] Bond AN, Orzechowski M, Zhang S, Ben-Zion I, Lemmer A, Garry N, Lee K, Chen M, Delano K, Gath E et al (2025) Reference-based chemical-genetic interaction profiling to elucidate small molecule mechanism of action in Mycobacterium tuberculosis. Nat Commun 16:9673 41184262 10.1038/s41467-025-64662-x PMC12583738

[CR13] Borrel A, Regad L, Xhaard H, Petitjean M, Camproux AC (2015) PockDrug: a model for predicting pocket druggability that overcomes pocket estimation uncertainties. J Chem Inf Model 55:882–895 25835082 10.1021/ci5006004

[CR14] Bosch B, DeJesus MA, Poulton NC, Zhang W, Engelhart CA, Zaveri A, Lavalette S, Ruecker N, Trujillo C, Wallach JB et al (2021) Genome-wide gene expression tuning reveals diverse vulnerabilities of M. tuberculosis. Cell 184:4579–4592.e4524 34297925 10.1016/j.cell.2021.06.033 PMC8382161

[CR15] Bosch B, DeJesus MA, Schnappinger D, Rock JM (2024) Weak links: advancing target-based drug discovery by identifying the most vulnerable targets. Ann N Y Acad Sci 1535:10–19 38595325 10.1111/nyas.15139

[CR16] Boshoff HI, Xu X, Tahlan K, Dowd CS, Pethe K, Camacho LR, Park TH, Yun CS, Schnappinger D, Ehrt S et al (2008) Biosynthesis and recycling of nicotinamide cofactors in Mycobacterium tuberculosis. An essential role for NAD in nonreplicating bacilli. J Biol Chem 283:19329–19341 18490451 10.1074/jbc.M800694200 PMC2443648

[CR17] Botella L, Vaubourgeix J, Livny J, Schnappinger D (2017) Depleting Mycobacterium tuberculosis of the transcription termination factor Rho causes pervasive transcription and rapid death. Nat Commun 8:14731 28348398 10.1038/ncomms14731 PMC5379054

[CR18] Brown KL, Wilburn KM, Montague CR, Grigg JC, Sanz O, Perez-Herran E, Barros D, Ballell L, VanderVen BC, Eltis LD (2023) Cyclic AMP-mediated inhibition of cholesterol catabolism in Mycobacterium tuberculosis by the novel drug candidate GSK2556286. Antimicrob Agents Chemother 67:e0129422 36602336 10.1128/aac.01294-22 PMC9872607

[CR19] Burton RL, Chen S, Xu XL, Grant GA (2009) Role of the anion-binding site in catalysis and regulation of Mycobacterium tuberculosis D-3-phosphoglycerate dehydrogenase. Biochemistry 48:4808–4815 19388702 10.1021/bi900172q PMC2692652

[CR20] Carey AF, Rock JM, Krieger IV, Chase MR, Fernandez-Suarez M, Gagneux S, Sacchettini JC, Ioerger TR, Fortune SM (2018) TnSeq of Mycobacterium tuberculosis clinical isolates reveals strain-specific antibiotic liabilities. PLoS Pathog 14:e1006939 29505613 10.1371/journal.ppat.1006939 PMC5854444

[CR21] Christophe T, Jackson M, Jeon HK, Fenistein D, Contreras-Dominguez M, Kim J, Genovesio A, Carralot JP, Ewann F, Kim EH et al (2009) High content screening identifies decaprenyl-phosphoribose 2’ epimerase as a target for intracellular antimycobacterial inhibitors. PLoS Pathog 5:e1000645 19876393 10.1371/journal.ppat.1000645 PMC2763345

[CR22] Chuenchor W, Doukov TI, Chang KT, Resto M, Yun CS, Gerratana B (2020) Different ways to transport ammonia in human and Mycobacterium tuberculosis NAD(+) synthetases. Nat Commun 11: 16 31911602 10.1038/s41467-019-13845-4 PMC6946656

[CR23] Chung ES, Johnson WC, Aldridge BB (2022) Types and functions of heterogeneity in mycobacteria. Nat Rev Microbiol 20:529–541 35365812 10.1038/s41579-022-00721-0 PMC9681535

[CR24] Conradie F, Diacon AH, Ngubane N, Howell P, Everitt D, Crook AM, Mendel CM, Egizi E, Moreira J, Timm J et al (2020) Treatment of highly drug-resistant pulmonary tuberculosis. N Engl J Med 382:893–902 32130813 10.1056/NEJMoa1901814 PMC6955640

[CR25] Covvey JR, Guarascio AJ (2022) Clinical use of lefamulin: a first-in-class semisynthetic pleuromutilin antibiotic. J Intern Med 291:51–63 34425035 10.1111/joim.13378

[CR26] d’Andrea FB, Poulton NC, Froom R, Tam K, Campbell EA, Rock JM (2022) The essential M. tuberculosis Clp protease is functionally asymmetric in vivo. Sci Adv 8:eabn7943 35507665 10.1126/sciadv.abn7943 PMC9067928

[CR27] Dartois VA, Mizrahi V, Savic RM, Silverman JA, Hermann D, Barry 3rdCE (2025) Strategies for shortening tuberculosis therapy. Nat Med 31:1765–1775 40514466 10.1038/s41591-025-03742-3 PMC12278330

[CR28] DeJesus MA, Gerrick ER, Xu W, Park SW, Long JE, Boutte CC, Rubin EJ, Schnappinger D, Ehrt S, Fortune SM et al (2017) Comprehensive essentiality analysis of the Mycobacterium tuberculosis genome via saturating transposon mutagenesis. MBio 8:e02133–16 28096490 10.1128/mBio.02133-16 PMC5241402

[CR29] Diacon AH, Barry 3rdCE, Carlton A, Chen RY, Davies M, de Jager V, Fletcher K, Koh G, Kontsevaya I, Heyckendorf J et al (2024) A first-in-class leucyl-tRNA synthetase inhibitor, ganfeborole, for rifampicin-susceptible tuberculosis: a phase 2a open-label, randomized trial. Nat Med 30:896–904 38365949 10.1038/s41591-024-02829-7 PMC10957473

[CR30] Early J, Ollinger J, Darby C, Alling T, Mullen S, Casey A, Gold B, Ochoada J, Wiernicki T, Masquelin T et al (2019) Identification of compounds with pH-dependent bactericidal activity against Mycobacterium tuberculosis. ACS Infect Dis 5:272–280 30501173 10.1021/acsinfecdis.8b00256 PMC6371205

[CR31] Evans JC, Trujillo C, Wang Z, Eoh H, Ehrt S, Schnappinger D, Boshoff HI, Rhee KY, Barry 3rdCE, Mizrahi V (2016) Validation of CoaBC as a bactericidal target in the coenzyme a pathway of Mycobacterium tuberculosis. ACS Infect Dis 2:958–968 27676316 10.1021/acsinfecdis.6b00150 PMC5153693

[CR32] Farhat M, Cox H, Ghanem M, Denkinger CM, Rodrigues C, Abd El Aziz MS, Enkh-Amgalan H, Vambe D, Ugarte-Gil C, Furin J et al (2024) Drug-resistant tuberculosis: a persistent global health concern. Nat Rev Microbiol 22:617–635 38519618 10.1038/s41579-024-01025-1

[CR33] Gavalda S, Bardou F, Laval F, Bon C, Malaga W, Chalut C, Guilhot C, Mourey L, Daffe M, Quemard A (2014) The polyketide synthase Pks13 catalyzes a novel mechanism of lipid transfer in mycobacteria. Chem Biol 21:1660–1669 25467124 10.1016/j.chembiol.2014.10.011

[CR34] Godinez-Macias KP, Chen D, Wallis JL, Siegel MG, Adam A, Bopp S, Carolino K, Coulson LB, Durst G, Thathy V et al (2025) Revisiting the Plasmodium falciparum druggable genome using predicted structures and data mining. NPJ Drug Discov 2:3 40066064 10.1038/s44386-025-00006-5 PMC11892419

[CR35] Gold B, Warrier T, Nathan C (2021) A multistress model for high throughput screening against nonreplicating Mycobacterium tuberculosis. Methods Mol Biol 2314:611–635 34235673 10.1007/978-1-0716-1460-0_27

[CR36] Guo H, Courbon GM, Bueler SA, Mai J, Liu J, Rubinstein JL (2021) Structure of mycobacterial ATP synthase bound to the tuberculosis drug bedaquiline. Nature 589:143–147 33299175 10.1038/s41586-020-3004-3

[CR37] Halder A, Samantaray S, Barbade S, Gupta A, Srivastava S (2025) DrugProtAI: a machine learning-driven approach for predicting protein druggability through feature engineering and robust partition-based ensemble methods. Brief Bioinform 26:bbaf330 40627683 10.1093/bib/bbaf330 PMC12236430

[CR38] Hasenoehrl EJ, Rae Sajorda D, Berney-Meyer L, Johnson S, Tufariello JM, Fuhrer T, Cook GM, Jacobs WR Jr, Berney M (2019) Derailing the aspartate pathway of Mycobacterium tuberculosis to eradicate persistent infection. Nat Commun 10:4215 31527595 10.1038/s41467-019-12224-3 PMC6746716

[CR39] Hayden JD, Brown LR, Gunawardena HP, Perkowski EF, Chen X, Braunstein M (2013) Reversible acetylation regulates acetate and propionate metabolism in Mycobacterium smegmatis. Microbiology 159:1986–1999 23813678 10.1099/mic.0.068585-0 PMC3783017

[CR40] Heinrich N, de Jager V, Dreisbach J, Gross-Demel P, Schultz S, Gerbach S, Kloss F, Dawson R, Narunsky K, Matt L et al (2025) Safety, bactericidal activity, and pharmacokinetics of the antituberculosis drug candidate BTZ-043 in South Africa (PanACEA-BTZ-043-02): an open-label, dose-expansion, randomised, controlled, phase 1b/2a trial. Lancet Microbe 6:100952 39793592 10.1016/j.lanmic.2024.07.015

[CR41] Helaine S, Conlon BP, Davis KM, Russell DG (2024) Host stress drives tolerance and persistence: the bane of anti-microbial therapeutics. Cell Host Microbe 32:852–862 38870901 10.1016/j.chom.2024.04.019 PMC11446042

[CR42] Imperial MZ, Nahid P, Phillips PPJ, Davies GR, Fielding K, Hanna D, Hermann D, Wallis RS, Johnson JL, Lienhardt C et al (2018) A patient-level pooled analysis of treatment-shortening regimens for drug-susceptible pulmonary tuberculosis. Nat Med 24:1708–1715 30397355 10.1038/s41591-018-0224-2 PMC6685538

[CR43] Ioerger TR, O’Malley T, Liao R, Guinn KM, Hickey MJ, Mohaideen N, Murphy KC, Boshoff HI, Mizrahi V, Rubin EJ et al (2013) Identification of new drug targets and resistance mechanisms in Mycobacterium tuberculosis. PLoS One 8:e75245 24086479 10.1371/journal.pone.0075245 PMC3781026

[CR44] Janssen S, Upton C, de Jager VR, van Niekerk C, Dawson R, Hutchings J, Kim J, Choi J, Nam K, Sun E et al (2025) Telacebec, a potent agent in the fight against tuberculosis: findings from a randomized, Phase 2 clinical trial and beyond. Am J Respir Crit Care Med 211:1504–1512 40116781 10.1164/rccm.202408-1632OC

[CR45] Johnson EO, LaVerriere E, Office E, Stanley M, Meyer E, Kawate T, Gomez JE, Audette RE, Bandyopadhyay N, Betancourt N et al (2019) Large-scale chemical-genetics yields new M. tuberculosis inhibitor classes. Nature 571:72–78 31217586 10.1038/s41586-019-1315-z

[CR46] Keam SJ (2025) Gepotidacin: first approval. Drugs 85:1479–1485 10.1007/s40265-025-02214-9 40762778

[CR47] Kim JH, O’Brien KM, Sharma R, Boshoff HI, Rehren G, Chakraborty S, Wallach JB, Monteleone M, Wilson DJ, Aldrich CC et al (2013) A genetic strategy to identify targets for the development of drugs that prevent bacterial persistence. Proc Natl Acad Sci USA 110:19095–19100 24191058 10.1073/pnas.1315860110 PMC3839782

[CR48] Kim SK, Dickinson MS, Finer-Moore J, Guan Z, Kaake RM, Echeverria I, Chen J, Pulido EH, Sali A, Krogan NJ et al (2023) Structure and dynamics of the essential endogenous mycobacterial polyketide synthase Pks13. Nat Struct Mol Biol 30:296–308 36782050 10.1038/s41594-022-00918-0 PMC10312659

[CR49] Krieger IV, Sukheja P, Yang B, Tang S, Selle D, Woods A, Engelhart C, Kumar P, Harbut MB, Liu D et al (2025) SuFEx-based antitubercular compound irreversibly inhibits Pks13. Nature 645:755–763 10.1038/s41586-025-09286-3 PMC12962449 40739353

[CR50] Krieger IV, Yalamanchili S, Dickson P, Engelhart CA, Zimmerman MD, Wood J, Clary E, Nguyen J, Thornton N, Centrella PA et al (2024) Inhibitors of the thioesterase activity of Mycobacterium tuberculosis Pks13 discovered using DNA-encoded chemical library screening. ACS Infect Dis 10:1561–1575 38577994 10.1021/acsinfecdis.3c00592 PMC11091879

[CR51] Kumar N, Sharma S, Kaushal PS (2021) Protein synthesis in Mycobacterium tuberculosis as a potential target for therapeutic interventions. Mol Asp Med 81:101002 10.1016/j.mam.2021.101002 34344520

[CR52] Lamont EA, Dillon NA, Baughn AD (2020) The bewildering antitubercular action of pyrazinamide. Microbiol Mol Biol Rev 84:e00070–19 32132245 10.1128/MMBR.00070-19 PMC7062198

[CR53] LaRonde-LeBlanc N, Resto M, Gerratana B (2009) Regulation of active site coupling in glutamine-dependent NAD(+) synthetase. Nat Struct Mol Biol 16:421–429 19270703 10.1038/nsmb.1567

[CR54] Le Guilloux V, Schmidtke P, Tuffery P (2009) Fpocket: an open source platform for ligand pocket detection. BMC Bioinforma 10:168 10.1186/1471-2105-10-168 PMC2700099 19486540

[CR55] Lun S, Xiao S, Zhang W, Wang S, Gunosewoyo H, Yu L-F, Bishai WR (2021) Therapeutic Potential of Coumestan Pks13 Inhibitors for Tuberculosis. Antimicrob Agents Chemother 65:e02190–20 10.1128/AAC.02190-20 PMC8092898 33558290

[CR56] Marrero J, Rhee KY, Schnappinger D, Pethe K, Ehrt S (2010) Gluconeogenic carbon flow of tricarboxylic acid cycle intermediates is critical for Mycobacterium tuberculosis to establish and maintain infection. Proc Natl Acad Sci USA 107:9819–9824 20439709 10.1073/pnas.1000715107 PMC2906907

[CR57] Matsumoto M, Hashizume H, Tomishige T, Kawasaki M, Tsubouchi H, Sasaki H, Shimokawa Y, Komatsu M (2006) OPC-67683, a nitro-dihydro-imidazooxazole derivative with promising action against tuberculosis in vitro and in mice. PLoS Med 3:e466 17132069 10.1371/journal.pmed.0030466 PMC1664607

[CR58] Mishra S, Singh PR, Hu X, Lopez-Quezada L, Jinich A, Jahn R, Geurts L, Shen N, DeJesus MA, Hartman T et al (2025) Candidate transmission survival genome of Mycobacterium tuberculosis. Proc Natl Acad Sci USA 122:e2425981122 40053362 10.1073/pnas.2425981122 PMC11912377

[CR59] MRC TB (1985) Controlled clinical trial of two 6-month regimens of chemotherapy in the treatment of pulmonary tuberculosis. Tanzania/British Medical Research Council Study. Am Rev Respir Dis 131:727–731 3890640 10.1164/arrd.1985.131.5.727

[CR60] Nambi S, Gupta K, Bhattacharyya M, Ramakrishnan P, Ravikumar V, Siddiqui N, Thomas AT, Visweswariah SS (2013) Cyclic AMP-dependent protein lysine acylation in mycobacteria regulates fatty acid and propionate metabolism. J Biol Chem 288:14114–14124 23553634 10.1074/jbc.M113.463992 PMC3656268

[CR61] Nyang’wa BT, Berry C, Kazounis E, Motta I, Parpieva N, Tigay Z, Solodovnikova V, Liverko I, Moodliar R, Dodd M et al (2022) A 24-week, all-oral regimen for rifampin-resistant tuberculosis. N Engl J Med 387:2331–2343 36546625 10.1056/NEJMoa2117166

[CR62] Payne DJ, Gwynn MN, Holmes DJ, Pompliano DL (2007) Drugs for bad bugs: confronting the challenges of antibacterial discovery. Nat Rev Drug Discov 6:29–40 17159923 10.1038/nrd2201

[CR63] Pethe K, Sequeira PC, Agarwalla S, Rhee K, Kuhen K, Phong WY, Patel V, Beer D, Walker JR, Duraiswamy J et al (2010) A chemical genetic screen in Mycobacterium tuberculosis identifies carbon-source-dependent growth inhibitors devoid of in vivo efficacy. Nat Commun 1:57 20975714 10.1038/ncomms1060 PMC3220188

[CR64] Prideaux B, Via LE, Zimmerman MD, Eum S, Sarathy J, O’Brien P, Chen C, Kaya F, Weiner DM, Chen PY et al (2015) The association between sterilizing activity and drug distribution into tuberculosis lesions. Nat Med 21:1223–1227 26343800 10.1038/nm.3937 PMC4598290

[CR65] Priya M, Gupta SK, Koundal A, Kapoor S, Tiwari S, Kidwai S, Sorio de Carvalho LP, Thakur KG, Mahajan D, Sharma D et al (2025) Itaconate mechanism of action and dissimilation in Mycobacterium tuberculosis. Proc Natl Acad Sci USA 122:e2423114122 39841148 10.1073/pnas.2423114122 PMC11789021

[CR66] Puckett S, Trujillo C, Eoh H, Marrero J, Spencer J, Jackson M, Schnappinger D, Rhee K, Ehrt S (2014) Inactivation of fructose-1,6-bisphosphate aldolase prevents optimal co-catabolism of glycolytic and gluconeogenic carbon substrates in Mycobacterium tuberculosis. PLoS Pathog 10:e1004144 24851864 10.1371/journal.ppat.1004144 PMC4031216

[CR67] Raffaelli N, Finaurini L, Mazzola F, Pucci L, Sorci L, Amici A, Magni G (2004) Characterization of Mycobacterium tuberculosis NAD kinase: functional analysis of the full-length enzyme by site-directed mutagenesis. Biochemistry 43:7610–7617 15182203 10.1021/bi049650w

[CR68] Rao SP, Alonso S, Rand L, Dick T, Pethe K (2008) The protonmotive force is required for maintaining ATP homeostasis and viability of hypoxic, nonreplicating Mycobacterium tuberculosis. Proc Natl Acad Sci USA 105:11945–11950 18697942 10.1073/pnas.0711697105 PMC2575262

[CR70] Rath S, Panda S, Sacchettini JC, Berthel SJ (2023) DAIKON: a data acquisition, integration, and knowledge capture web application for target-based drug discovery. ACS Pharm Transl Sci 6:1043–1051 10.1021/acsptsci.3c00034 PMC10353056 37470023

[CR72] Remm S, Earp JC, Dick T, Dartois V, Seeger MA (2022) Critical discussion on drug efflux in Mycobacterium tuberculosis. FEMS Microbiol Rev 46:fuab050 34637511 10.1093/femsre/fuab050 PMC8829022

[CR73] Rodionova IA, Schuster BM, Guinn KM, Sorci L, Scott DA, Li X, Kheterpal I, Shoen C, Cynamon M, Locher C et al (2014) Metabolic and bactericidal effects of targeted suppression of NadD and NadE enzymes in mycobacteria. MBio 5:e00747–13 24549842 10.1128/mBio.00747-13 PMC3944813

[CR74] Sankar J, Chauhan A, Singh R, Mahajan D (2024) Isoniazid-historical development, metabolism associated toxicity and a perspective on its pharmacological improvement. Front Pharm 15:1441147 10.3389/fphar.2024.1441147 PMC11447295 39364056

[CR75] Sarathy J, Blanc L, Alvarez-Cabrera N, O’Brien P, Dias-Freedman I, Mina M, Zimmerman M, Kaya F, Ho Liang HP, Prideaux B et al (2019a) Fluoroquinolone efficacy against tuberculosis is driven by penetration into lesions and activity against resident bacterial populations. Antimicrob Agents Chemother 63:e02516–e02518 30803965 10.1128/AAC.02516-18 PMC6496041

[CR76] Sarathy JP (2024) Molecular and microbiological methods for the identification of nonreplicating Mycobacterium tuberculosis. PLoS Pathog 20:e1012595 39383167 10.1371/journal.ppat.1012595 PMC11463790

[CR77] Sarathy JP, Dartois V (2020) Caseum: a Niche for Mycobacterium tuberculosis drug-tolerant persisters. Clin Microbiol Rev 33:e00159–19 32238365 10.1128/CMR.00159-19 PMC7117546

[CR78] Sarathy JP, Gruber G, Dick T (2019b) Re-understanding the Mechanisms of action of the anti-mycobacterial drug bedaquiline. Antibiotics 8:261 31835707 10.3390/antibiotics8040261 PMC6963887

[CR79] Sarathy JP, Xie M, Jones RM, Chang A, Osiecki P, Weiner D, Tsao WS, Dougher M, Blanc L, Fotouhi N et al (2023) A novel tool to identify bactericidal compounds against vulnerable targets in drug-tolerant M. tuberculosis found in caseum. mBio 14:e0059823 37017524 10.1128/mbio.00598-23 PMC10127596

[CR80] Sassetti CM, Rubin EJ (2003) Genetic requirements for mycobacterial survival during infection. Proc Natl Acad Sci USA 100:12989–12994 14569030 10.1073/pnas.2134250100 PMC240732

[CR81] Sharma R, Hartman TE, Beites T, Kim JH, Eoh H, Engelhart CA, Zhu L, Wilson DJ, Aldrich CC, Ehrt S et al (2023) Metabolically distinct roles of NAD synthetase and NAD kinase define the essentiality of NAD and NADP in Mycobacterium tuberculosis. mBio 14:e0034023 37350592 10.1128/mbio.00340-23 PMC10470730

[CR82] Shi C, Geders TW, Park SW, Wilson DJ, Boshoff HI, Abayomi O, Barry 3rdCE, Schnappinger D, Finzel BC, Aldrich CC (2011) Mechanism-based inactivation by aromatization of the transaminase BioA involved in biotin biosynthesis in Mycobacterium tuberculosis. J Am Chem Soc 133:18194–18201 21988601 10.1021/ja204036t PMC3222238

[CR83] Showalter HD (2020) Recent progress in the discovery and development of 2-nitroimidazooxazines and 6-nitroimidazooxazoles to treat tuberculosis and neglected tropical diseases. Molecules 25:4137 10.3390/molecules25184137 PMC7576498 32927749

[CR84] Silver LL (2011) Challenges of antibacterial discovery. Clin Microbiol Rev 24:71–109 21233508 10.1128/CMR.00030-10 PMC3021209

[CR85] Singh V, Dziwornu GA, Chibale K (2023) The implication of Mycobacterium tuberculosis-mediated metabolism of targeted xenobiotics. Nat Rev Chem 7:340–354 37117810 10.1038/s41570-023-00472-3 PMC10026799

[CR86] Smith CM, Baker RE, Proulx MK, Mishra BB, Long JE, Park SW, Lee HN, Kiritsy MC, Bellerose MM, Olive AJ et al (2022) Host-pathogen genetic interactions underlie tuberculosis susceptibility in genetically diverse mice. Elife 11:e74419 35112666 10.7554/eLife.74419 PMC8846590

[CR87] Telenti A, Philipp WJ, Sreevatsan S, Bernasconi C, Stockbauer KE, Wieles B, Musser JM, Jacobs WR Jr (1997) The emb operon, a gene cluster of Mycobacterium tuberculosis involved in resistance to ethambutol. Nat Med 3:567–570 9142129 10.1038/nm0597-567

[CR88] Tempel W, Rabeh WM, Bogan KL, Belenky P, Wojcik M, Seidle HF, Nedyalkova L, Yang T, Sauve AA, Park HW et al (2007) Nicotinamide riboside kinase structures reveal new pathways to NAD+. PLoS Biol 5:e263 17914902 10.1371/journal.pbio.0050263 PMC1994991

[CR89] VanderVen BC, Fahey RJ, Lee W, Liu Y, Abramovitch RB, Memmott C, Crowe AM, Eltis LD, Perola E, Deininger DD et al (2015) Novel inhibitors of cholesterol degradation in Mycobacterium tuberculosis reveal how the bacterium’s metabolism is constrained by the intracellular environment. PLoS Pathog 11:e1004679 25675247 10.1371/journal.ppat.1004679 PMC4335503

[CR90] Vergnolle O, Xu H, Tufariello JM, Favrot L, Malek AA, Jacobs WR Jr, Blanchard JS (2016) Post-translational acetylation of MbtA modulates mycobacterial siderophore biosynthesis. J Biol Chem 291:22315–22326 27566542 10.1074/jbc.M116.744532 PMC5064009

[CR91] Vilcheze C, Weinrick B, Wong KW, Chen B, Jacobs WR Jr (2010) NAD+ auxotrophy is bacteriocidal for the tubercle bacilli. Mol Microbiol 76:365–377 20199601 10.1111/j.1365-2958.2010.07099.x PMC2945688

[CR92] Wang X, Ahn YM, Lentscher AG, Lister JS, Brothers RC, Kneen MM, Gerratana B, Boshoff HI, Dowd CS (2017) Design, synthesis, and evaluation of substituted nicotinamide adenine dinucleotide (NAD(+)) synthetase inhibitors as potential antitubercular agents. Bioorg Med Chem Lett 27:4426–4430 28827112 10.1016/j.bmcl.2017.08.012 PMC6190683

[CR93] Warner DF, Barczak AK, Gutierrez MG, Mizrahi V (2025) Mycobacterium tuberculosis biology, pathogenicity and interaction with the host. Nat Rev Microbiol 23:788–804 10.1038/s41579-025-01201-x PMC7618155 40588584

[CR94] Wayne LG, Hayes LG (1996) An in vitro model for sequential study of shiftdown of Mycobacterium tuberculosis through two stages of nonreplicating persistence. Infect Immun 64:2062–2069 8675308 10.1128/iai.64.6.2062-2069.1996 PMC174037

[CR95] Wehrli W (1983) Rifampin: mechanisms of action and resistance. Rev Infect Dis 5(Suppl 3):S407–S411 6356275 10.1093/clinids/5.supplement_3.s407

[CR96] Wei JR, Krishnamoorthy V, Murphy K, Kim JH, Schnappinger D, Alber T, Sassetti CM, Rhee KY, Rubin EJ (2011) Depletion of antibiotic targets has widely varying effects on growth. Proc Natl Acad Sci USA 108:4176–4181 21368134 10.1073/pnas.1018301108 PMC3053961

[CR97] Wellington S, Hung DT (2018) The expanding diversity of Mycobacterium tuberculosis drug targets. ACS Infect Dis 4:696–714 29412643 10.1021/acsinfecdis.7b00255

[CR98] W.H.O. (2022) W.H.O. Consolidated guidelines on tuberculosis. Module 4: treatment - drug-resistant treatment, 2022 update. World Health Organization, Geneva 36630546

[CR99] W.H.O. (2025) Global tuberculosis report. World Health Organization

[CR100] Wilburn KM, Montague CR, Qin B, Woods AK, Love MS, McNamara CW, Schultz PG, Southard TL, Huang L, Petrassi HM et al (2022) Pharmacological and genetic activation of cAMP synthesis disrupts cholesterol utilization in Mycobacterium tuberculosis. PLoS Pathog 18:e1009862 35134095 10.1371/journal.ppat.1009862 PMC8856561

[CR101] Wilson C, Ray P, Zuccotto F, Hernandez J, Aggarwal A, Mackenzie C, Caldwell N, Taylor M, Huggett M, Mathieson M et al (2022) Optimization of TAM16, a benzofuran that inhibits the thioesterase activity of Pks13; evaluation toward a preclinical candidate for a novel antituberculosis clinical target. J Med Chem 65:409–423 34910486 10.1021/acs.jmedchem.1c01586 PMC8762665

[CR102] Wilson R, Kumar P, Parashar V, Vilcheze C, Veyron-Churlet R, Freundlich JS, Barnes SW, Walker JR, Szymonifka MJ, Marchiano E et al (2013) Antituberculosis thiophenes define a requirement for Pks13 in mycolic acid biosynthesis. Nat Chem Biol 9:499–506 23770708 10.1038/nchembio.1277 PMC3720791

[CR103] Woong Park S, Klotzsche M, Wilson DJ, Boshoff HI, Eoh H, Manjunatha U, Blumenthal A, Rhee K, Barry 3rdCE, Aldrich CC et al (2011) Evaluating the sensitivity of Mycobacterium tuberculosis to biotin deprivation using regulated gene expression. PLoS Pathog 7:e1002264 21980288 10.1371/journal.ppat.1002264 PMC3182931

[CR104] Xia F, Zhang H, Yang H, Zheng M, Min W, Sun C, Yuan K, Yang P (2023) Targeting polyketide synthase 13 for the treatment of tuberculosis. Eur J Med Chem 259:115702 37544185 10.1016/j.ejmech.2023.115702

[CR105] Xu H, Hegde SS, Blanchard JS (2011) Reversible acetylation and inactivation of Mycobacterium tuberculosis acetyl-CoA synthetase is dependent on cAMP. Biochemistry 50:5883–5892 21627103 10.1021/bi200156t PMC3125470

[CR106] Yang H, Sha W, Liu Z, Tang T, Liu H, Qin L, Cui Z, Chen J, Liu F, Zheng R et al (2018) Lysine acetylation of DosR regulates the hypoxia response of Mycobacterium tuberculosis. Emerg Microbes Infect 7:34 29559631 10.1038/s41426-018-0032-2 PMC5861037

[CR107] Yang M, Wang Y, Chen Y, Cheng Z, Gu J, Deng J, Bi L, Chen C, Mo R, Wang X et al (2015) Succinylome analysis reveals the involvement of lysine succinylation in metabolism in pathogenic Mycobacterium tuberculosis. Mol Cell Proteom 14:796–811 10.1074/mcp.M114.045922 PMC4390261 25605462

[CR108] Zaidi SMA, Coussens AK, Seddon JA, Kredo T, Warner D, Houben R, Esmail H (2023) Beyond latent and active tuberculosis: a scoping review of conceptual frameworks. EClinicalMedicine 66:102332 38192591 10.1016/j.eclinm.2023.102332 PMC10772263

[CR109] Zhang W, Lun S, Wang SS, Cai YP, Yang F, Tang J, Bishai WR, Yu LF (2022) Structure-based optimization of coumestan derivatives as polyketide synthase 13-thioesterase(Pks13-TE) inhibitors with improved hERG profiles for Mycobacterium tuberculosis treatment. J Med Chem 65:13240–13252 36174223 10.1021/acs.jmedchem.2c01064

[CR110] Zumla AI, Gillespie SH, Hoelscher M, Philips PP, Cole ST, Abubakar I, McHugh TD, Schito M, Maeurer M, Nunn AJ (2014) New antituberculosis drugs, regimens, and adjunct therapies: needs, advances, and future prospects. Lancet Infect Dis 14:327–340 24670627 10.1016/S1473-3099(13)70328-1

