# Supplementary material for: Integrating chemical, genetic, and feasibility assessments for anti-tubercular target validation
Source: EMBO Mol Med. 2026 Apr 7;18(5):1495–507. doi: 10.1038/s44321-026-00415-7 (PMC13179349; doi:10.1038/s44321-026-00415-7)
Supplement: Supplementary file 1 — Table EV1 [file 44321_2026_415_MOESM1_ESM.docx]

# ****Table EV1****

| Target | Function | Chemical Validation | Genetic Validation |
| --- | --- | --- | --- |
| DNA Replication and Repair | | | |
| DnaE1 | DnaE1 is the α subunit of DNA polymerase III and responsible for chromosomal DNA replication. | The natural product nargenicin binds to DnaE1 in a DNA-dependent manner, as shown by cryo-EM. On-target whole-cell activity in *Mtb* is supported by (i) a transcriptional profile dominated by a DNA damage response; (ii) cell elongation consistent with replication inhibition (de Wet *et al*, 2020); and (iii) selective inhibition of DNA replication in macromolecular incorporation assays (Chengalroyen *et al*, 2022) | Genetic inactivation of *dnaE1* is growth inhibitory *in vitro* (Chengalroyen *et al.*, 2022) and *dnaE1* is among the *Mtb* genes most vulnerable to partial genetic inactivation (Bosch *et al*, 2021). |
| DnaN | DnaN is the β-sliding clamp (processivity factor) of DNA polymerase III. It co-localizes polymerase and repair enzymes with DNA via protein-protein interactions at a specific site in the dimer. It is essential for DNA replication and DNA repair. | The macrocyclic peptide class of natural products – Griselimycins and Mycoplanecins – bind and inhibit the interaction of the sliding clamp and DNA polymerase III. These compounds have potent and antibacterial activity against *Mtb* and other mycobacteria. X-ray co-crystal structures with DnaN of *Mtb* and *E. coli* have been solved (e.g., 5AGU, 5AGV, 5AH4, 5AH2, 8CIZ). Griselimycins are inactive against non-replicating *Mtb* under hypoxic conditions (Fu *et al*, 2024; Kling *et al*, 2015). Griselimycins are efficacious in mouse models of TB (Kling *et al.*, 2015). Resistance arises at low frequency with high fitness cost through chromosomal amplification of *dnaN* and flanking regions. This amplification rapidly reverses in the absence of drug pressure (Kling *et al.*, 2015). | *DnaN* encodes an essential replisome component (Bosch *et al.*, 2021; DeJesus *et al*, 2017). Conditional knockout strains have been generated in *M. smegmatis* (*Msm*) and show complementation by *Mtb* *dnaN* (Mulye & Jain, 2025). |
| Gyrase (GyrA, GyrB) | DNA gyrase is a tetrameric (GyrA_2_B_2_) type II DNA topoisomerase that controls DNA topology. It introduces transient double‑stranded DNA breaks, passes another DNA segment through the break, and then reseals the DNA. | Fluoroquinolones, which are cidal and used clinically for TB, stabilize the cleaved DNA–enzyme intermediate and cause double‑stranded DNA breaks (Aldred *et al*, 2014). Multiple non-fluoroquinolone GyrA inhibitors that bind distinct ligand-binding sites also inhibit *Mtb* growth *in vitro* and *in vivo* (Basarab *et al*, 2022; Blanco *et al*, 2015; Gedeon *et al*, 2024; Imai *et al*, 2022). Coumarins such as novobiocin inhibit the ATPase activity of the GyrB subunits. Several natural products and small molecule inhibitors targeting GyrB inhibit *Mtb* growth (Chopra *et al*, 2012; Gl *et al*, 2020; Kashyap *et al*, 2018; Stokes *et al*, 2020). | *GyrA* and *gyrB* are essential, and their genetic silencing is cidal (Bosch *et al.*, 2021; Choudhary *et al*, 2019; Rock *et al*, 2017). Genetic silencing of gyrase can, however, induce drug tolerant persisters (Choudhary *et al.*, 2019). |
| Transcription and mRNA Metabolism | | | |
| RNA polymerase (RpoA, RpoB, RpoC, RpoZ) | The RNAP core enzyme is encoded by *rpoA* (α-subunit), *rpoB* (β-subunit), *rpoC* (β’-subunit) and *rpoZ* (ω-subunit), forming the α_2_ββ’ω complex | Several classes of antibiotics target RNA polymerase enzyme, including the rifamycins, soranigicin, kanglemycins, didoxamycins, and myxopyronins. Rifamycins are naturally derived antibiotics and a critical component in standard TB therapy. They bind to a site on the β-subunit adjacent to the active center and prevent the transition from initiation to elongation (Kirsch *et al*, 2022). Most rifamycin resistance mutations map to *rpoB*, with the most prevalent clinical substitutions (e.g., S450L, H445Y, D435V) accounting for most rifampin-resistant TB. These single-amino-acid changes abolish rifamycin activity (Molodtsov *et al*, 2017; Mosaei & Zenkin, 2020).  Ongoing efforts aim to identify novel small molecules to address challenges associated with long‑term rifamycin use and clinical resistance. This includes work on aroyl‑aryl‑phenylalanine amides (AAPs), potent inhibitors that bind the β‑subunit at a site distinct from rifamycins (Lin *et al*, 2017). | Bacterial RNA polymerase is an essential enzyme and is indispensable for growth (Bosch *et al.*, 2021; DeJesus *et al.*, 2017). Conditional knockouts and hypomorphs are available to study on-target activity of compounds in *Mtb*. |
| DosR | DosR is a DNA binding protein that regulates a regulon of genes induced in response to hypoxia. Its DNA binding activity is positively regulated by phosphorylation via the sensor histidine kinases DosS and DosT, but can also be negatively regulated by post-translational modifications like reversible acetylation of lysine residue 182 (Sivaramakrishnan & de Montellano, 2013; Wisedchaisri *et al*, 2008). | A high-throughput chemical screen using a fluorescent reporter strain identified two chemical scaffolds that exhibited evidence of dose-dependent inhibition and biochemical evidence of on-target binding to DosR and DosS, respectively. This was demonstrated by inhibition of DosR DNA binding and DosS protein conformation and overexpression-mediated resistance. Combinations of these inhibitors additionally demonstrated evidence of *in vitro* synergy (Zheng *et al*, 2020). | A *dosRS* deletion mutant of H37Rv showed modest attenuated in C57BL/6, BALB/C, C3HeB/FeJ mice, New Zealand white rabbits and outbred Hartley Guinea pigs, though this attenuation was due, in part, to a loss of DosS, rather than DosR (Converse *et al*, 2009; Gautam *et al*, 2015). A study in macaques however showed a defect in the ability of a DosR-specific deletion to persist that could be genetically complemented by DosR alone (Mehra *et al*, 2015). |
| GpsI | GpsI functions both as a polynucleotide phosphorylase (PNPase) and as a guanosine pentaphosphate synthetase (GpsI), with roles in RNA processing, rRNA maturation, and regulation of the stringent response. | A biphenyl carboxamide (named X1) with activity against *Mtb* was identified in a phenotypic screen and shown to be susceptible to resistance mutations in *gpsI* (Dal Molin et al, 2019). Recently, X1 was demonstrated (i) to inhibit both poly(A) degradation and poly(A) synthesis by GpsI *in vitro*, and (ii) to bind between the RNA-binding domain and the catalytically active RNAse PH-like domain 2 (Griesser *et al*, 2025). | *GpsI* has been classified as an *in vitro* essential *Mtb* gene by TnSeq and CRISPRi (Bosch *et al.*, 2021; DeJesus *et al.*, 2017). |
| Rho | Rho is an essential ATP-dependent helicase mediating transcription termination. | The natural product bicyclomycin (BCM) acts as an antibiotic against many Gram-negative and some Gram-positive bacteria via inhibition of Rho. However, Rho of *Mtb* is intrinsically resistant to BCM (Saridakis *et al*, 2022) and efforts to identify progressible inhibitors for mycobacterial Rho have failed so far. | Genetic depletion of Rho leads to a rapid loss of viability for replicating and nonreplicating *Mtb* and strongly attenuates *Mtb* in acute and chronic mouse infections (Botella *et al*, 2017). |
| VirS | VirS is an AraC/XylS-family transcriptional regulator. It is positioned divergently from the acid-inducible *mymA* operon, which it directly activates. VirS orchestrates a broader transcriptional response to acidic stress, including genes involved in central metabolism, cell-envelope remodeling, efflux/ion transport and detoxification (Singh *et al*, 2003; Singh *et al*, 2019a). | A structure-guided/virtual screen followed by biochemical and cellular validation identified multiple small molecules that inhibit VirS DNA binding. These inhibitors reduce *Mtb* growth *in vitro* and diminish intracellular growth in macrophages (Singh *et al.*, 2019a). Whether these compounds affect *Mtb* solely via engagement of VirS remains to be demonstrated.  VirS is also the target of some ethionamide (ETH) boosters, such as SMARt751. ETH is a pro-drug that is activated by EthA and MymA (Grant *et al*, 2016). SMARt751 interacts with VirS *in vitro* and causes upregulation of the VirS regulon in *Mtb*. Mutations that cause resistance to the ETH-SMARt751 combination map to *mymA* and genes regulating the expression of *mymA*, including *virS* (Flipo *et al*, 2022). A derivative of SMARt751 (referred to as Alpibectir or BL-GSK098) is in clinical evaluation (Pieren *et al*, 2024). | *VirS* is dispensable for growth of *Mtb* *in vitro* (Bosch *et al.*, 2021; DeJesus *et al.*, 2017). Disruption of *virS* alters mycolic acid composition and cell-envelope properties, compromises maintenance of intrabacterial pH under acid stress and within IFN-γ–activated macrophages, impairs the block of phagosome–lysosome fusion, reduces intracellular survival, and attenuates dissemination *in vivo* (Singh *et al.*, 2003; Singh *et al.*, 2019a). |
| Translation | | | |
| LeuS | LeuS catalyzes the attachment of leucine to its corresponding tRNA, an essential step in protein synthesis. | Ganfeborole (GSK3036656) is a 3-amino 4-chlorobenzoxaborole that selectively inhibits *Mtb* LeuS, resulting in potent cellular activity across DS, MDR and XDR strains of *Mtb* (Li *et al*, 2017). Crystal structures of compounds from this series bound to LeuS and the isolation of resistant mutants confirm an on-target mode of action (Palencia *et al*, 2016). Ganfeborole has recently completed Phase 2a clinical profiling (Diacon *et al*, 2024) and remains in clinical development. | *LeuS* has been classified as an *in vitro* essential *Mtb* gene by TnSeq and CRISPRi (Bosch et al., 2021; DeJesus et al, 2017). |
| LysS | LysS catalyzes the attachment of lysine to its corresponding tRNA, a critical step in protein synthesis. | DDU209 (compound 8) is a potent LysRS inhibitor (IC_50_ = 50 nM) with whole cell activity (intracellular MIC_90_ = 40 nM). Crystallography shows that the inhibitor binds in the ATP pocket (7QI8). On-target cellular f action was confirmed by resistant mutants and reduced potency in *Mtb* strains overexpressing LysRS (Davis *et al*, 2025; Green *et al*, 2022). Efficacy was observed in acute and chronic models of infection, with the early lead DDU774 (compound 5) can substitute for linezolid in preliminary combination studies (Davis *et al.*, 2025; Green *et al.*, 2022). Representatives from the series are active in a non-replicating and caseum MBC assay (unpublished data). | *LysS* has been classified as an *in vitro* essential *Mtb* gene by TnSeq and CRISPRi (Bosch et al., 2021; De-Jesus et al, 2017)*.* |
| AspS | AspS catalyzes the attachment of aspartic acid to its corresponding tRNA, an essential step in protein synthesis. | The crystal structure of apo *Msm* AspS (PDB: 4O2D) was reported in 2014 (Gurcha *et al*, 2014). Subsequently, several biochemically validated *Mtb* AspS inhibitors were identified using a target-based whole-cell screening assay in *M. bovi*s BCG genetically modified to constitutively express the *Mtb* *aspS* (Soto *et al*, 2018). A screen using a zebrafish infection model identified an additional chemotype (Habjan *et al*, 2021). No further progression of any of these AspS hits has been reported to date. | *AspS* has been classified as an *in vitro* essential *Mtb* gene by TnSeq and CRISPRi (Bosch et al., 2021; De-Jesus et al, 2017). |
| MetS | MetS catalyzes the attachment of methionine to its corresponding tRNA, a critical step in protein synthesis. | The crystal structure of *Mtb* MetRS in complex with its catalytic intermediate (methionyl adenylate) has been resolved (PDB: 6AX8). MetS has attracted interest as an antibacterial target (Green *et al*, 2009) and multiple modelling and machine learning studies have been reported (*e.g.* by Volynets and colleagues (Volynets *et al*, 2022) but no active inhibitors have been reported to date. | *MetS* has been classified as an *in vitro* essential *Mtb* gene by TnSeq and CRISPRi (Bosch et al., 2021; De-Jesus et al, 2017). |
| Ribosome | Ribosomes are the protein-synthesizing factories of the cell, providing the platform on which amino acids are polymerized in a template-dependent fashion to form polypeptide chains. | The ribosome is the target for multiple classes of drugs in clinical use and compounds in development, including aminoglycosides (Ahuja *et al*, 2012; Campbell *et al*, 2011; Council, 1948; Zaunbrecher *et al*, 2009), tuberactinomycins (Maus *et al*, 2005; Stanley *et al*, 2010), oxazolidinones (Aono *et al*, 2022; Conradie *et al*, 2020; Furin *et al*, 2016; Gordon *et al*, 2022; Kim *et al*, 2022; Wallis *et al*, 2014), spectinomycin derivatives (Bauman *et al*, 2024; Lee *et al*, 2014; Temrikar *et al*, 2023), and macrolides (Zhang *et al*, 2023b). | Most genes encoding ribosomal proteins have been classified as essential by TnSeq and CRISPRi (Bosch *et al.*, 2021; DeJesus *et al.*, 2017). |
| LysA | LysA catalyzes decarboxylation of meso-diaminopimelate (meso-DAP) to produce L-lysine, contributing to both cell wall and amino acid biosynthesis. | Small molecules that effectively engage LysA in *Mtb* remain to be developed. | *LysA* is required for growth of *Mtb* in standard media, but the gene can be deleted when lysine is provided (Abrahams *et al*, 2012). The resulting lysine auxotroph was cleared rapidly from C57BL/6 mice (Pavelka *et al*, 2003). |
| TrpA, TrpB | TrpA and TrpB form tryptophan synthetase, a heterotetrametric enzyme complex that catalyzes a step in tryptophan biosynthesis. | Three different chemical series have been reported that target TrpAB (Abrahams *et al*, 2017; Wellington *et al*, 2017). They were identified by phenotypic screening, with mechanisms of action establish via isolation of resistant mutants, tryptophan supplementation (which eliminates growth inhibition) and co-crystal structures. One series has demonstrated activity in a murine model of infection while the other had activity in a zebrafish model. | Genetic analyses have shown that *trpA* and *trpB* are essential *in vitro* (Bosch *et al.*, 2021; DeJesus *et al.*, 2017) and during infection (Wellington *et al.*, 2017). |
| ***Protein Degradation*** | | | |
| ClpP1, ClpP2 | The ClpP1P2 complex is an essential intracellular serine protease that maintains protein homeostasis by degrading abnormal, misfolded, or regulatory proteins. It associates with regulatory ATPases (ClpC1 and ClpX) to form a complete, ATP-dependent degradation machine. | Known inhibitors include peptide boronates, such as the anti-cancer drug bortezomib (Akopian *et al*, 2015) and the acyldepsipeptides (ADEPs) (Schmitz *et al*, 2023). Silencing of *clpP1P2* sensitizes *M. bovis* BCG to growth inhibition by ADEPs (Famulla *et al*, 2016). | TnSeq and CRISPRi have established essentiality of *clpP1P2* for growth *in vitro* and in macrophages, and a high degree of vulnerability associated with down-regulation (Bosch *et al.*, 2021; DeJesus *et al.*, 2017; Lunge *et al*, 2020). |
| ClpC1 | ClpC1 is an ATPase that associates with the proteolytic domains, ClpP1 and ClpP2, and together they are responsible for waste protein degradation within the cell. | Natural products such as Ecumicin, Cyclomarin A and Rufomycin bind ClpC1 with high affinity and inhibit bacterial growth (Choules *et al*, 2019; Gao *et al*, 2015; Schmitt *et al*, 2011). Crystal structures of these natural products bound to ClpC1 have been solved (e.g., 9DIN, 6CN8, 8A8V, 8A8W, 6PBS, 3WDC). Rufomycin and CymA resistant mutants carry mutations in ClpC1. *In vivo* administration of ecumicin reduces growth of *Mtb* in mice (Gao *et al.*, 2015). | *ClpC1* is essential for growth of *Mtb* *in vitro* and in macrophages (Bosch *et al.*, 2021; DeJesus *et al.*, 2017; Lunge *et al.*, 2020). |
| PrcB, PrcA | PrcB and PrcA form the core proteasome complext that degrades proteins tagged with the prokaryotic ubiquitin-like protein Pup. | Determination of the mechanism of inhibition by peptidyl boronates (Lin *et al*, 2009) was followed by determination of peptidyl species specificity (Lin *et al*, 2008) and then by demonstration of species-selective cidal activity of oxathiazolone inhibitors against non-replicating *Mtb* with solution of the structure of the co-crystal (Lin *et al.*, 2009), then by N,C-capped dipeptides (Lin *et al*, 2013) and their co-crystals (Hsu *et al*, 2017) and phenylimidazole based peptidomimetics and their co-crystals (Zhan *et al*, 2019). Co-crystal structures were solved for all 5 series. Compounds in each series exhibit anti-*Mtb* activity. Oxathiazolones (Lin *et al.*, 2008) and macrocyclic peptides (Zhang *et al*, 2021) were confirmed to inhibit the proteasome within *Mtb*. | Conditional silencing of *prcBA* killed *Mtb* during the chronic phase of infection in mice (Gandotra *et al*, 2007). |
| ***Protein Secretion*** | | | |
| LepB | LepB is the sole signal peptidase in mycobacteria (Ollinger *et al*, 2012). It cleaves the signal peptides from proteins exported via the general secretory (Sec) pathway (Paetzel *et al*, 2002). | MD3, a known signal peptidase inhibitor, has weak activity against *M. tuberculosis*, which appears to be via inhibition of secretion (Barbosa *et al*, 2002). Several compound series identified against a LepB hypomorph were identified; however, the published series do not appear to target LepB directly (Bonnett *et al*, 2023; Bonnett *et al*, 2016). | *LepB* is essential *in vitro* under standard conditions against replicating and non-replicating bacilli (Bosch *et al.*, 2021; DeJesus *et al.*, 2017). Gene silencing confirms that *lepB* is vulnerable, but that depletion of LepB requires several generations before an effect on growth is seen. |
| SecA1 | SecA1 is an essential ATPase in the canonical Sec pathway that mediates protein export across the cytoplasmic membrane. It associates peripherally with the SecYEG translocon and uses ATP hydrolysis to drive the translocation of unfolded preproteins through the membrane channel (Swanson *et al*, 2015). SecA1 recognizes the signal peptides and mature domains of preproteins and interacts with the SecB chaperone to ensure efficient protein export (Economou & Wickner, 1994; Hartl *et al*, 1990). | No *Mtb*–validated SecA1 inhibitors have been reported so far. Although SecA inhibitors such as sodium azide  (Klein *et al*, 1994; Oliver *et al*, 1990; Segers & Anne, 2011), CJ-21058 (Sugie *et al*, 2002), pannomycin (Parish *et al*, 2009), SEW-05929 and HTS-12302 (Li *et al*, 2008), rose bengal (Hsieh *et al*, 2014; Huang *et al*, 2012), and thiouracils (Chen *et al*, 2010; Cui *et al*, 2017) demonstrate SecA inhibition in other bacterial species, there is no direct evidence for on-target activity in *Mtb*. The decoquinate derivative RMB041 (Knoll *et al*, 2022) exhibits potent whole-cell activity against *Mtb*, but mechanistic and computational studies implicate alternative targets, and do not validate SecA1 as its primary mode of action. | *SecA1* is highly conserved and essential for growth of *Mtb* (Bosch *et al.*, 2021; DeJesus *et al.*, 2017). Conditional depletion of SecA1 in *Mtb* impaired growth, reduced secretion of SecA1‐dependent substrates, and increased membrane permeability (Rigel *et al*, 2009). |
| SecY, SecE, SecG | The SecYEG complex forms the core protein-conducting channel of the Sec machinery in the cytoplasmic membrane. It mediates the translocation of unfolded preproteins across the cytoplasmic membrane or their insertion into the membrane (Economou & Wickner, 1994; Feltcher *et al*, 2010). Within this complex, SecY forms the central pore through which preproteins pass, while SecE1 and SecG stabilize and regulate the conformation of the channel during secretion (Miller *et al*, 2017; Tam *et al*, 2005). Together with SecA1 the SecYEG complex drives secretion of essential cell-wall and virulence proteins in *Mtb* (Driessen & Nouwen, 2008). | In *Mtb*, SecYEG is chemically validated primarily via Amycobactin, a natural product that SecY and exhibits potent activity against replicating and non-replicating *Mtb* (Quigley *et al*, 2020). Other inhibitors, such as Eeyarestatin‑24 (ES24) (Schafer *et al*, 2023; Steenhuis *et al*, 2021) act on SecYEG in other bacteria but are not validated in *Mtb*. Decatransin (Junne *et al*, 2015) inhibits the eukaryotic Sec61 translocon (eukaryotic SecYEG homolog) but does not act on bacteria and has no demonstrated antibacterial activity or on‑target inhibition of bacterial SecYEG. | The SecYEG complex in *Mtb* is essential for protein export. *SecY* and *SecE* genes are indispensable for bacterial viability, as demonstrated by loss-of-function and depletion studies causing severe secretion defects and loss of growth. *SecG* is nonessential but increases translocation efficiency, and its deletion reduces protein export performance, marked with a fitness defect, without lethal effects (DeJesus *et al.*, 2017; Miller *et al.*, 2017; Veenendaal *et al*, 2004). *SecY* and *secE* are also highly vulnerable to partial genetic inactivation (Bosch *et al.*, 2021). |
| ***Peptidoglycan*** | | | |
| MurA | After the formation of uridine diphosphate (UDP)-N-acetylglucosamine (GlcNAc), several Mur enzymes (MurA–F) contribute to peptidoglycan (PG) synthesis. MurA catalyzes the first committed step by transferring an enolpyruvate residue from phosphoenolpyruvate to UDP-GlcNAc. | MurA is validated in other pathogens as the target of the broad-spectrum antibiotic fosfomycin (Kim & Lees, 2025). However, *Mtb* MurA is insensitive to fosfomycin and small molecule inhibitors that inhibit growth of *Mtb* via engagement of MurA remain to be discovered. | *MurA* has been classified as an *in vitro* essential *Mtb* gene by TnSeq and CRISPRi (Bosch *et al.*, 2021; DeJesus *et al.*, 2017). Inducible depletion of MurA resulted in reduced fitness in a mouse model of TB (Koh *et al*, 2022). |
| MurI | MurI is glutamate racemase and converts L‑glutamate to D‑glutamate for incorporation into peptidoglycan (Maitra *et al*, 2019). It also exhibits a moonlighting, racemase‑independent activity that binds GyrA and inhibits DNA gyrase DNA binding (Sengupta *et al*, 2008). | β‑Chloro‑D‑alanine (BCDA) is a mechanism‑based, irreversible MurI inhibitor with antitubercular activity. Treatment of *Mtb* with BCDA leads to (i) formation of a BCDA‑modified MurI peptide (consistent with covalent engagement of MurI) and (ii) causes selective changes in peptidoglycan precursor pools consistent with blockade of D‑Glu incorporation. Supplementation with D‑Glu (2 mM) increases the BCDA MIC for H37Rv by ~32‑fold and rescues metabolomic phenotypes without altering intracellular BCDA levels; overexpression of a surrogate glutamate racemase in *Mtb* H37Rv increased the BCDA MIC ~16‑fold (Prosser *et al*, 2016). | Attempts to delete *murI* in *Mtb* failed unless a second copy of *murI* was supplied (Morayya *et al*, 2015). *MurI* has also been classified as an *in vitro* essential *Mtb* gene by TnSeq and CRISPRi (Bosch *et al.*, 2021; DeJesus *et al.*, 2017) |
| ***Nucleotide Biosynthesis*** | | | |
| GuaB2 | GuaB2 is inosine monophosphate dehydrogenase (IMPDH) and catalyzes the conversion of inosine monophosphate (IMP) to xanthosine monophosphate (XMP) in the purine biosynthesis pathway. Of the three IMPDH homologs in *Mtb*, GuaB2 is the only essential, catalytically active enzyme. | Multiple inhibitors of *Mtb* GuaB2 with activity against wildtype *Mtb* have been described (Cox *et al*, 2016; Park *et al*, 2017; Singh *et al*, 2017; Singh *et al*, 2019b). Evidence for on-target activity is based on (i) an MIC upshift in *M. bovis* BCG overexpressing *guaB2* (Cox et al., 2016); (ii) resistant mutations within *guaB2* (Singh *et al.*, 2017) or its promoter (Park et al., 2017); (iii) hypersensitization by *guaB2* knockdown (Singh *et al.*, 2017; Singh *et al.*, 2019b); and (iv) metabolite rescue of *Mtb* by exogenous guanine (Park *et al.*, 2017; Singh *et al.*, 2017).  Advanced indazole sulfonamide lead compounds failed to show efficacy in acute or chronic mouse models. Moreover, measurement of guanine levels in resected lung tissue from *Mtb*-infected rabbits and human TB patients revealed high levels of guanine (0.2-5 mM) suggested the potential for metabolic bypass further tempering enthusiasm for this target (Park *et al.*, 2017).  However, more recent studies of nucleobase levels in resected lung tissue from TB patients found much lower concentrations of guanine (~5 μM) (Lamprecht *et al*, 2025). | *Mtb* is vulnerable to partial genetic depletion of *guaB2* (Bosch *et al.*, 2021) and genetic inactivation of *guaB2* prevents *Mtb* from establishing an infection in mice (Singh *et al.*, 2017). |
| PurF | PurF catalyzes the first and committed step of *de novo* purine biosynthesis, producing phosphoribosylamine (PRA) from phosphoribosylpyrophosphate (PRPP) via the conversion of glutamine to glutamate. | JNJ-6640 is a potent inhibitor of PurF (IC_50_ = 1nM) with *in vitro* whole cell activity (MIC_90_ = 8.6 ± 3.9 nM, MBC_99.9_ = 140 ± 63 nM). *In vivo* efficacy was achieved using both acute and chronic mouse infection models. JNJ-6640 was also shown to contribute to the BPa backbone regimen *in vitro* and *in vivo*.  That JNJ-6640 exerts its effects on *Mtb* strains is supported by (i) the mapping of resistance mutations to *purF*, (ii) inhibition of de novo purine biosynthesis in *Mtb*, and (iii) potentiation of JNJ-6640 activity in response to partial silencing of *purF* *(*Lamprecht et al., 2025). | *PurF* has been classified as an *in vitro* essential *Mtb* gene by TnSeq and CRISPRi (Bosch *et al.*, 2021; DeJesus *et al.*, 2017). *PurF* transposon mutants (isolated in media supplemented with hypoxanthine and thiamine) show a strong growth defect in standard media and are severely attenuated in mice (Block *et al*, 2024). |
| PyrG | PyrG is CTP synthetase and catalyzes the ATP-dependent conversion of UTP to CTP by transferring an amide nitrogen from glutamine or ammonia | Phenotypic screening identified two thiophenecarboxamide derivatives that require activation by EthA and are active against replicating and nonreplicating *Mtb*. Resistance mutations mapped to *ethA* and *pyrG* and an active metabolite of one thiophenecarboxamide derivative was found to be a competitive inhibitor of PyrG. Metabolomic studies confirmed that these compounds cause deregulation of nucleotide metabolism, consistent with inhibition of PyrG (Mori *et al*, 2005). Follow up work identified PanK as an additional target for these compounds (Chiarelli *et al*, 2018). Biochemical screens against recombinant PyrG identified thiozole derivates that were more potent against a *pyrG* conditional knockdown (Esposito *et al*, 2017). | *PyrG* has been classified as an *in vitro* essential *Mtb* gene by TnSeq and CRISPRi (Bosch *et al.*, 2021; DeJesus *et al.*, 2017). |
| Isoprenoid Biosynthesis | | | |
| Dxs1 | Dxs1 catalyzes the first and rate-limiting step of the methylerythritol phosphate (MEP) pathway, one of two routes for isoprenoid biosynthesis in bacteria. This pathway is essential for growth of *Mtb*, as the alternative mevalonate pathway is absent. The Dxs1 enzyme is also involved in thiamine diphosphate (cofactor) biosynthesis (Gierse *et al*, 2021; Zhang *et al*, 2009). | Several inhibitor classes have demonstrated varying potencies against *Mtb* Dxs1. Thiamine derivatives (hDP analogs) act as competitive inhibitors mimicking thiamine diphosphate (ThDP), showing sub-μM to low micromolar inhibition of *Mtb* Dxs1 (Masini *et al*, 2015).  Butylacetyl phosphonate (BAP) is a selective mechanism-based inhibitor that binds to the pyruvate site of *Mtb* Dxs1. However, despite effective enzyme inhibition at low micromolar concentrations, BAP’s antibacterial activity is limited due to poor cellular uptake across the mycobacterial cell envelope (Smith *et al*, 2014).  Ligand-based virtual screening has identified novel drug-like molecules, showing promising enzymatic and whole-cell activity against drug-resistant *Mtb* strains, with favorable stability and low resistance development (Zhu *et al*, 2022).  That *Mtb* growth inhibition is due to engagement of Dxs1 remains to be demonstrated. | *Dxs1* has been classified as essential by TnSeq and transcriptional suppression of *dxs1* markedly hindered *Mtb* growth, confirming its essential role for bacterial viability. The growth defect could not be alleviated by adding MEP pathway intermediates, demonstrating that *Mtb* depends on *dxs1* for isoprenoid biosynthesis in the absence of alternative salvage routes. Overexpression of *dxs1* was inhibitory to *Mtb* growth. Furthermore, *dxs1*-depleted strains exhibited heightened susceptibility to first-line tuberculosis drugs (isoniazid, rifampicin, and ethambutol) (Bosch *et al.*, 2021; Brown *et al*, 2010; DeJesus *et al.*, 2017; Rani & Surolia, 2024). |
| Lipid Transport |  |  |  |
| EfpA | EfpA is a membrane protein that belongs to the major facilitator superfamily. Recent studies suggest that it primarily functions as a lipid transporter and it has been implicated in resistance to multiple drugs (Li *et al*, 2024; Wang *et al*, 2024). | EfpA inhibitors were identified in chemical genetic screens (Johnson *et al*, 2019; Johnson *et al*, 2020) and the structures of EfpA in complex with two inhibitors have been determined (Khandelwal *et al*, 2025; Li *et al.*, 2024; Wang *et al.*, 2024). On-target activity of these inhibitors in *Mtb* is supported by resistance mutations in *efpA* (Johnson *et al.*, 2019; Khandelwal *et al.*, 2025). | *EfpA* has been classified as an *in vitro* essential *Mtb* gene by TnSeq and CRISPRi (Bosch *et al.*, 2021; DeJesus *et al.*, 2017). |
| Central Carbon Metabolism | | | |
| PckA | PckA (or PEPCK) is a GTP-dependent phophoenolpyruvate (PEP) carboxykinase that catalyzes the first committed step in gluconeogenesis, replenishing PEP from oxaloacetate (Mukhopadhyay *et al*, 2001). PckA links the TCA cycle to synthesis of hexose phosphates and is required to channel carbon from fatty acids and other non‑carbohydrate substrates into gluconeogenic intermediates and ultimately cell‑wall precursors and other cellular components. | Xanthine based human PEPCK inhibitors have been described (Foley *et al*, 2003a, b). However, selective inhibitors of *Mtb* PckA have not been reported. The crystal structure of *Mtb* PckA and its biochemical mechanism are available (Machova *et al*, 2017; Machova *et al*, 2015). | *PckA* is dispensable for growth of *Mtb* in standard media but required for growth on gluconeogenic carbon sources. PckA knockouts are unable to establish infections in mice and conditional depletion of PEPCK prevents survival of *Mtb* in chronic infections (Marrero *et al*, 2010). Deletion of *pckA* also reduces growth at low pH (Dechow & Abramovitch, 2024). |
| LpdC | LpdC acts as the E3 component to catalyze NAD(H) dependent redox cycling of the lipoamide prosthetic group attached to the E2 components of pyruvate dehydrogenase, α-ketoglutarate dehydrogenase and branched chain ketoacid dehydrogenase; and as E1 for a peroxynitrite reductase-peroxidase with AhpD and AhpC. | Co-crystal resolved, species-selective indazole (Ginn *et al*, 2021) and 2-cyanoindole sulfonamide (Sun *et al*, 2024) inhibitors are competitive with lipoamide and growth inhibitory to *Mtb* in carbohydrate-based media and bactericidal for *Mtb* in axenic culture under NO stress and in *Mtb*-infected primary mouse macrophages. Resistance to both scaffolds is generated via SNPs in *lpdC* (Sun et al., 2024). On-target activity is further supported by intra-*Mtb* accumulation of pyruvate and other ketoacids upon exposure (Ginn *et al.*, 2021). 2-cyanoindole analogs demonstrate efficacy in acute TB infection in mice (R. Bryk et al., unpublished data). | *Mtb* with a *lpdC* gene deletion cannot grow in carbohydrate-based media, is highly susceptible to NO stress, and could not establish infection in mice (Venugopal *et al*, 2011). |
| GlcB | GlcB (malate synthase) is the second enzyme of the glyoxylate shunt, which converts glyoxylate into malate by incorporating acetyl from acetyl-CoA. It is essential for utilization of fatty acid- derived carbon source and for the detoxification of glyoxylate. | Phenyl-diketo acid (PDKA) inhibitors of malate synthase (GlcB) inhibit growth of *Mtb* on acetate and reduce growth of *Mtb* in mice. Crystal structures of GlcB in complex with PDKA inhibitors have been solved and overexpression of GlcB reduces the MIC of these inhibitors (Krieger *et al*, 2012). | *GlcB* has been classified as an *in vitro* essential *Mtb* gene by TnSeq and CRISPRi (Bosch *et al.*, 2021; DeJesus *et al.*, 2017). Induced depletion of GlcB prevents *Mtb* from establishing the infection in the mouse model of TB and later induction at any stage of infection results in bacteria clearing up (Puckett *et al*, 2017). |
| Mdh | Mdh (malate dehydrogenase) catalyzes the final step of the Krebs cycle: the reversible conversion of L-malate ((S)-malate) to oxaloacetate, coupled to the conversion of NAD^+^ to NADH + H^+^. While highly endergonic, this reaction is driven in the forward direction by coupling to the downstream condensation of its product, oxaloacetate with acetyl CoA to generate citrate. This reaction is also catalyzed by Rv2852c, an annotated malate quinone oxidoreductase, that is also essential for optimal growth in 7H9 (Harold *et al*, 2022). | A high throughput screen of phenotypically active compounds against *Mtb* in 7H9 identified a compound that inhibited purified recombinant *Mtb* MDH *in vitro* and enhanced phenotypic potency against a strain of *Mtb* in which levels of MDH were reduced. A crystal structure of *Mtb* MDH revealed a species-specific loop overlying the NAD binding pocket (Rittershaus *et al*, 2018). | *Mdh* is essential for growth in standard 7H9, survival in stationary phase, survival in hypoxia, and survival in C57BL/6 mice (Rittershaus *et al.*, 2018). |
| Cell Division | | | |
| FtsZ | FtsZ is critical for cell division and polymerizes to form the Z-ring, which serves as a scaffold for recruitment of other cell division proteins (Hong *et al*, 2013). | Trisubstituted benzimidazoles series have been proposed as FtsZ inhibitors (Awasthi *et al*, 2013; Haranahalli *et al*, 2021; Kumar *et al*, 2011; Lin *et al*, 2018), but in several cases evidence that FtsZ is indeed the potency-determining target in *Mtb* remains incomplete. Nonetheless, several molecules in this class are potent *in vitro* and have *in vivo* activity in the mouse model (Knudson *et al*, 2015; Knudson *et al*, 2014).  Compounds that disrupt the interaction of FtsZ and SepF also inhibit growth and cause filamentation (Huang *et al*, 2007; Zhang *et al*, 2023a).  TBE-12 ((9,10-dioxo-9,10-dihydroanthracen-2-yl) methyl) triphenylphosphonium bromide) inhibits FtsZ GTPase activity in *Msm* and overexpression of an FtsZ-GFP fusion increases the MIC of TBE-12 (Lin *et al.*, 2018).  Additional molecules (pyridopyrazine and pyrimidothiazine analogs) were identified by virtual screening but have not been confirmed to have whole cell activity (Suresh *et al*, 2023). | *FtsZ* is essential for growth of *Mtb* *in vitro* and among the genes most vulnerable to partial genetic inactivation (Bosch *et al.*, 2021; DeJesus *et al.*, 2017). Silencing of *ftsZ* in *Msm* disrupts cell division and leads to filamentation (Blokpoel *et al*, 2005; Ehrt *et al*, 2005). |
| PknA, PknB | PknA and PknB are essential eukaryotic-like Ser/Thr kinases that act together as a central signaling hub controlling cell wall synthesis, cell division, and growth of *Mtb*. | Several small-molecules that are either dual inhibitors of PknA/B or primarily inactivate PknB have been described in literature (Fernandez *et al*, 2006; Lougheed *et al*, 2011; Mori *et al*, 2019; Thongdee *et al*, 2022; Vieira *et al*, 2021; Wang *et al*, 2017a). Many of these compounds are either not active against *Mtb* or evidence that growth inhibition is due to engagement of PknB is either incomplete or missing entirely.  However, for the 5-substituted pyrimidines, which are potent inhibitors of PknB (Ki = 0.004 uM) and PknA (Ki = 0.018), inhibition of *Mtb* growth via engagement of PknA/B is supported by chemical-genetic and phosphoproteomic data (Carette *et al*, 2018).  For indolocarbazoles and staurosporine, overexpression of PknB increased the MIC (Fernandez *et al.*, 2006; Mori *et al.*, 2019). IMB-YH-8 produces cell wall/morphological and transcriptional changes consistent with PknB pathway perturbation (Xu *et al*, 2017a); imidazopyridine aminofurazans reduce PknB‑mediated GarA phosphorylation and are susceptible to mutations in *pknB* (Wlodarchak *et al*, 2018). | *PknA and pknB* are essential for growth of *Mtb* *in vitro* and in mice (Bosch *et al.*, 2021; Chawla *et al*, 2014; DeJesus *et al.*, 2017; Fernandez *et al.*, 2006; Nagarajan *et al*, 2015). |
| MtrA | MtrA encodes the response regulator of the MtrAB-LpqB system. MtrA is activated via phosphorylation by the sensor kinase MtrB, which responds to yet-undefined signals. Once phosphorylated, MtrA binds to DNA and regulates transcription of target genes involved in cell envelope remodeling, cell division, and DNA replication. | Small molecules that effectively engage MtrA in *Mtb* remain to be developed. | Genetic inactivation of *mtrA* is growth inhibitory *in vitro* and bactericidal in *Mtb*-infected macrophages (Li *et al*, 2022). Partial silencing of *mtrA* sensitizes *Mtb* to multiple antitubercular compounds, including bedaquiline, clarithromycin, rifampicin and vancomycin (Li *et al.*, 2022). |
| ***cAMP Metabolism*** | | | |
| CyA | CyA is a membrane-associated class III adenylyl cyclase catalyzes the conversion of ATP to cyclic AMP (cAMP), a second messenger involved in regulating stress adaptation, lipid metabolism, and virulence in *Mtb*. Elevated cAMP levels inhibit the ability of *Mtb* to utilize cholesterol, a critical pathway for persistence during infection, and rewire transcriptional networks linked to virulence and host adaptation. | Small-molecule agonists such as V-59, mCLB073 (also known as TBD11), and GSK2556286 (referred to as GSK286) directly activate CyA. These compounds raise intracellular levels of cyclic AMP (cAMP) by 50- to 140-fold and inhibit the growth of *Mtb* on cholesterol-rich substrates. These compounds mimic the effects of CyA overexpression, lead to the downregulation of cholesterol catabolic genes, and exhibit bacterial growth inhibition that depends on CyA.  Medicinal optimization of V‑59 yielded mCLB073, which entered Phase 1 clinical testing in 2024, and GSK2556286 has shown potential to shorten treatment duration in preclinical models of the Nix-TB regimen.  Loss‑of‑function mutations in *cya* confer resistance to all known agonists and abolish compound‑induced cAMP accumulation (Brown *et al*, 2023; Martinez *et al*, 2025; Wilburn *et al*, 2022). | *Cya* is dispensable for growth of *Mtb* in standard media (Bosch *et al.*, 2021; DeJesus *et al.*, 2017). Overexpression of CyA increases drug sensitivity, while genetic disruption reduces intracellular survival and virulence, underscoring the enzyme's importance in host adaptation (Brown *et al.*, 2023; Wilburn *et al.*, 2022). |
| ***Arabinogalactan Biosynthesis*** | | | |
| DprE1 | DprE1 (decaprenylphosphoryl-β-D-ribose 2′-epimerase 1) is a flavoenzyme that catalyzes a key step in the biosynthesis of the cell wall. It converts decaprenylphosphoryl-β-D-ribose (DPR) into decaprenylphosphoryl-D-arabinose (DPA), a precursor required for the production of arabinogalactan and lipoarabinomannan, which are essential components of the mycobacterial cell envelope (Mikusova *et al*, 2005; Wolucka, 2008). | DprE1 is the target of several small-molecule inhibitors that are active against *Mtb* in mice and for which resistance mutations map to *dprE1*, including (i) benzothiazinones (BTZ043, PBTZ169/Macozinone) (Makarov *et al*, 2009; Neres *et al*, 2012); (ii) dinitrobenzamides and related benzoxacinones (Riccardi *et al*, 2013; Richter *et al*, 2018); (iii) Quabodepistat (OPC-167832) (Hariguchi *et al*, 2020); and (iv) 1,4-azaindole derivatives, such as TBA-7371 (Chatterji *et al*, 2014; Shirude *et al*, 2013; Shirude *et al*, 2014).  Quabodepistat, TBA-7371, and Macozinone are in clinical development. | *DprE1* has been classified as essential by TnSeq and CRISPRi (Bosch *et al.*, 2021; DeJesus *et al.*, 2017). Conditional silencing of *dprE1* results in swelling of the bacteria, cell envelope damage and lysis (Kolly *et al*, 2014) and genetic depletion of DprE1 attenuates *Mtb* in mice (Boldrin *et al*, 2018). |
| EmbC, EmbA, EmbB | EmbC, EmbA and EmbB are membrane-embedded arabinosyltransferases (AraTs) required for the synthesis of arabinogalactan (AG) and lipoarabinomannan (LAM). EmbB and EmbA form a heteromeric complex that catalyzes branching reactions. EmbC contributes to arabinan chain elongation (Berg *et al*, 2007; Escuyer *et al*, 2001; Zhang *et al*, 2020). | EmbC and EmbB are the direct targets of ethambutol (EMB), a first-line drug for treatment of TB. EMB inhibits the biochemical activity of these arabinosyltransferases and Cryo-EM maps demonstrated binding of EMB to the active-site regions of EmbB and EmbC (Zhang *et al.*, 2020). Mutations in *embB* are associated with EMB-resistant clinical *Mtb* isolates (Telenti *et al*, 1997) and many of these map near the EMB binding site (Zhang *et al.*, 2020), the arabinose donor decaprenyl-phosphoryl-D-arabinose (DPA) accumulates in EMB-treated *Msm* (Wolucka et al, 1994). Overexpression of EmbC decreases growth inhibition by EMB; EmbC underexpression increases it (Korkegian *et al*, 2014). | *EmbCAB* are essential for growth of *Mtb* (Amin *et al*, 2008; Bosch *et al.*, 2021; DeJesus *et al.*, 2017) |
| UbiA | UbiA catalyzes the transfer of a pentosylribose moiety from phosphoribosyl pyrophosphate to decaprenyl phosphate, channeling decaprenyl phosphate into decaprenylphospho-D-arabinofuranose, a key intermediate in the arabinogalactan biosynthesis. It serves as the sole donor of arabinose in *Mtb*. | An *ubiA* conditional knockdown *Mtb* strain was hypersusceptible to a compound called KRT2029 (Kolly *et al.*, 2014) but target specificity has not been established. | Genetic inactivation of *ubiA* leads to bacillary swelling, cell wall damage, lysis and death *in vitro* (Kolly *et al.*, 2014) and *ubiA* is among the genes most vulnerable to partial genetic inactivation (Bosch *et al.*, 2021). |
| ***Lipid Biosynthesis*** | | | |
| PptT | PptT catalyzes covalent transfer of a phosphopantetheine moiety from coenzyme A to a conserved serine residue of multiple acyl carrier domain proteins that are involved in the biosynthesis of structural and virulence-associated lipids in *Mtb*. | Amidinoureas were the first whole-cell active inhibitors identified with validated on-target activity, as demonstrated through hypomorphic and hypermorphic strain profiling, resistance mutations in pathway and target genes and solutions of co-crystal structures. Although amidinoureas function as partial inhibitors, compound 8918 prevented *Mtb* growth in mice, with a 100 mg/kg dose producing an effect comparable to that of 10 mg/kg rifampicin (Ballinger *et al*, 2019; Ottavi *et al*, 2023; Ottavi *et al*, 2022). Raltitrexed and thioquinazolinones have also been validated for on-target activity—Raltitrexed through enzyme-bound structural analysis (Singh *et al*, 2024) and thioquinazolinones through resistant mutant and hypomorphic strain profiling (Singh *et al*, 2025). | Genetic inactivation of *pptT* was bactericidal, inhibiting bacterial growth *in vitro* as well as during both the acute and chronic phases of infection in mice (Leblanc *et al*, 2012). |
| ***Mycolic Acid Biosynthesis*** | | | |
| KasA | KasA is the β-ketoacyl synthase component of the type II fatty acid synthase (FAS-II) system. It catalyzes the addition of 2-carbon units to acyl-AcpM in each round of the Fas-II cycle to generate the long chain fatty acids that are required for the synthesis of mycolic acids. | Multipe KasA inhibitor classes with potent *in vitro* activity against *Mtb* have been identified and were reviewed recently (Rudraraju et al, 2022)*.*  The natural product thiolactomycin (TLM) inhibits KasA, KasB and FabH and is active against extracellular and intracellular mycobacteria. Inhibition of cell-wall synthesis by TLM has been demonstrated in *Msm* and *M. bovis* BCG (Kremer *et al*, 2000; Slayden *et al*, 1996).  Indazole sulphonamides have been extensively explored by the Freundlich and GSK groups, showing activity *in vitro* and in mice (Abrahams *et al*, 2016). The original GSK series was abandoned due to genotoxicity, but other groups have made progress in maintaining the antitubercular activity while reducing mutagenicity (Warapande *et al*, 2025). On-target activity of this chemotype is supported by isolation of resistance mutation in *kasA* and *in vitro* inhibition of KasA enzyme (Cunningham *et al*, 2020). KasA inhibitors also combine favorably with bedaquiline‑ and pretomanid‑containing regimens and may contribute to treatment shortening (Soni *et al*, 2025).  Cyclic sulfamates repersent a newer series that act via inhibition of KasA (Bartolomeu Halicki *et al*, 2025). | KasA is essential for *in vitro* growth under standard aerobic, replicating conditions as demonstrated by numerous transposon library approaches, including by DeJesus *et al.* (DeJesus *et al.*, 2017). Genetic knockdown confirmed that KasA is also a vulnerable target (Bosch *et al.*, 2021). |
| InhA | InhA is the NADH-dependent enoyl-ACP reductase of the type II fatty acid synthase (FAS-II) system. It reduces the trans C2–C3 double bond of long-chain acyl-ACP thioesters to generate the long fatty acyl precursors required for mycolic acid biosynthesis (Quemard *et al*, 1995). | Activated isoniazid (INH) forms an isonicotinyl–NAD(H) adduct that binds InhA with sub‑nanomolar affinity; crystal structures of ternary InhA–NAD(H)–INH complexes visualize this adduct in the active site (Rozwarski *et al*, 1998). On-target activity against *Mtb* is supported by (i) INH resistance mutations in *inhA*; (ii) the decrease in potency caused by overexpression of InhA confers resistance to INH; (iii) the decrease in potency caused by InhA substitutions (e.g., S94A) that alter NADH binding (Banerjee *et al*, 1994; Lempens *et al*, 2018); (iv) the depletion of mycolic acids that occurs after exposing *Mtb* to INH; (v) the cell‑wall morphological changes cause by exposing *Mtb* to INH; and (vi) that genetic inactivation of *inhA* phenocopies these effects (Schroeder *et al*, 2002).  InhA is also inhibited by ethionamide, pyridomycin, 4‑hydroxy‑2‑pyridones, and triclosan (Hartkoorn *et al*, 2012; Manjunatha *et al*, 2015; North *et al*, 2014) | *InhA* has been classified as an *in vitro* essential *Mtb* gene by TnSeq (DeJesus *et al.*, 2017) and is among the genes most vulnerable to partial genetic inactivation (Bosch *et al.*, 2021). |
| HadA, HadB, HadC | Heterodimers of HadAB and HadBC form the (*3R)*-hydroxyacyl-ACP dehydratases of the FAS-II system. These catalyze the dehydration of (3*R*)-hydroxyacyl-ACP during the FAS-II elongation cycles, with HadB serving as the catalytic subunit of both heterodimers. HadAB is most likely involved in the early fatty acid elongation cycles, producing intermediate‑size meromycolic chains, whereas HadBC is implicated in late‑stage meromycolic chain biosynthesis with a preference for longer substrates (Sacco *et al*, 2007). | The prodrugs isoxyl (ISO, MIC 1-10 µg/ml) and thiacetazone (TAC, MIC 0.1-0.5 µg/ml), both once used to treat TB, inhibit HadAB by covalently modifying Cys61 of HadA (Grzegorzewicz *et al*, 2015). On-target activity is supported by (i) the accumulation of 3-hydroxy C18, C20, and C22 fatty acids in response to exposing *Mtb* to ISO or TAC; and (ii) resistance mutations *hadA*.  A series of 1,3-diarylpyrazolyl-acylsulfonamides also inhibits HadAB and disrupts mycolic acid biosynthesis in *Mtb* (Singh et al, 2022). Inhibition is mediated by non-covalent interactions within the HadAB active site. | The *hadA-hadC* gene cluster was first shown to be essential *in vitro* via a homologous recombination study (Sacco *et al.*, 2007), and later confirmed via genome wide TnSeq (DeJesus *et al.*, 2017) and CRISPRi studies (Bosch *et al.*, 2021). |
| FadD32 | FadD32 is required for mycolic acid biosynthesis. It catalyzes the activation of long-chain fatty acids via their adenylation to acyl-adenylates (acyl-AMP). The activated fatty acids are subsequently transferred to polyketide synthase (Pks13) for further chain extension (Gavalda *et al*, 2009; Leger *et al*, 2009; Trivedi *et al*, 2004). | Radiolabeling experiments demonstrated that the fatty acyl ACP synthetase activity of FadD32 is inhibited by 4,6-diaryl-5,7-dimethyl coumarins (Stanley *et al*, 2013). Several compounds from this series are potent against *Mtb* (0.42 - 0.24 µM) and were active against *M. marinum* in the zebrafish model. On-target validation evidence includes (i) resistance mutations mapping to *fadD32*; (ii) episomal expression of mutant FadD32 alleles (E120A or F291L) confers dominant resistance in wild-type strains; and (iii) failure to inhibit acyl-AcpM adduct formation in a resistant mutant.  Scaffold morphing from the coumarins led to a quinoline-2-carboxamides series with maintained FadD32 activity, improved ADME/PK properties and *in vivo* efficacy an 8-day acute mouse model (Fang *et al*, 2018). On-target evidence for this series rests primarily on cross-resistance of mutants isolated for quinoline analogs.  More recently, the 2,4-dibromo-6-[3-(trifluoromethyl)-1,2-oxazol-5-yl]phenol (M1) was identified via virtual screening of an isoxazole scaffold library against FadD32 (Rani *et al*, 2025). M1 activity was confirmed against FadD32 enzymatically and showed ex vivo and *in vivo* activity against *Mtb*. Transcriptomic analysis of *Mtb* exposed to M1 reveals down‑regulation and dysregulation of genes involved in cell‑wall and mycolic‑acid biosynthesis, supporting on‑target interference with FadD‑dependent pathways. | Disruption of *fadD32* resulted in mycolic acid deficiency in *C. glutamicum* and was shown to be essential for growth of *M. smegmatis* *in vitro* (Portevin *et al*, 2005). Essentiality of *fadD32* in *Mtb* was confirmed by TnSeq (DeJesus *et al.*, 2017) and CRISPRi (Bosch *et al.*, 2021). |
| Pks13 | Pks13 catalyzes the final condensation step in mycolic acid biosynthesis, joining meromycolyl and α-chain precursors to produce the long-chain mycolic acids that form the *Mtb* cell wall. These lipids provide structural rigidity and impermeability, protecting the bacterium from antibiotics and host immune system. Structural studies on the enzyme corroborate its central role in mycolic-acid assembly (Kim et al., 2023). | Multiple chemotypes targeting four of the five Pks13 domains (N-acyl carrier protein domain (Wilson *et al*, 2013), N-acyl carrier protein / ketosynthase domain (Green *et al*, 2025), acyltransferase (AT) domain (Krieger *et al*, 2025), and thioesterase (TE) domain (Green *et al*, 2023; Krieger *et al*, 2024; Wilson *et al*, 2022)), establishing this enzyme as a druggable target.  Early thiophene derivatives inhibited bacterial growth by covalently modifying the AT domain, directly linking disruption of mycolic acid biosynthesis to *pks13* mutations.  The benzofuran amide, TAM16, was later identified as a selective inhibitor of the TE domain, blocking product release and enhancing susceptibility to front-line agents.  Optimization of coumestan analogs improved metabolic stability and minimized off-target ion channel activity while maintaining sub-μM potency.  The most advanced chemotype, CMX410, is a covalent SuFEx-based inhibitor that acylates the catalytic Ser801 in the AT domain, irreversibly blocking acyl chain transfer. CMX410 displays low-nanomolar potency, activity against drug-sensitive and MDR/XDR *Mtb* strains, and robust efficacy in murine TB infection models. Mutations conferring resistance to the SuFEx-based inhibitors map to *pks13* and overexpression of Pks13 increases the MIC of these compounds (Khola *et al*, 2024; Krieger *et al.*, 2025; Krieger *et al.*, 2024; Wilson *et al.*, 2013) | Genome-scale saturated transposon mutagenesis identified *pks13* as essential for the growth of *Mtb* *in vitro* (DeJesus *et al.*, 2017) and CRISPRi demonstrated *Mtb*’s vulnerability to partial genetic inactivation of Pks13 (Bosch *et al.*, 2021). Depletion of Pks13 in *Msm* blocks terminal mycolate condensation, leading to rapid loss of viability (Portevin *et al*, 2004). |
| MmpL3 | MmpL3 is required to synthesize the mycomembrane of mycobacteria, using proton translocation as the driving force to transport trehalose monomycolates (TMM) across the cytosolic membrane. MmpL3 is unique to mycobacteria (Stevens *et al*, 2022; Xu *et al*, 2017b). | Numerous scaffolds have been identified which inhibit MmpL3 function including adamantyl ureas, aryl indoles, pyrazoles, oxazoles, thiazoles, and indolecarboxamides (Baldin *et al*, 2025; Bolla, 2020; Grzegorzewicz *et al*, 2012; Li *et al*, 2016; Li *et al*, 2014; Shao *et al*, 2020; Williams *et al*, 2019; Zhao *et al*, 2022). Inhibition of MmpL3 is bactericidal *in vitro* and compounds have activity against intracellular and extracellular activity. Several classes have been confirmed to have *in vivo* activity in the TB mouse model, most notably the indolecarboxamides (Stec *et al*, 2016).  On-target activity has been confirmed for many compounds by (i) isolation of resistant mutants, (ii) activity against hyper-hypomorphs, and (ii) intracellular accumulation of mycolates (Grover *et al*, 2021; Li *et al.*, 2014; McNeil *et al*, 2020). | *MmpL3* is essential for growth in standard medium, macrophages and in the mouse model of infection (Cheung *et al*, 2022; Degiacomi *et al*, 2017; Li *et al.*, 2016; McNeil & Cook, 2019; Poce *et al*, 2013; Williams *et al*, 2024). |
| ***Respiratory Chain*** | | | |
| Ndh, NdhA | Type 2 NADH dehydrogenases (Ndh, NdhA) catalyze electron transfer from NADH to the quinone pool and regenerate NAD+ from NADH. | Several inhibitors have been identified and were reviewed recently (Wu *et al*, 2025). Activity of 2-mercaptoquinazolinones and tricyclic spironolactones against *Mtb* is restricted to media containing fatty acids, which is consistent with on-target whole-cell activity (Beites *et al*, 2019). Efficient killing of *Mtb* by inhibition of Ndh requires simultaneous inactivation of type I NADH dehydrogenase (Beites *et al.*, 2019; Xu *et al*, 2023). | Together, type 2 NADH dehydrogenases are essential for growth in fatty-acid containing media but dispensable for growth in fatty-acid free media. They are required for optimal growth of *Mtb* in mice but inactivation on Ndh and NdhA is insufficient to prevent growth entirely (Beites *et al.*, 2019). |
| Cytochrome *bc*_1_-*aa*_3_ oxidase | The cytochrome *bc*_1-_*aa*_3_ oxidase supercomplex transfers electrons from menaquionol to oxygen, reducing it to water and contributing to the proton motive force (PMF) that drives ATP synthesis. | Several inhibitors have been identified and were recently reviewed (Bajeli *et al*, 2020)).  On-target whole-cell activity for several inhibitors is supported (i) by resistance mutations (Pethe *et al*, 2013), (ii) failure to inhibit *Mtb* in which cytochrome *bc*_1_-*aa*_3_ oxidase has been genetically inactivated (Beites *et al.*, 2019), (iii) and synergy with inhibition of cytochrome *bd* oxidase (Beites *et al.*, 2019).  Telacebec (Q203) has shown variable efficacy in mice - from potent killing of wild‑type *Mtb* (Pethe *et al.*, 2013) to activity restricted to strains lacking cytochrome bd oxidase (Kalia *et al*, 2017) – but demonstrates activity in a phase 2 clinical trial (de Jager *et al*, 2020; Janssen *et al*, 2025).  Recent work indicates that cytochrome *bc1*-*aa3*, including telacebec, can provide sterilizing and treatment‑shortening benefits in clinically relevant regimens based on mouse relapse studies, and that clinical *Mtb* isolates are more susceptible to cytochrome *bc1*-*aa3* inhibition (Aguilar-Perez *et al*, 2025). | Complete genetic inactivation reduces growth *in vitro* and in mice. Simultaneous inactivation of cytochrome *bc*_1_-*aa*_3_ oxidase and cytochrome *bd* oxidase prevents growth *in vitro* and kills *Mtb* in mice (Beites *et al.*, 2019). |
| Cytochrome *bd* oxidase (CydA, CydB) | Cytochrome *bd* oxidase is an alternative respiratory terminal oxidase that transfers electrons from menaquinol to oxygen but does not contribute to the generation of PMF. | Inhibitors of cytochrome bd oxidase have little activity by themselves, but show in vitro and in vivo efficacy in combination with telacebec, with a significant though modest effect size in mice (Jeffreys *et al*, 2023; Lee *et al*, 2021).  On-target whole-cell activity is supported by (i) the requirement for telacebec to be effective, (ii) RNAseq experiments demonstrating that the combination of ND-011992 with telacebec affects the *Mtb* transcriptome in a manner that is similar to the impact telacebec has on the transcriptome of *Mtb* lacking cytochrome *bd* oxidase, and (iii) that impact on oxygen consumption rate is reduced by overexpression of cytochrome *bd* oxidase (Jeffreys *et al.*, 2023; Lee *et al.*, 2021). | Deletion of *cydAB* does not impact growth of *Mtb* in standard media but increases susceptibility of *Mtb* to killing by an activated immune system and to acid stress (Beites *et al.*, 2019; Cai *et al*, 2021). Simultaneous inactivation of cytochrome *bc*_1_-*aa*_3_ oxidase and cytochrome *bd* oxidase prevents growth *in vitro* and kills *Mtb* in mice (Beites *et al.*, 2019). |
| ATP synthase | ATP synthase catalyzes the synthesis of ATP from ADP and inorganic phosphate, using the PMF generated by respiration. | Bedaquiline (BDQ), the best‑characterized ATP synthase inhibitor, is active against *Mtb* in vitro and in mouse models and is a cornerstone of regimens that shorten treatment for drug‑resistant TB (Andries *et al*, 2005; Conradie *et al.*, 2020).  Evidence for on-target whole cell activity includes (i) resistance mutations in *atpE* (which encodes the c subunit of ATP synthase), and (ii) depletion of ATP pools, which is prevented by BDQ resistance mutations (Andries *et al.*, 2005; Koul *et al*, 2014).  Squaramides, e.g., SQ31f, are another class of ATP synthase inhibitors (Tantry *et al*, 2017). SQ31f and BDQ bind overlapping regions of the Fo subunit but occupy distinct pockets at different depths and with different stoichiometries (Courbon *et al*, 2023). On‑target activity is supported by resistance mutations, which partly overlap with those conferring resistance to BDQ (e.g. *atpE* D28), but many bedaquiline atpE mutants (A63/I66) remain squaramide‑susceptible. | ATP synthase is essential for growth of *Mtb* under all conditions tested and vulnerable to partial genetic inactivation (Bosch *et al.*, 2021; DeJesus *et al.*, 2017). |
| MenG | MenG is a SAM-dependent methyltransferase that catalyzes the terminal methylation of demethylmenaquinone (DMK-9) to menaquinone-9 (MK-9), a key redox cofactor in the *Mtb* respiratory chain. Menaquinone is essential for ATP synthesis and redox balance under both replicating and non-replicating conditions (Johnston *et al*, 2003). | Small-molecule inhibitors of MenG, including DG70 and later analogs, block the conversion of DMK-9 to MK-9, collapse the proton motive force, and are bactericidal to both actively growing and nutrient-starved persisters (Sukheja *et al*, 2017). Optimization has produced next-generation scaffolds such as benzothiazole, benzoxazole, and aminobenzimidazole derivatives with improved potency and physicochemical properties (Sharma *et al*, 2025; Velappan *et al*, 2020).  Inhibitors derived from GSK517A inhibit MenG with low-nanomolar biochemical potency (JNJ-6887 IC50 ~1.1–1.3 nM; JNJ-1866 12 ± 2.8 nM)(Wetzel *et al*, 2026). JNJ-6887 kills *Mtb*, reduces respiration, and synergizes with bedaquiline to resensitize Rv0678 efflux-mediated bedaquiline-resistant *Mtb*. On-target whole-cell activity is supported by (i) resistance selections mapping to *menG*, (ii) MenG-directed CRISPRi hypersensitization, and (iii) menaquinone depletion after compound treatment. | *MenG* has been classified as an *in vitro* essential *Mtb* gene by TnSeq and CRISPRi (Bosch *et al.*, 2021; DeJesus *et al.*, 2017). Loss or inhibition of MenG activity leads to depletion of MK-9, accumulation of DMK-9, collapse of the proton motive force, and cell death under both replicating and non-replicating conditions (Sukheja *et al.*, 2017). |
| ***Mycobactin Biosynthesis*** | | | |
| MbtA | MbtA is the adenylating enzyme that catalyzes the first committed step of mycobactin siderophore biosynthesis. | Salicyl-AMS and its derivatives are the best-studied *Mtb*A inhibitors and show activity both biochemically and against *Mtb* *in vitro* and in mice (Dawadi *et al*, 2016; Dawadi *et al*, 2015; Engelhart & Aldrich, 2013; Ferreras *et al*, 2005; Krajczyk *et al*, 2016; Lun *et al*, 2013; Nelson *et al*, 2015). Their on-target activity is supported by (i) inhibition of mycobactin biosynthesis in *Mtb*; and (ii) reduced potency in media supplemented with salicylate or high iron (Bythrow *et al*, 2019; Dawadi *et al*, 2018; Ferreras *et al.*, 2005). | *Mtb* relies on mycobactins to scavenge ferric iron (Fe3+) and to support growth in iron-limited media (De Voss *et al*, 2000). However, the role of siderophore-dependent iron acquisition varies with host genotype (Smith *et al*, 2022; Tufariello *et al*, 2016) and the specific impact of genetically inactivating MbtA on *Mtb* growth and persistence during infection remains largely unexplored. |
| ***Cofactor Metabolism*** | | | |
| **Coenzyme A (CoA)** | | | |
| CoaBC | CoaBC is a bifunctional enzyme in CoA biosynthesis; its CoaB domain (4‑phosphopantothenoyl‑L‑cysteine synthetase) catalyzes condensation of 4‑phosphopantothenate with cysteine to form 4‑phosphopantothenoyl‑L‑cysteine. | A CoaB inhibitor (4-(5-chloro-1,3-benzoxazol-2-yl)sulfanyl-5-methyl-2-phenyl-pyrazol-3-ol) was identified from a biochemical screen. Evidence for on-target activity in *Mtb* include (i) a metabolomic profile consistent with perturbations in pantothenate and CoA biosynthesis, and (ii) metabolite rescue (CoaBC bypass by pantethine) (Evans *et al*, 2021).  Inhibitors with weak albeit on-target activity, which engage an allosteric site in CoaB, have also been described (Mendes *et al*, 2021). | *CoaBC* has been genetically validated as an essential target *in vitro* (Bosch *et al.*, 2021; DeJesus *et al.*, 2017) and *coaBC* is required for *Mtb* to establish an infection and to grow and persist in mice (Evans *et al*, 2016) |
| PanK | PanK (also referred to as CoaA) is the type I Pantothenate kinase that converts pantothenate to 4’-phosphopantothenate in the CoA biosynthesis pathway. | Biaryl acetic acid inhibitors of PanK inhibit growth of *Mtb*, with on-target activity supported by an MIC upshift upon overexpression of PanK (Reddy *et al*, 2014).  Prodrugs activated by EthA inhibit PyrG and PanK. On-target whole-cell activity against *Mtb* rests on the identification of resistance mutations in *panK* and demonstration of an impact of the resistance-associated mutations o PanK/ CoaA biochemical activity (Chiarelli *et al.*, 2018).  Potent ATP competitive triazoles and quinolones further attest to the biochemical tractability of PanK (Butman *et al*, 2020). These compounds inhibit growth of *panK* hypomorphs but are inactive against wild‑type *Mtb*, an observation consistent with low target vulnerability (Evans *et al.*, 2016; Reddy *et al.*, 2014). | *PanK* is essential for growth of *Mtb* *in vitro*, but relatively invulnerable to partial genetic inactivation (Bosch *et al.*, 2021; Evans *et al.*, 2016; Reddy *et al.*, 2014). |
| **NAD** | | | |
| NadD | NadD catalyzes the adenylation of NaMN (β-nicotinate D-ribonucleotide) to form NaAD (deamido-NAD+), a substrate of NadE, and is essential for the synthesis of NAD(H) and NADP(H). | Screening of a small library of whole cell actives (Osterman *et al*, 2019) yielded weak inhibitors that lack evidence of on-target engagement in *Mtb*. Hits from phenotypic and target-based HTS campaigns in the TBDA are under evaluation. | *NadD* is one of the most vulnerable genes in *Mtb* (Bosch *et al.*, 2021). Inducible degradation of NadD caused a strong bactericidal effect in replicating and nonreplicating *Msm* (Rodionova *et al*, 2014). |
| NadE | Catalyzes the conversion of nicotinic acid adenine dinucleotide (NaAD) to nicotinamide adenine dinucleotide (NAD+), using glutamine as ammonia donor although ammonia can substitute for glutamine *in vitro*. | Inhibitors of NadE that result in intracellular depletion of NAD(H) are cidal to non-replicating as well as replicating *Mtb* *in vitro* with the MBC and MIC values being similar for the most potent compounds (Boshoff *et al*, 2008). Compounds with different pharmacophores have been tested but showed limited improvement in potency (Wang *et al*, 2017b). Clear evidence that potency of these compounds against *Mtb* is primarily due to inhibition of NadE is missing. | Knockdown of NadE is cidal under non-replicating and replicating conditions *in vitro* (Bosch *et al.*, 2021; Kim *et al*, 2013; Rodionova *et al.*, 2014; Sharma *et al*, 2023). Depletion of NadE results in rapid loss of viability in acute and chronic models of mouse infection (Kim *et al.*, 2013). |
| **Chorismate** | | | |
| AroG | AroG (3‑deoxy‑D‑arabino‑heptulosonate‑7‑phosphate synthase, DAHP synthase) catalyzes the first committed step in chorismate biosynthesis, converting erythrose‑4‑phosphate and phosphoenolpyruvate into DAHP. Chorismate is a central branchpoint precursor for aromatic amino acids, folate, and ubiquinone. | No small‑molecule AroG inhibitors with confirmed whole‑cell activity against *Mtb* have been reported to date. | The Aro pathway is essential *in vitro* in standard medium (Parish & Stoker, 2002). AroG is essential in standard medium and in macrophages (Galina *et al*, 2022). Supplementation with L-tryptophan, L-tyrosine, L-phenylalanine, *p*-hydroxybenzoate, *p*-aminobenzoic acid and 2,3-dihydroxybenzoate restores growth only partially.  AroG is the most vulnerable target of the pathway according to genetic screens (Bosch *et al.*, 2021). |
| **Biotin** | | | |
| BioA | BioA (7,8-diaminopelargonic acid synthase) catalyzes the antepenultimate step in the biotin biosynthesis pathway, transferring an amino group from S-adenosyl-L-methionine (SAM) to 7-keto-8-aminopelargonic acid (KAPA) to form 7,8-diaminopelargonic acid (DAPA). | BioA is the target of the natural product amiclenomycin (Mann *et al*, 2005) and can by inhibited by various small molecules, including aryl hydrazines, hydrazides, and pyridoxal 5′‑phosphate–dependent transaminase inhibitors (Dai *et al*, 2014; Liu *et al*, 2017; Liu *et al*, 2025; Park *et al*, 2015).  Recent structure‑guided development has yielded a potent BioA inhibitor that validates biotin synthesis inhibition as an antitubercular strategy, with one compound demonstrating attenuation of *Mtb* in a low‑biotin mouse model of infection (Liu *et al.*, 2025).  On-target whole-cell activity of several BioA inhibitors is supported by loss of potency in biotin-supplemented media and BioA overexpression. | Genes involved in biotin synthesis, including *bioA* and *bioB*, are dispensable for *Mtb* growth in standard laboratory media, which contain biotin. This dispensability *in vitro* enabled assessment of their roles *in vivo* using TnSeq, which showed that *Mtb* must synthesize biotin to proliferate during infection (Sassetti & Rubin, 2003). Conditional *bioA* knockdown demonstrated that endogenous biotin synthesis is essential for *Mtb* persistence in mice. Notably, attenuation of *Mtb* in this model requires BioA expression to be reduced by more than 96% (Woong Park *et al*, 2011). |
| BioB | BioB (biotin synthase) catalyzes the final step of biotin biosynthesis, converting dethiobiotin (DTB) to biotin. | Acidomycin is a natural product that inhibits growth of *Mtb* via inhibition of BioB (Bockman *et al*, 2019; Qu *et al*, 2024). |  |
| BirA | Biotin protein ligase (BirA or BPL) post-translationally attaches biotin to specific lysine residues on biotin-dependent enzymes such as acyl-CoA carboxylases and pyruvate CoA carboxylase. | *Mtb* BirA can be inhibited with a bisubstrate mimic that also has whole-cell activity. That inhibition of *Mtb* growth is due to engagement of BirA is supported by a decrease of biotinylated proteins and a decrease in potency upon BirA overexpression (Duckworth *et al*, 2011). However, subsequent screens to identify additional BirA inhibitors with progressible leads have not been successful. | Genetic inactivation of BirA prevents growth and persistence of *Mtb* in mice (Tiwari *et al*, 2018) and is often sufficient to eliminate *Mtb* from chronically infected mice (Su *et al*, 2021). |
| **Folate** | | | |
| DfrA | DfrA (dihydrofolate reductase) catalyzes the reduction of dihydrofolate (DHF) coupled to oxidation of NADPH to NADP, and thus supplies one‑carbon units for nucleotide and amino acid biosynthesis. | DfrA is a chemically well validated antibacterial target, including against *Mtb*. Methotrexate esters inhibit *Mtb* DfrA *in vitro* and exhibit on-target activity against *Mtb* as demonstrated by knockdown and overexpression (Nixon *et al*, 2014).  DfrA is also targeted by para-aminosalicylic acid (PAS), a pro-drug which is one of the oldest antitubercular agents, introduced in 1946 for treatment of TB (Minato *et al*, 2015; Zheng *et al*, 2013). However, activated PAS does not seem to be a monospecific DHFR inhibitor but rather a multi-target antifolate (Hajian *et al*, 2019). | DfrA has been classified as *in vitro* essential by transposon mutagenesis and CRISPRi (Bosch *et al.*, 2021; DeJesus *et al.*, 2017). |
| **Riboflavin** | | | |
| RibF | RibF is a bifunctional enzyme that catalyzes the conversion of riboflavin to FMN through action of the riboflavin kinase (RFK) domain, and subsequent conversion of FMN to FAD catalyzed by the FMN adenylyltranferase (FMNAT) domain. The FMNAT domain displays significant structural divergence from human FAD synthase which makes it a more attractive target than the RFK domain which shares sequence and similarity to the human ortholog. | Small molecules that effectively engage RibF in *Mtb* remain to be developed. | *RibF* has been classified as an *in vitro* essential *Mtb* gene by TnSeq and CRISPRi (Bosch et al., 2021; De-Jesus et al, 2017). Genetic inactivation of *ribF* by CRISRPi is bactericidal in *Mtb* under standard *in vitro* culture conditions (Chengalroyen *et al*, 2024). |

# References

Abrahams GL, Kumar A, Savvi S, Hung AW, Wen S, Abell C, Barry CE, 3rd, Sherman DR, Boshoff HI, Mizrahi V (2012) Pathway-selective sensitization of Mycobacterium tuberculosis for target-based whole-cell screening. *Chem Biol* 19: 844-854

Abrahams KA, Chung CW, Ghidelli-Disse S, Rullas J, Rebollo-Lopez MJ, Gurcha SS, Cox JA, Mendoza A, Jimenez-Navarro E, Martinez-Martinez MS *et al* (2016) Identification of KasA as the cellular target of an anti-tubercular scaffold. *Nat Commun* 7: 12581

Abrahams KA, Cox JAG, Futterer K, Rullas J, Ortega-Muro F, Loman NJ, Moynihan PJ, Perez-Herran E, Jimenez E, Esquivias J *et al* (2017) Inhibiting mycobacterial tryptophan synthase by targeting the inter-subunit interface. *Sci Rep* 7: 9430

Aguilar-Perez C, Lenaerts AJ, Villellas C, Guillemont J, Dallow J, Painter H, Ammerman NC, Hassan A, Golovkine G, Brock L *et al* (2025) The role of cytochrome bc(1) inhibitors in future tuberculosis treatment regimens. *Nat Commun* 16: 9344

Ahuja SD, Ashkin D, Avendano M, Banerjee R, Bauer M, Bayona JN, Becerra MC, Benedetti A, Burgos M, Centis R *et al* (2012) Multidrug resistant pulmonary tuberculosis treatment regimens and patient outcomes: an individual patient data meta-analysis of 9,153 patients. *PLoS Med* 9: e1001300

Akopian T, Kandror O, Tsu C, Lai JH, Wu W, Liu Y, Zhao P, Park A, Wolf L, Dick LR *et al* (2015) Cleavage Specificity of Mycobacterium tuberculosis ClpP1P2 Protease and Identification of Novel Peptide Substrates and Boronate Inhibitors with Anti-bacterial Activity. *J Biol Chem* 290: 11008-11020

Aldred KJ, Kerns RJ, Osheroff N (2014) Mechanism of quinolone action and resistance. *Biochemistry* 53: 1565-1574

Amin AG, Goude R, Shi L, Zhang J, Chatterjee D, Parish T (2008) EmbA is an essential arabinosyltransferase in Mycobacterium tuberculosis. *Microbiology (Reading)* 154: 240-248

Andries K, Verhasselt P, Guillemont J, Gohlmann HW, Neefs JM, Winkler H, Van Gestel J, Timmerman P, Zhu M, Lee E *et al* (2005) A diarylquinoline drug active on the ATP synthase of Mycobacterium tuberculosis. *Science (New York, NY)* 307: 223-227

Aono A, Murase Y, Chikamatsu K, Igarashi Y, Shimomura Y, Hosoya M, Osugi A, Morishige Y, Takaki A, Yamada H *et al* (2022) In vitro activity of tedizolid and linezolid against multidrug-resistant Mycobacterium tuberculosis: a comparative study using microdilution broth assay and genomics. *Diagn Microbiol Infect Dis* 103: 115714

Awasthi D, Kumar K, Knudson SE, Slayden RA, Ojima I (2013) SAR studies on trisubstituted benzimidazoles as inhibitors of Mtb FtsZ for the development of novel antitubercular agents. *J Med Chem* 56: 9756-9770

Bajeli S, Baid N, Kaur M, Pawar GP, Chaudhari VD, Kumar A (2020) Terminal Respiratory Oxidases: A Targetables Vulnerability of Mycobacterial Bioenergetics? *Front Cell Infect Microbiol* 10: 589318

Baldin VP, Harding CL, Quach D, Sugie J, Pogliano J, Parish T (2025) Thienopyrimidine amide analogs target MmpL3 in Mycobacterium tuberculosis. *Antimicrob Agents Chemother* 69: e0098025

Ballinger E, Mosior J, Hartman T, Burns-Huang K, Gold B, Morris R, Goullieux L, Blanc I, Vaubourgeix J, Lagrange S *et al* (2019) Opposing reactions in coenzyme A metabolism sensitize Mycobacterium tuberculosis to enzyme inhibition. *Science (New York, NY)* 363

Banerjee A, Dubnau E, Quemard A, Balasubramanian V, Um KS, Wilson T, Collins D, de Lisle G, Jacobs WR, Jr. (1994) inhA, a gene encoding a target for isoniazid and ethionamide in Mycobacterium tuberculosis. *Science (New York, NY)* 263: 227-230

Barbosa MD, Lin S, Markwalder JA, Mills JA, DeVito JA, Teleha CA, Garlapati V, Liu C, Thompson A, Trainor GL *et al* (2002) Regulated expression of the Escherichia coli lepB gene as a tool for cellular testing of antimicrobial compounds that inhibit signal peptidase I in vitro. *Antimicrob Agents Chemother* 46: 3549-3554

Bartolomeu Halicki PC, Kim JH, Griffin AF, Rampon DS, Becker KL, Seeliger JC, Schomaker JM, Rohde KH (2025) Targeting Mycolic Acid Biosynthesis with Cyclic Sulfamates: A New Strategy against Mycobacterium tuberculosis. *ACS Infect Dis* 11: 3071-3084

Basarab GS, Ghorpade S, Gibhard L, Mueller R, Njoroge M, Peton N, Govender P, Massoudi LM, Robertson GT, Lenaerts AJ *et al* (2022) Spiropyrimidinetriones: a Class of DNA Gyrase Inhibitors with Activity against Mycobacterium tuberculosis and without Cross-Resistance to Fluoroquinolones. *Antimicrob Agents Chemother* 66: e0219221

Bauman AA, Sarathy JP, Kaya F, Massoudi LM, Scherman MS, Hastings C, Liu J, Xie M, Brooks EJ, Ramey ME *et al* (2024) Spectinamide MBX-4888A exhibits favorable lesion and tissue distribution and promotes treatment shortening in advanced murine models of tuberculosis. *Antimicrob Agents Chemother* 68: e0071624

Beites T, O'Brien K, Tiwari D, Engelhart CA, Walters S, Andrews J, Yang HJ, Sutphen ML, Weiner DM, Dayao EK *et al* (2019) Plasticity of the Mycobacterium tuberculosis respiratory chain and its impact on tuberculosis drug development. *Nature communications* 10: 4970

Berg S, Kaur D, Jackson M, Brennan PJ (2007) The glycosyltransferases of Mycobacterium tuberculosis - roles in the synthesis of arabinogalactan, lipoarabinomannan, and other glycoconjugates. *Glycobiology* 17: 35-56R

Blanco D, Perez-Herran E, Cacho M, Ballell L, Castro J, Gonzalez Del Rio R, Lavandera JL, Remuinan MJ, Richards C, Rullas J *et al* (2015) Mycobacterium tuberculosis gyrase inhibitors as a new class of antitubercular drugs. *Antimicrob Agents Chemother* 59: 1868-1875

Block AM, Wiegert PC, Namugenyi SB, Tischler AD (2024) Transposon sequencing reveals metabolic pathways essential for Mycobacterium tuberculosis infection. *PLoS Pathog* 20: e1011663

Blokpoel MC, Murphy HN, O'Toole R, Wiles S, Runn ES, Stewart GR, Young DB, Robertson BD (2005) Tetracycline-inducible gene regulation in mycobacteria. *Nucleic Acids Res* 33: e22

Bockman MR, Engelhart CA, Cramer JD, Howe MD, Mishra NK, Zimmerman M, Larson P, Alvarez-Cabrera N, Park SW, Boshoff HIM *et al* (2019) Investigation of ( S)-(-)-Acidomycin: A Selective Antimycobacterial Natural Product That Inhibits Biotin Synthase. *ACS Infect Dis* 5: 598-617

Boldrin F, Degiacomi G, Serafini A, Kolly GS, Ventura M, Sala C, Provvedi R, Palu G, Cole ST, Manganelli R (2018) Promoter mutagenesis for fine-tuning expression of essential genes in Mycobacterium tuberculosis. *Microb Biotechnol* 11: 238-247

Bolla JR (2020) Targeting MmpL3 for anti-tuberculosis drug development. *Biochem Soc Trans* 48: 1463-1472

Bonnett S, Jee JA, Chettiar S, Ovechkina Y, Korkegian A, Greve E, Odingo J, Parish T (2023) Identification of 2-Amino Benzothiazoles with Bactericidal Activity against Mycobacterium tuberculosis. *Microbiol Spectr* 11: e0497422

Bonnett SA, Ollinger J, Chandrasekera S, Florio S, O'Malley T, Files M, Jee JA, Ahn J, Casey A, Ovechkina Y *et al* (2016) A Target-Based Whole Cell Screen Approach To Identify Potential Inhibitors of Mycobacterium tuberculosis Signal Peptidase. *ACS Infect Dis* 2: 893-902

Bosch B, DeJesus MA, Poulton NC, Zhang W, Engelhart CA, Zaveri A, Lavalette S, Ruecker N, Trujillo C, Wallach JB *et al* (2021) Genome-wide gene expression tuning reveals diverse vulnerabilities of M. tuberculosis. *Cell* 184: 4579-4592 e4524

Boshoff HI, Xu X, Tahlan K, Dowd CS, Pethe K, Camacho LR, Park TH, Yun CS, Schnappinger D, Ehrt S *et al* (2008) Biosynthesis and recycling of nicotinamide cofactors in mycobacterium tuberculosis. An essential role for NAD in nonreplicating bacilli. *The Journal of biological chemistry* 283: 19329-19341

Botella L, Vaubourgeix J, Livny J, Schnappinger D (2017) Depleting Mycobacterium tuberculosis of the transcription termination factor Rho causes pervasive transcription and rapid death. *Nature communications* 8: 14731

Brown AC, Eberl M, Crick DC, Jomaa H, Parish T (2010) The nonmevalonate pathway of isoprenoid biosynthesis in Mycobacterium tuberculosis is essential and transcriptionally regulated by Dxs. *J Bacteriol* 192: 2424-2433

Brown KL, Wilburn KM, Montague CR, Grigg JC, Sanz O, Perez-Herran E, Barros D, Ballell L, VanderVen BC, Eltis LD (2023) Cyclic AMP-Mediated Inhibition of Cholesterol Catabolism in Mycobacterium tuberculosis by the Novel Drug Candidate GSK2556286. *Antimicrobial agents and chemotherapy* 67: e0129422

Butman HS, Kotze TJ, Dowd CS, Strauss E (2020) Vitamin in the Crosshairs: Targeting Pantothenate and Coenzyme A Biosynthesis for New Antituberculosis Agents. *Front Cell Infect Microbiol* 10: 605662

Bythrow GV, Mohandas P, Guney T, Standke LC, Germain GA, Lu X, Ji C, Levendosky K, Chavadi SS, Tan DS *et al* (2019) Kinetic Analyses of the Siderophore Biosynthesis Inhibitor Salicyl-AMS and Analogues as MbtA Inhibitors and Antimycobacterial Agents. *Biochemistry* 58: 833-847

Cai Y, Jaecklein E, Mackenzie JS, Papavinasasundaram K, Olive AJ, Chen X, Steyn AJC, Sassetti CM (2021) Host immunity increases Mycobacterium tuberculosis reliance on cytochrome bd oxidase. *PLoS pathogens* 17: e1008911

Campbell PJ, Morlock GP, Sikes RD, Dalton TL, Metchock B, Starks AM, Hooks DP, Cowan LS, Plikaytis BB, Posey JE (2011) Molecular detection of mutations associated with first- and second-line drug resistance compared with conventional drug susceptibility testing of Mycobacterium tuberculosis. *Antimicrob Agents Chemother* 55: 2032-2041

Carette X, Platig J, Young DC, Helmel M, Young AT, Wang Z, Potluri LP, Moody CS, Zeng J, Prisic S *et al* (2018) Multisystem Analysis of Mycobacterium tuberculosis Reveals Kinase-Dependent Remodeling of the Pathogen-Environment Interface. *mBio* 9

Chatterji M, Shandil R, Manjunatha MR, Solapure S, Ramachandran V, Kumar N, Saralaya R, Panduga V, Reddy J, Prabhakar KR *et al* (2014) 1,4-azaindole, a potential drug candidate for treatment of tuberculosis. *Antimicrob Agents Chemother* 58: 5325-5331

Chawla Y, Upadhyay S, Khan S, Nagarajan SN, Forti F, Nandicoori VK (2014) Protein Kinase B ( PknB) of Mycobacterium tuberculosis Is Essential for Growth of the Pathogen in Vitro as well as for Survival within the Host. *Journal of Biological Chemistry* 289: 13858-13875

Chen W, Huang YJ, Gundala SR, Yang H, Li M, Tai PC, Wang B (2010) The first low microM SecA inhibitors. *Bioorg Med Chem* 18: 1617-1625

Chengalroyen MD, Mason MK, Borsellini A, Tassoni R, Abrahams GL, Lynch S, Ahn YM, Ambler J, Young K, Crowley BM *et al* (2022) DNA-Dependent Binding of Nargenicin to DnaE1 Inhibits Replication in Mycobacterium tuberculosis. *ACS Infect Dis* 8: 612-625

Chengalroyen MD, Mehaffy C, Lucas M, Bauer N, Raphela ML, Oketade N, Warner DF, Lewinsohn DA, Lewinsohn DM, Dobos KM *et al* (2024) Modulation of riboflavin biosynthesis and utilization in mycobacteria. *Microbiology spectrum* 12: e0320723

Cheung CY, McNeil MB, Cook GM (2022) Utilization of CRISPR interference to investigate the contribution of genes to pathogenesis in a macrophage model of Mycobacterium tuberculosis infection. *J Antimicrob Chemother* 77: 615-619

Chiarelli LR, Mori G, Orena BS, Esposito M, Lane T, de Jesus Lopes Ribeiro AL, Degiacomi G, Zemanova J, Szadocka S, Huszar S *et al* (2018) A multitarget approach to drug discovery inhibiting Mycobacterium tuberculosis PyrG and PanK. *Sci Rep* 8: 3187

Chopra S, Matsuyama K, Tran T, Malerich JP, Wan B, Franzblau SG, Lun S, Guo H, Maiga MC, Bishai WR *et al* (2012) Evaluation of gyrase B as a drug target in Mycobacterium tuberculosis. *J Antimicrob Chemother* 67: 415-421

Choudhary E, Sharma R, Kumar Y, Agarwal N (2019) Conditional Silencing by CRISPRi Reveals the Role of DNA Gyrase in Formation of Drug-Tolerant Persister Population in Mycobacterium tuberculosis. *Front Cell Infect Microbiol* 9: 70

Choules MP, Wolf NM, Lee H, Anderson JR, Grzelak EM, Wang Y, Ma R, Gao W, McAlpine JB, Jin YY *et al* (2019) Rufomycin Targets ClpC1 Proteolysis in Mycobacterium tuberculosis and M. abscessus. *Antimicrob Agents Chemother* 63

Conradie F, Diacon AH, Ngubane N, Howell P, Everitt D, Crook AM, Mendel CM, Egizi E, Moreira J, Timm J *et al* (2020) Treatment of Highly Drug-Resistant Pulmonary Tuberculosis. *N Engl J Med* 382: 893-902

Converse PJ, Karakousis PC, Klinkenberg LG, Kesavan AK, Ly LH, Allen SS, Grosset JH, Jain SK, Lamichhane G, Manabe YC *et al* (2009) Role of the dosR-dosS two-component regulatory system in Mycobacterium tuberculosis virulence in three animal models. *Infect Immun* 77: 1230-1237

Council MR (1948) STREPTOMYCIN treatment of pulmonary tuberculosis. *Br Med J* 2: 769-782

Courbon GM, Palme PR, Mann L, Richter A, Imming P, Rubinstein JL (2023) Mechanism of mycobacterial ATP synthase inhibition by squaramides and second generation diarylquinolines. *EMBO J* 42: e113687

Cox JA, Mugumbate G, Del Peral LV, Jankute M, Abrahams KA, Jervis P, Jackenkroll S, Perez A, Alemparte C, Esquivias J *et al* (2016) Novel inhibitors of Mycobacterium tuberculosis GuaB2 identified by a target based high-throughput phenotypic screen. *Sci Rep* 6: 38986

Cui P, Li X, Zhu M, Wang B, Liu J, Chen H (2017) Design, synthesis and antimicrobial activities of thiouracil derivatives containing triazolo-thiadiazole as SecA inhibitors. *Eur J Med Chem* 127: 159-165

Cunningham F, Esquivias J, Fernandez-Menendez R, Perez A, Guardia A, Escribano J, Rivero C, Vimal M, Cacho M, de Dios-Anton P *et al* (2020) Exploring the SAR of the beta-Ketoacyl-ACP Synthase Inhibitor GSK3011724A and Optimization around a Genotoxic Metabolite. *ACS Infect Dis* 6: 1098-1109

Dai R, Wilson DJ, Geders TW, Aldrich CC, Finzel BC (2014) Inhibition of Mycobacterium tuberculosis transaminase BioA by aryl hydrazines and hydrazides. *Chembiochem* 15: 575-586

Dal Molin M, Selchow P, Schafle D, Tschumi A, Ryckmans T, Laage-Witt S, Sander P (2019) Identification of novel scaffolds targeting Mycobacterium tuberculosis. *J Mol Med (Berl)* 97: 1601-1613

Davis SH, Mathieson M, Buchanan KI, Dawson A, Smith A, Cocco M, Tamaki FK, Post JM, Baragana B, Jansen C *et al* (2025) Design and Development of Lysyl tRNA Synthetase Inhibitors, for the Treatment of Tuberculosis. *J Med Chem* 68: 16459-16482

Dawadi S, Boshoff HIM, Park SW, Schnappinger D, Aldrich CC (2018) Conformationally Constrained Cinnolinone Nucleoside Analogues as Siderophore Biosynthesis Inhibitors for Tuberculosis. *ACS medicinal chemistry letters* 9: 386-391

Dawadi S, Kawamura S, Rubenstein A, Remmel R, Aldrich CC (2016) Synthesis and pharmacological evaluation of nucleoside prodrugs designed to target siderophore biosynthesis in Mycobacterium tuberculosis. *Bioorg Med Chem* 24: 1314-1321

Dawadi S, Viswanathan K, Boshoff HI, Barry CE, 3rd, Aldrich CC (2015) Investigation and conformational analysis of fluorinated nucleoside antibiotics targeting siderophore biosynthesis. *J Org Chem* 80: 4835-4850

de Jager VR, Dawson R, van Niekerk C, Hutchings J, Kim J, Vanker N, van der Merwe L, Choi J, Nam K, Diacon AH (2020) Telacebec (Q203), a New Antituberculosis Agent. *N Engl J Med* 382: 1280-1281

De Voss JJ, Rutter K, Schroeder BG, Su H, Zhu Y, Barry CE, 3rd (2000) The salicylate-derived mycobactin siderophores of Mycobacterium tuberculosis are essential for growth in macrophages. *Proceedings of the National Academy of Sciences of the United States of America* 97: 1252-1257

de Wet TJ, Winkler KR, Mhlanga M, Mizrahi V, Warner DF (2020) Arrayed CRISPRi and quantitative imaging describe the morphotypic landscape of essential mycobacterial genes. *Elife* 9

Dechow SJ, Abramovitch RB (2024) Targeting Mycobacterium tuberculosis pH-driven adaptation. *Microbiology (Reading)* 170

Degiacomi G, Benjak A, Madacki J, Boldrin F, Provvedi R, Palu G, Kordulakova J, Cole ST, Manganelli R (2017) Essentiality of mmpL3 and impact of its silencing on Mycobacterium tuberculosis gene expression. *Sci Rep* 7: 43495

DeJesus MA, Gerrick ER, Xu W, Park SW, Long JE, Boutte CC, Rubin EJ, Schnappinger D, Ehrt S, Fortune SM *et al* (2017) Comprehensive Essentiality Analysis of the Mycobacterium tuberculosis Genome via Saturating Transposon Mutagenesis. *MBio* 8

Diacon AH, Barry CE, 3rd, Carlton A, Chen RY, Davies M, de Jager V, Fletcher K, Koh G, Kontsevaya I, Heyckendorf J *et al* (2024) A first-in-class leucyl-tRNA synthetase inhibitor, ganfeborole, for rifampicin-susceptible tuberculosis: a phase 2a open-label, randomized trial. *Nat Med* 30: 896-904

Driessen AJ, Nouwen N (2008) Protein translocation across the bacterial cytoplasmic membrane. *Annu Rev Biochem* 77: 643-667

Duckworth BP, Geders TW, Tiwari D, Boshoff HI, Sibbald PA, Barry CE, 3rd, Schnappinger D, Finzel BC, Aldrich CC (2011) Bisubstrate adenylation inhibitors of biotin protein ligase from Mycobacterium tuberculosis. *Chemistry & biology* 18: 1432-1441

Economou A, Wickner W (1994) SecA promotes preprotein translocation by undergoing ATP-driven cycles of membrane insertion and deinsertion. *Cell* 78: 835-843

Ehrt S, Guo XV, Hickey CM, Ryou M, Monteleone M, Riley LW, Schnappinger D (2005) Controlling gene expression in mycobacteria with anhydrotetracycline and Tet repressor. *Nucleic Acids Res* 33: e21

Engelhart CA, Aldrich CC (2013) Synthesis of chromone, quinolone, and benzoxazinone sulfonamide nucleosides as conformationally constrained inhibitors of adenylating enzymes required for siderophore biosynthesis. *J Org Chem* 78: 7470-7481

Escuyer VE, Lety MA, Torrelles JB, Khoo KH, Tang JB, Rithner CD, Frehel C, McNeil MR, Brennan PJ, Chatterjee D (2001) The role of the embA and embB gene products in the biosynthesis of the terminal hexaarabinofuranosyl motif of Mycobacterium smegmatis arabinogalactan. *The Journal of biological chemistry* 276: 48854-48862

Esposito M, Szadocka S, Degiacomi G, Orena BS, Mori G, Piano V, Boldrin F, Zemanova J, Huszar S, Barros D *et al* (2017) A Phenotypic Based Target Screening Approach Delivers New Antitubercular CTP Synthetase Inhibitors. *ACS Infect Dis* 3: 428-437

Evans JC, Murugesan D, Post JM, Mendes V, Wang Z, Nahiyaan N, Lynch SL, Thompson S, Green SR, Ray PC *et al* (2021) Targeting Mycobacterium tuberculosis CoaBC through Chemical Inhibition of 4'-Phosphopantothenoyl-l-cysteine Synthetase (CoaB) Activity. *ACS Infect Dis* 7: 1666-1679

Evans JC, Trujillo C, Wang Z, Eoh H, Ehrt S, Schnappinger D, Boshoff HI, Rhee KY, Barry CE, 3rd, Mizrahi V (2016) Validation of CoaBC as a Bactericidal Target in the Coenzyme A Pathway of Mycobacterium tuberculosis. *ACS Infect Dis* 2: 958-968

Famulla K, Sass P, Malik I, Akopian T, Kandror O, Alber M, Hinzen B, Ruebsamen-Schaeff H, Kalscheuer R, Goldberg AL *et al* (2016) Acyldepsipeptide antibiotics kill mycobacteria by preventing the physiological functions of the ClpP1P2 protease. *Mol Microbiol* 101: 194-209

Fang C, Lee KK, Nietupski R, Bates RH, Fernandez-Menendez R, Lopez-Roman EM, Guijarro-Lopez L, Yin Y, Peng Z, Gomez JE *et al* (2018) Discovery of heterocyclic replacements for the coumarin core of anti-tubercular FadD32 inhibitors. *Bioorg Med Chem Lett* 28: 3529-3533

Feltcher ME, Sullivan JT, Braunstein M (2010) Protein export systems of Mycobacterium tuberculosis: novel targets for drug development? *Future Microbiol* 5: 1581-1597

Fernandez P, Saint-Joanis B, Barilone N, Jackson M, Gicquel B, Cole ST, Alzari PM (2006) The Ser/Thr protein kinase PknB is essential for sustaining mycobacterial growth. *Journal of Bacteriology* 188: 7778-7784

Ferreras JA, Ryu JS, Di Lello F, Tan DS, Quadri LE (2005) Small-molecule inhibition of siderophore biosynthesis in Mycobacterium tuberculosis and Yersinia pestis. *Nature chemical biology* 1: 29-32

Flipo M, Frita R, Bourotte M, Martinez-Martinez MS, Boesche M, Boyle GW, Derimanov G, Drewes G, Gamallo P, Ghidelli-Disse S *et al* (2022) The small-molecule SMARt751 reverses Mycobacterium tuberculosis resistance to ethionamide in acute and chronic mouse models of tuberculosis. *Sci Transl Med* 14: eaaz6280

Foley LH, Wang P, Dunten P, Ramsey G, Gubler ML, Wertheimer SJ (2003a) Modified 3-alkyl-1,8-dibenzylxanthines as GTP-competitive inhibitors of phosphoenolpyruvate carboxykinase. *Bioorg Med Chem Lett* 13: 3607-3610

Foley LH, Wang P, Dunten P, Ramsey G, Gubler ML, Wertheimer SJ (2003b) X-ray structures of two xanthine inhibitors bound to PEPCK and N-3 modifications of substituted 1,8-dibenzylxanthines. *Bioorg Med Chem Lett* 13: 3871-3874

Fu C, Liu Y, Walt C, Rasheed S, Bader CD, Lukat P, Neuber M, Haeckl FPJ, Blankenfeldt W, Kalinina OV *et al* (2024) Elucidation of unusual biosynthesis and DnaN-targeting mode of action of potent anti-tuberculosis antibiotics Mycoplanecins. *Nat Commun* 15: 791

Furin JJ, Du Bois J, van Brakel E, Chheng P, Venter A, Peloquin CA, Alsultan A, Thiel BA, Debanne SM, Boom WH *et al* (2016) Early Bactericidal Activity of AZD5847 in Patients with Pulmonary Tuberculosis. *Antimicrob Agents Chemother* 60: 6591-6599

Galina L, Hopf FSM, Abbadi BL, Sperotto NDM, Czeczot AM, Duque-Villegas MA, Perello MA, Matter LB, de Souza EV, Parish T *et al* (2022) Evaluation of 3-Deoxy-D-Arabino-Heptulosonate 7-Phosphate Synthase (DAHPS) as a Vulnerable Target in Mycobacterium tuberculosis. *Microbiol Spectr* 10: e0072822

Gandotra S, Schnappinger D, Monteleone M, Hillen W, Ehrt S (2007) In vivo gene silencing identifies the Mycobacterium tuberculosis proteasome as essential for the bacteria to persist in mice. *Nature medicine* 13: 1515-1520

Gao W, Kim JY, Anderson JR, Akopian T, Hong S, Jin YY, Kandror O, Kim JW, Lee IA, Lee SY *et al* (2015) The cyclic peptide ecumicin targeting ClpC1 is active against Mycobacterium tuberculosis in vivo. *Antimicrob Agents Chemother* 59: 880-889

Gautam US, McGillivray A, Mehra S, Didier PJ, Midkiff CC, Kissee RS, Golden NA, Alvarez X, Niu T, Rengarajan J *et al* (2015) DosS Is required for the complete virulence of mycobacterium tuberculosis in mice with classical granulomatous lesions. *Am J Respir Cell Mol Biol* 52: 708-716

Gavalda S, Leger M, van der Rest B, Stella A, Bardou F, Montrozier H, Chalut C, Burlet-Schiltz O, Marrakchi H, Daffe M *et al* (2009) The Pks13/FadD32 crosstalk for the biosynthesis of mycolic acids in Mycobacterium tuberculosis. *J Biol Chem* 284: 19255-19264

Gedeon A, Yab E, Dinut A, Sadowski E, Capton E, Dreneau A, Petit J, Gioia B, Piveteau C, Djaout K *et al* (2024) Molecular mechanism of a triazole-containing inhibitor of Mycobacterium tuberculosis DNA gyrase. *iScience* 27: 110967

Gierse RM, Reddem ER, Alhayek A, Baitinger D, Hamid Z, Jakobi H, Laber B, Lange G, Hirsch AKH, Groves MR (2021) Identification of a 1-deoxy-D-xylulose-5-phosphate synthase (DXS) mutant with improved crystallographic properties. *Biochem Biophys Res Commun* 539: 42-47

Ginn J, Jiang X, Sun S, Michino M, Huggins DJ, Mbambo Z, Jansen R, Rhee KY, Arango N, Lima CD *et al* (2021) Whole Cell Active Inhibitors of Mycobacterial Lipoamide Dehydrogenase Afford Selectivity over the Human Enzyme through Tight Binding Interactions. *ACS Infect Dis* 7: 435-444

Gl B, Rajput R, Gupta M, Dahiya P, Thakur JK, Bhatnagar R, Grover A (2020) Structure-based drug repurposing to inhibit the DNA gyrase of Mycobacterium tuberculosis. *Biochem J* 477: 4167-4190

Gordon O, Dikeman DA, Ortines RV, Wang Y, Youn C, Mumtaz M, Orlando N, Zhang J, Patel AM, Gough E *et al* (2022) The Novel Oxazolidinone TBI-223 Is Effective in Three Preclinical Mouse Models of Methicillin-Resistant Staphylococcus aureus Infection. *Microbiol Spectr* 10: e0245121

Grant SS, Wellington S, Kawate T, Desjardins CA, Silvis MR, Wivagg C, Thompson M, Gordon K, Kazyanskaya E, Nietupski R *et al* (2016) Baeyer-Villiger Monooxygenases EthA and MymA Are Required for Activation of Replicating and Non-replicating Mycobacterium tuberculosis Inhibitors. *Cell Chem Biol* 23: 666-677

Green LS, Bullard JM, Ribble W, Dean F, Ayers DF, Ochsner UA, Janjic N, Jarvis TC (2009) Inhibition of methionyl-tRNA synthetase by REP8839 and effects of resistance mutations on enzyme activity. *Antimicrob Agents Chemother* 53: 86-94

Green SR, Davis SH, Damerow S, Engelhart CA, Mathieson M, Baragana B, Robinson DA, Tamjar J, Dawson A, Tamaki FK *et al* (2022) Lysyl-tRNA synthetase, a target for urgently needed M. tuberculosis drugs. *Nat Commun* 13: 5992

Green SR, Harrison JR, Thompson S, Murugesan D, Libardo MDJ, Engelhart CA, Meshanni J, Fletcher D, Scullion P, Edwards D *et al* (2025) Identification of a Series Containing a Pentafluorophenyl Moiety That Targets Pks13 to Inhibit Growth of Mycobacterium tuberculosis. *ACS Infect Dis* 11: 715-726

Green SR, Wilson C, Eadsforth TC, Punekar AS, Tamaki FK, Wood G, Caldwell N, Forte B, Norcross NR, Kiczun M *et al* (2023) Identification and Optimization of Novel Inhibitors of the Polyketide Synthase 13 Thioesterase Domain with Antitubercular Activity. *J Med Chem* 66: 15380-15408

Griesser T, Wang R, Angona IP, Rogenmoser J, Obrist J, Schneider G, Sander P (2025) Selective inhibition of Mycobacterium tuberculosis GpsI unveils a novel strategy to target the RNA metabolism. *Nucleic acids research* 53

Grover S, Engelhart CA, Perez-Herran E, Li W, Abrahams KA, Papavinasasundaram K, Bean JM, Sassetti CM, Mendoza-Losana A, Besra GS *et al* (2021) Two-Way Regulation of MmpL3 Expression Identifies and Validates Inhibitors of MmpL3 Function in Mycobacterium tuberculosis. *Acs Infectious Diseases* 7: 141-152

Grzegorzewicz AE, Eynard N, Quemard A, North EJ, Margolis A, Lindenberger JJ, Jones V, Kordulakova J, Brennan PJ, Lee RE *et al* (2015) Covalent modification of the Mycobacterium tuberculosis FAS-II dehydratase by Isoxyl and Thiacetazone. *ACS Infect Dis* 1: 91-97

Grzegorzewicz AE, Pham H, Gundi VA, Scherman MS, North EJ, Hess T, Jones V, Gruppo V, Born SE, Kordulakova J *et al* (2012) Inhibition of mycolic acid transport across the Mycobacterium tuberculosis plasma membrane. *Nat Chem Biol* 8: 334-341

Gurcha SS, Usha V, Cox JA, Futterer K, Abrahams KA, Bhatt A, Alderwick LJ, Reynolds RC, Loman NJ, Nataraj V *et al* (2014) Biochemical and structural characterization of mycobacterial aspartyl-tRNA synthetase AspS, a promising TB drug target. *PLoS One* 9: e113568

Habjan E, Ho VQT, Gallant J, van Stempvoort G, Jim KK, Kuijl C, Geerke DP, Bitter W, Speer A (2021) An anti-tuberculosis compound screen using a zebrafish infection model identifies an aspartyl-tRNA synthetase inhibitor. *Dis Model Mech* 14

Hajian B, Scocchera E, Shoen C, Krucinska J, Viswanathan K, N GD, Erlandsen H, Estrada A, Mikusova K, Kordulakova J *et al* (2019) Drugging the Folate Pathway in Mycobacterium tuberculosis: The Role of Multi-targeting Agents. *Cell Chem Biol* 26: 781-791 e786

Haranahalli K, Tong S, Kim S, Awwa M, Chen L, Knudson SE, Slayden RA, Singleton E, Russo R, Connell N *et al* (2021) Structure-activity relationship studies on 2,5,6-trisubstituted benzimidazoles targeting Mtb-FtsZ as antitubercular agents. *RSC Med Chem* 12: 78-94

Hariguchi N, Chen X, Hayashi Y, Kawano Y, Fujiwara M, Matsuba M, Shimizu H, Ohba Y, Nakamura I, Kitamoto R *et al* (2020) OPC-167832, a Novel Carbostyril Derivative with Potent Antituberculosis Activity as a DprE1 Inhibitor. *Antimicrob Agents Chemother* 64

Harold LK, Jinich A, Hards K, Cordeiro A, Keighley LM, Cross A, McNeil MB, Rhee K, Cook GM (2022) Deciphering functional redundancy and energetics of malate oxidation in mycobacteria. *J Biol Chem* 298: 101859

Hartkoorn RC, Sala C, Neres J, Pojer F, Magnet S, Mukherjee R, Uplekar S, Boy-Rottger S, Altmann KH, Cole ST (2012) Towards a new tuberculosis drug: pyridomycin - nature's isoniazid. *EMBO Mol Med* 4: 1032-1042

Hartl FU, Lecker S, Schiebel E, Hendrick JP, Wickner W (1990) The binding cascade of SecB to SecA to SecY/E mediates preprotein targeting to the E. coli plasma membrane. *Cell* 63: 269-279

Hong W, Deng W, Xie J (2013) The structure, function, and regulation of Mycobacterium FtsZ. *Cell Biochem Biophys* 65: 97-105

Hsieh YH, Huang YJ, Jin JS, Yu L, Yang H, Jiang C, Wang B, Tai PC (2014) Mechanisms of Rose Bengal inhibition on SecA ATPase and ion channel activities. *Biochem Biophys Res Commun* 454: 308-312

Hsu HC, Singh PK, Fan H, Wang R, Sukenick G, Nathan C, Lin G, Li H (2017) Structural Basis for the Species-Selective Binding of N,C-Capped Dipeptides to the Mycobacterium tuberculosis Proteasome. *Biochemistry* 56: 324-333

Huang Q, Tonge PJ, Slayden RA, Kirikae T, Ojima I (2007) FtsZ: a novel target for tuberculosis drug discovery. *Curr Top Med Chem* 7: 527-543

Huang YJ, Wang H, Gao FB, Li M, Yang H, Wang B, Tai PC (2012) Fluorescein analogues inhibit SecA ATPase: the first sub-micromolar inhibitor of bacterial protein translocation. *ChemMedChem* 7: 571-577

Imai Y, Hauk G, Quigley J, Liang L, Son S, Ghiglieri M, Gates MF, Morrissette M, Shahsavari N, Niles S *et al* (2022) Evybactin is a DNA gyrase inhibitor that selectively kills Mycobacterium tuberculosis. *Nat Chem Biol* 18: 1236-1244

Janssen S, Upton C, de Jager VR, van Niekerk C, Dawson R, Hutchings J, Kim J, Choi J, Nam K, Sun E *et al* (2025) Telacebec, a Potent Agent in the Fight against Tuberculosis: Findings from a Randomized, Phase 2 Clinical Trial and Beyond. *Am J Respir Crit Care Med* 211: 1504-1512

Jeffreys LN, Ardrey A, Hafiz TA, Dyer LA, Warman AJ, Mosallam N, Nixon GL, Fisher NE, Hong WD, Leung SC *et al* (2023) Identification of 2-Aryl-Quinolone Inhibitors of Cytochrome bd and Chemical Validation of Combination Strategies for Respiratory Inhibitors against Mycobacterium tuberculosis. *ACS Infect Dis* 9: 221-238

Johnson EO, LaVerriere E, Office E, Stanley M, Meyer E, Kawate T, Gomez JE, Audette RE, Bandyopadhyay N, Betancourt N *et al* (2019) Large-scale chemical-genetics yields new M. tuberculosis inhibitor classes. *Nature* 571: 72-78

Johnson EO, Office E, Kawate T, Orzechowski M, Hung DT (2020) Large-Scale Chemical-Genetic Strategy Enables the Design of Antimicrobial Combination Chemotherapy in Mycobacteria. *ACS Infect Dis* 6: 56-63

Johnston JM, Arcus VL, Morton CJ, Parker MW, Baker EN (2003) Crystal structure of a putative methyltransferase from Mycobacterium tuberculosis: misannotation of a genome clarified by protein structural analysis. *Journal of bacteriology* 185: 4057-4065

Junne T, Wong J, Studer C, Aust T, Bauer BW, Beibel M, Bhullar B, Bruccoleri R, Eichenberger J, Estoppey D *et al* (2015) Decatransin, a new natural product inhibiting protein translocation at the Sec61/SecYEG translocon. *J Cell Sci* 128: 1217-1229

Kalia NP, Hasenoehrl EJ, Ab Rahman NB, Koh VH, Ang MLT, Sajorda DR, Hards K, Gruber G, Alonso S, Cook GM *et al* (2017) Exploiting the synthetic lethality between terminal respiratory oxidases to kill Mycobacterium tuberculosis and clear host infection. *Proceedings of the National Academy of Sciences of the United States of America* 114: 7426-7431

Kashyap A, Singh PK, Silakari O (2018) Chemical classes targeting energy supplying GyrB domain of Mycobacterium tuberculosis. *Tuberculosis (Edinb)* 113: 43-54

Khandelwal NK, Gupta M, Gomez JE, Barkho S, Guan Z, Eng AY, Kawate T, Balasubramani SG, Sali A, Hung DT *et al* (2025) Structure and inhibition mechanisms of Mycobacterium tuberculosis essential transporter efflux protein A. *Nature communications* 16: 3139

Khola S, Kumar S, Bhanwala N, Khatik GL (2024) Polyketide Synthase 13 (Pks13) Inhibition: A Potential Target for New Class of Anti-tubercular Agents. *Curr Top Med Chem* 24: 2362-2376

Kim DH, Lees WJ (2025) Molecular Pharmacology of the Antibiotic Fosfomycin, an Inhibitor of Peptidoglycan Biosynthesis. *Biochemistry* 64: 1720-1727

Kim JH, O'Brien KM, Sharma R, Boshoff HI, Rehren G, Chakraborty S, Wallach JB, Monteleone M, Wilson DJ, Aldrich CC *et al* (2013) A genetic strategy to identify targets for the development of drugs that prevent bacterial persistence. *Proceedings of the National Academy of Sciences of the United States of America* 110: 19095-19100

Kim JS, Kim YH, Lee SH, Kim YH, Kim JW, Kang JY, Kim SK, Kim SJ, Kang YS, Kim TH *et al* (2022) Early Bactericidal Activity of Delpazolid (LCB01-0371) in Patients with Pulmonary Tuberculosis. *Antimicrob Agents Chemother* 66: e0168421

Kirsch SH, Haeckl FPJ, Muller R (2022) Beyond the approved: target sites and inhibitors of bacterial RNA polymerase from bacteria and fungi. *Nat Prod Rep* 39: 1226-1263

Klein M, Hofmann B, Klose M, Freudl R (1994) Isolation and characterization of a Bacillus subtilis secA mutant allele conferring resistance to sodium azide. *FEMS Microbiol Lett* 124: 393-397

Kling A, Lukat P, Almeida DV, Bauer A, Fontaine E, Sordello S, Zaburannyi N, Herrmann J, Wenzel SC, Konig C *et al* (2015) Antibiotics. Targeting DnaN for tuberculosis therapy using novel griselimycins. *Science* 348: 1106-1112

Knoll KE, van der Walt MM, Loots DT (2022) In Silico Drug Discovery Strategies Identified ADMET Properties of Decoquinate RMB041 and Its Potential Drug Targets against Mycobacterium tuberculosis. *Microbiol Spectr* 10: e0231521

Knudson SE, Awasthi D, Kumar K, Carreau A, Goullieux L, Lagrange S, Vermet H, Ojima I, Slayden RA (2015) Cell division inhibitors with efficacy equivalent to isoniazid in the acute murine Mycobacterium tuberculosis infection model. *Journal of Antimicrobial Chemotherapy* 70: 3070-3073

Knudson SE, Kumar K, Awasthi D, Ojima I, Slayden RA (2014) In vitro-in vivo activity relationship of substituted benzimidazole cell division inhibitors with activity against Mycobacterium tuberculosis. *Tuberculosis* 94: 271-276

Koh EI, Oluoch PO, Ruecker N, Proulx MK, Soni V, Murphy KC, Papavinasasundaram K, Reames CJ, Trujillo C, Zaveri A *et al* (2022) Chemical-genetic interaction mapping links carbon metabolism and cell wall structure to tuberculosis drug efficacy. *Proc Natl Acad Sci U S A* 119: e2201632119

Kolly GS, Boldrin F, Sala C, Dhar N, Hartkoorn RC, Ventura M, Serafini A, McKinney JD, Manganelli R, Cole ST (2014) Assessing the essentiality of the decaprenyl-phospho-d-arabinofuranose pathway in Mycobacterium tuberculosis using conditional mutants. *Mol Microbiol* 92: 194-211

Korkegian A, Roberts DM, Blair R, Parish T (2014) Mutations in the essential arabinosyltransferase EmbC lead to alterations in Mycobacterium tuberculosis lipoarabinomannan. *The Journal of biological chemistry* 289: 35172-35181

Koul A, Vranckx L, Dhar N, Gohlmann HW, Ozdemir E, Neefs JM, Schulz M, Lu P, Mortz E, McKinney JD *et al* (2014) Delayed bactericidal response of Mycobacterium tuberculosis to bedaquiline involves remodelling of bacterial metabolism. *Nature communications* 5: 3369

Krajczyk A, Zeidler J, Januszczyk P, Dawadi S, Boshoff HI, Barry CE, 3rd, Ostrowski T, Aldrich CC (2016) 2-Aryl-8-aza-3-deazaadenosine analogues of 5'-O-[N-(salicyl)sulfamoyl]adenosine: Nucleoside antibiotics that block siderophore biosynthesis in Mycobacterium tuberculosis. *Bioorg Med Chem* 24: 3133-3143

Kremer L, Douglas JD, Baulard AR, Morehouse C, Guy MR, Alland D, Dover LG, Lakey JH, Jacobs WR, Jr., Brennan PJ *et al* (2000) Thiolactomycin and related analogues as novel anti-mycobacterial agents targeting KasA and KasB condensing enzymes in Mycobacterium tuberculosis. *J Biol Chem* 275: 16857-16864

Krieger IV, Freundlich JS, Gawandi VB, Roberts JP, Gawandi VB, Sun Q, Owen JL, Fraile MT, Huss SI, Lavandera JL *et al* (2012) Structure-guided discovery of phenyl-diketo acids as potent inhibitors of M. tuberculosis malate synthase. *Chem Biol* 19: 1556-1567

Krieger IV, Sukheja P, Yang B, Tang S, Selle D, Woods A, Engelhart C, Kumar P, Harbut MB, Liu D *et al* (2025) SuFEx-based antitubercular compound irreversibly inhibits Pks13. *Nature* 645: 755-763

Krieger IV, Yalamanchili S, Dickson P, Engelhart CA, Zimmerman MD, Wood J, Clary E, Nguyen J, Thornton N, Centrella PA *et al* (2024) Inhibitors of the Thioesterase Activity of Mycobacterium tuberculosis Pks13 Discovered Using DNA-Encoded Chemical Library Screening. *ACS Infect Dis* 10: 1561-1575

Kumar K, Awasthi D, Lee SY, Zanardi I, Ruzsicska B, Knudson S, Tonge PJ, Slayden RA, Ojima I (2011) Novel Trisubstituted Benzimidazoles, Targeting Mtb FtsZ, as a New Class of Antitubercular Agents. *Journal of Medicinal Chemistry* 54: 374-381

Lamprecht DA, Wall RJ, Leemans A, Truebody B, Sprangers J, Fiogbe P, Davies C, Wetzel J, Daems S, Pearson W *et al* (2025) Targeting de novo purine biosynthesis for tuberculosis treatment. *Nature* 644: 214-220

Leblanc C, Prudhomme T, Tabouret G, Ray A, Burbaud S, Cabantous S, Mourey L, Guilhot C, Chalut C (2012) 4'-Phosphopantetheinyl transferase PptT, a new drug target required for Mycobacterium tuberculosis growth and persistence in vivo. *PLoS pathogens* 8: e1003097

Lee BS, Hards K, Engelhart CA, Hasenoehrl EJ, Kalia NP, Mackenzie JS, Sviriaeva E, Chong SMS, Manimekalai MSS, Koh VH *et al* (2021) Dual inhibition of the terminal oxidases eradicates antibiotic-tolerant Mycobacterium tuberculosis. *EMBO Mol Med* 13: e13207

Lee RE, Hurdle JG, Liu J, Bruhn DF, Matt T, Scherman MS, Vaddady PK, Zheng Z, Qi J, Akbergenov R *et al* (2014) Spectinamides: a new class of semisynthetic antituberculosis agents that overcome native drug efflux. *Nat Med* 20: 152-158

Leger M, Gavalda S, Guillet V, van der Rest B, Slama N, Montrozier H, Mourey L, Quemard A, Daffe M, Marrakchi H (2009) The dual function of the Mycobacterium tuberculosis FadD32 required for mycolic acid biosynthesis. *Chem Biol* 16: 510-519

Lempens P, Meehan CJ, Vandelannoote K, Fissette K, de Rijk P, Van Deun A, Rigouts L, de Jong BC (2018) Isoniazid resistance levels of Mycobacterium tuberculosis can largely be predicted by high-confidence resistance-conferring mutations. *Sci Rep* 8: 3246

Li D, Zhang X, Yao Y, Sun X, Sun J, Ma X, Yuan K, Bai G, Pang X, Hua R *et al* (2024) Structure and function of Mycobacterium tuberculosis EfpA as a lipid transporter and its inhibition by BRD-8000.3. *Proceedings of the National Academy of Sciences of the United States of America* 121: e2412653121

Li M, Huang YJ, Tai PC, Wang B (2008) Discovery of the first SecA inhibitors using structure-based virtual screening. *Biochem Biophys Res Commun* 368: 839-845

Li S, Poulton NC, Chang JS, Azadian ZA, DeJesus MA, Ruecker N, Zimmerman MD, Eckartt KA, Bosch B, Engelhart CA *et al* (2022) CRISPRi chemical genetics and comparative genomics identify genes mediating drug potency in Mycobacterium tuberculosis. *Nat Microbiol* 7: 766-779

Li W, Obregon-Henao A, Wallach JB, North EJ, Lee RE, Gonzalez-Juarrero M, Schnappinger D, Jackson M (2016) Therapeutic Potential of the Mycobacterium tuberculosis Mycolic Acid Transporter, MmpL3. *Antimicrob Agents Chemother* 60: 5198-5207

Li W, Upadhyay A, Fontes FL, North EJ, Wang Y, Crans DC, Grzegorzewicz AE, Jones V, Franzblau SG, Lee RE *et al* (2014) Novel insights into the mechanism of inhibition of MmpL3, a target of multiple pharmacophores in Mycobacterium tuberculosis. *Antimicrob Agents Chemother* 58: 6413-6423

Li X, Hernandez V, Rock FL, Choi W, Mak YSL, Mohan M, Mao W, Zhou Y, Easom EE, Plattner JJ *et al* (2017) Discovery of a Potent and Specific M. tuberculosis Leucyl-tRNA Synthetase Inhibitor: (S)-3-(Aminomethyl)-4-chloro-7-(2-hydroxyethoxy)benzo[c][1,2]oxaborol-1(3H)-ol (GSK656). *J Med Chem* 60: 8011-8026

Lin G, Chidawanyika T, Tsu C, Warrier T, Vaubourgeix J, Blackburn C, Gigstad K, Sintchak M, Dick L, Nathan C (2013) N,C-Capped dipeptides with selectivity for mycobacterial proteasome over human proteasomes: role of S3 and S1 binding pockets. *J Am Chem Soc* 135: 9968-9971

Lin G, Li D, de Carvalho LP, Deng H, Tao H, Vogt G, Wu K, Schneider J, Chidawanyika T, Warren JD *et al* (2009) Inhibitors selective for mycobacterial versus human proteasomes. *Nature* 461: 621-626

Lin G, Tsu C, Dick L, Zhou XK, Nathan C (2008) Distinct specificities of Mycobacterium tuberculosis and mammalian proteasomes for N-acetyl tripeptide substrates. *The Journal of biological chemistry* 283: 34423-34431

Lin W, Mandal S, Degen D, Liu Y, Ebright YW, Li S, Feng Y, Zhang Y, Mandal S, Jiang Y *et al* (2017) Structural Basis of Mycobacterium tuberculosis Transcription and Transcription Inhibition. *Mol Cell* 66: 169-179 e168

Lin Y, Zhang H, Zhu N, Wang X, Han Y, Chen M, Jiang J, Si S (2018) Identification of TB-E12 as a novel FtsZ inhibitor with anti-tuberculosis activity. *Tuberculosis (Edinb)* 110: 79-85

Liu F, Dawadi S, Maize KM, Dai R, Park SW, Schnappinger D, Finzel BC, Aldrich CC (2017) Structure-Based Optimization of Pyridoxal 5'-Phosphate-Dependent Transaminase Enzyme (BioA) Inhibitors that Target Biotin Biosynthesis in Mycobacterium tuberculosis. *Journal of medicinal chemistry* 60: 5507-5520

Liu Q, Wallach JB, Jayasinghe YP, Sullivan MR, Proietto J, Rodriguez S, Vo S, Boshoff HIM, Jia Z, Ostrer L *et al* (2025) Structure-Guided Development of a Potent BioA Inhibitor Validates Biotin Synthesis Inhibition as a Therapeutic Strategy for Tuberculosis. *bioRxiv*

Lougheed KEA, Osborne SA, Saxty B, Whalley D, Chapman T, Bouloc N, Chugh J, Nott TJ, Patel D, Spivey VL *et al* (2011) Effective inhibitors of the essential kinase PknB and their potential as anti-mycobacterial agents. *Tuberculosis* 91: 277-286

Lun S, Guo H, Adamson J, Cisar JS, Davis TD, Chavadi SS, Warren JD, Quadri LE, Tan DS, Bishai WR (2013) Pharmacokinetic and in vivo efficacy studies of the mycobactin biosynthesis inhibitor salicyl-AMS in mice. *Antimicrobial agents and chemotherapy* 57: 5138-5140

Lunge A, Gupta R, Choudhary E, Agarwal N (2020) The unfoldase ClpC1 of Mycobacterium tuberculosis regulates the expression of a distinct subset of proteins having intrinsically disordered termini. *J Biol Chem* 295: 9455-9473

Machova I, Hubalek M, Lepsik M, Bednarova L, Pazderkova M, Kopecky V, Jr., Snasel J, Dostal J, Pichova I (2017) The Role of Cysteine Residues in Catalysis of Phosphoenolpyruvate Carboxykinase from Mycobacterium tuberculosis. *PLoS One* 12: e0170373

Machova I, Snasel J, Dostal J, Brynda J, Fanfrlik J, Singh M, Tarabek J, Vanek O, Bednarova L, Pichova I (2015) Structural and functional studies of phosphoenolpyruvate carboxykinase from Mycobacterium tuberculosis. *PLoS One* 10: e0120682

Maitra A, Munshi T, Healy J, Martin LT, Vollmer W, Keep NH, Bhakta S (2019) Cell wall peptidoglycan in Mycobacterium tuberculosis: An Achilles' heel for the TB-causing pathogen. *FEMS microbiology reviews* 43: 548-575

Makarov V, Manina G, Mikusova K, Mollmann U, Ryabova O, Saint-Joanis B, Dhar N, Pasca MR, Buroni S, Lucarelli AP *et al* (2009) Benzothiazinones kill Mycobacterium tuberculosis by blocking arabinan synthesis. *Science* 324: 801-804

Manjunatha UH, SP SR, Kondreddi RR, Noble CG, Camacho LR, Tan BH, Ng SH, Ng PS, Ma NL, Lakshminarayana SB *et al* (2015) Direct inhibitors of InhA are active against Mycobacterium tuberculosis. *Sci Transl Med* 7: 269ra263

Mann S, Marquet A, Ploux O (2005) Inhibition of 7,8-diaminopelargonic acid aminotransferase by amiclenomycin and analogues. *Biochem Soc Trans* 33: 802-805

Marrero J, Rhee KY, Schnappinger D, Pethe K, Ehrt S (2010) Gluconeogenic carbon flow of tricarboxylic acid cycle intermediates is critical for Mycobacterium tuberculosis to establish and maintain infection. *Proc Natl Acad Sci U S A* 107: 9819-9824

Martinez G, Tolentino K, Sukheja P, Webb J, McNamara CW, Chatterjee AK, Yang B (2025) Novel isoxazole thiophene-containing compounds active against Mycobacterium tuberculosis. *Bioorg Med Chem Lett* 119: 130108

Masini T, Lacy B, Monjas L, Hawksley D, de Voogd AR, Illarionov B, Iqbal A, Leeper FJ, Fischer M, Kontoyianni M *et al* (2015) Validation of a homology model of Mycobacterium tuberculosis DXS: rationalization of observed activities of thiamine derivatives as potent inhibitors of two orthologues of DXS. *Org Biomol Chem* 13: 11263-11277

Maus CE, Plikaytis BB, Shinnick TM (2005) Mutation of tlyA confers capreomycin resistance in Mycobacterium tuberculosis. *Antimicrob Agents Chemother* 49: 571-577

McNeil MB, Cook GM (2019) Utilization of CRISPR Interference To Validate MmpL3 as a Drug Target in Mycobacterium tuberculosis. *Antimicrob Agents Chemother* 63

McNeil MB, O'Malley T, Dennison D, Shelton CD, Sunde B, Parish T (2020) Multiple Mutations in Mycobacterium tuberculosis MmpL3 Increase Resistance to MmpL3 Inhibitors. *mSphere* 5

Mehra S, Foreman TW, Didier PJ, Ahsan MH, Hudock TA, Kissee R, Golden NA, Gautam US, Johnson AM, Alvarez X *et al* (2015) The DosR Regulon Modulates Adaptive Immunity and Is Essential for Mycobacterium tuberculosis Persistence. *Am J Respir Crit Care Med* 191: 1185-1196

Mendes V, Green SR, Evans JC, Hess J, Blaszczyk M, Spry C, Bryant O, Cory-Wright J, Chan DS, Torres PHM *et al* (2021) Inhibiting Mycobacterium tuberculosis CoaBC by targeting an allosteric site. *Nature communications* 12: 143

Mikusova K, Huang H, Yagi T, Holsters M, Vereecke D, D'Haeze W, Scherman MS, Brennan PJ, McNeil MR, Crick DC (2005) Decaprenylphosphoryl arabinofuranose, the donor of the D-arabinofuranosyl residues of mycobacterial arabinan, is formed via a two-step epimerization of decaprenylphosphoryl ribose. *J Bacteriol* 187: 8020-8025

Miller BK, Zulauf KE, Braunstein M (2017) The Sec Pathways and Exportomes of Mycobacterium tuberculosis. *Microbiol Spectr* 5

Minato Y, Thiede JM, Kordus SL, McKlveen EJ, Turman BJ, Baughn AD (2015) Mycobacterium tuberculosis folate metabolism and the mechanistic basis for para-aminosalicylic acid susceptibility and resistance. *Antimicrob Agents Chemother* 59: 5097-5106

Molodtsov V, Scharf NT, Stefan MA, Garcia GA, Murakami KS (2017) Structural basis for rifamycin resistance of bacterial RNA polymerase by the three most clinically important RpoB mutations found in Mycobacterium tuberculosis. *Mol Microbiol* 103: 1034-1045

Morayya S, Awasthy D, Yadav R, Ambady A, Sharma U (2015) Revisiting the essentiality of glutamate racemase in Mycobacterium tuberculosis. *Gene* 555: 269-276

Mori M, Sammartino JC, Costantino L, Gelain A, Meneghetti F, Villa S, Chiarelli LR (2019) An Overview on the Potential Antimycobacterial Agents Targeting Serine/Threonine Protein Kinases from Mycobacterium tuberculosis. *Curr Top Med Chem* 19: 646-661

Mori S, Yamasaki M, Maruyama Y, Momma K, Kawai S, Hashimoto W, Mikami B, Murata K (2005) NAD-binding mode and the significance of intersubunit contact revealed by the crystal structure of Mycobacterium tuberculosis NAD kinase-NAD complex. *Biochem Biophys Res Commun* 327: 500-508

Mosaei H, Zenkin N (2020) Inhibition of RNA Polymerase by Rifampicin and Rifamycin-Like Molecules. *EcoSal Plus* 9

Mukhopadhyay B, Concar EM, Wolfe RS (2001) A GTP-dependent vertebrate-type phosphoenolpyruvate carboxykinase from Mycobacterium smegmatis. *J Biol Chem* 276: 16137-16145

Mulye M, Jain V (2025) Molecular Insights Into the Structure, Function, and Stability of the DNA Polymerase Processivity Factor From Mycobacterium tuberculosis. *J Mol Biol* 437: 169416

Nagarajan SN, Upadhyay S, Chawla Y, Khan S, Naz S, Subramanian J, Gandotra S, Nandicoori VK (2015) Protein kinase A (PknA) of Mycobacterium tuberculosis is independently activated and is critical for growth in vitro and survival of the pathogen in the host. *J Biol Chem* 290: 9626-9645

Nelson KM, Viswanathan K, Dawadi S, Duckworth BP, Boshoff HI, Barry CE, 3rd, Aldrich CC (2015) Synthesis and Pharmacokinetic Evaluation of Siderophore Biosynthesis Inhibitors for Mycobacterium tuberculosis. *Journal of medicinal chemistry* 58: 5459-5475

Neres J, Pojer F, Molteni E, Chiarelli LR, Dhar N, Boy-Rottger S, Buroni S, Fullam E, Degiacomi G, Lucarelli AP *et al* (2012) Structural basis for benzothiazinone-mediated killing of Mycobacterium tuberculosis. *Sci Transl Med* 4: 150ra121

Nixon MR, Saionz KW, Koo MS, Szymonifka MJ, Jung H, Roberts JP, Nandakumar M, Kumar A, Liao R, Rustad T *et al* (2014) Folate pathway disruption leads to critical disruption of methionine derivatives in Mycobacterium tuberculosis. *Chem Biol* 21: 819-830

North EJ, Jackson M, Lee RE (2014) New approaches to target the mycolic acid biosynthesis pathway for the development of tuberculosis therapeutics. *Curr Pharm Des* 20: 4357-4378

Oliver DB, Cabelli RJ, Dolan KM, Jarosik GP (1990) Azide-resistant mutants of Escherichia coli alter the SecA protein, an azide-sensitive component of the protein export machinery. *Proc Natl Acad Sci U S A* 87: 8227-8231

Ollinger J, O'Malley T, Ahn J, Odingo J, Parish T (2012) Inhibition of the sole type I signal peptidase of Mycobacterium tuberculosis is bactericidal under replicating and nonreplicating conditions. *J Bacteriol* 194: 2614-2619

Osterman AL, Rodionova I, Li X, Sergienko E, Ma CT, Catanzaro A, Pettigrove ME, Reed RW, Gupta R, Rohde KH *et al* (2019) Novel Antimycobacterial Compounds Suppress NAD Biogenesis by Targeting a Unique Pocket of NaMN Adenylyltransferase. *ACS Chem Biol* 14: 949-958

Ottavi S, Li K, Cacioppo JG, Perkowski AJ, Ramesh R, Gold BS, Ling Y, Roberts J, Singh A, Zhang D *et al* (2023) Mycobacterium tuberculosis PptT Inhibitors Based on Heterocyclic Replacements of Amidinoureas. *ACS medicinal chemistry letters* 14: 970-976

Ottavi S, Scarry SM, Mosior J, Ling Y, Roberts J, Singh A, Zhang D, Goullieux L, Roubert C, Bacque E *et al* (2022) In Vitro and In Vivo Inhibition of the Mycobacterium tuberculosis Phosphopantetheinyl Transferase PptT by Amidinoureas. *Journal of medicinal chemistry* 65: 1996-2022

Paetzel M, Karla A, Strynadka NC, Dalbey RE (2002) Signal peptidases. *Chem Rev* 102: 4549-4580

Palencia A, Li X, Bu W, Choi W, Ding CZ, Easom EE, Feng L, Hernandez V, Houston P, Liu L *et al* (2016) Discovery of Novel Oral Protein Synthesis Inhibitors of Mycobacterium tuberculosis That Target Leucyl-tRNA Synthetase. *Antimicrob Agents Chemother* 60: 6271-6280

Parish CA, de la Cruz M, Smith SK, Zink D, Baxter J, Tucker-Samaras S, Collado J, Platas G, Bills G, Diez MT *et al* (2009) Antisense-guided isolation and structure elucidation of pannomycin, a substituted cis-decalin from Geomyces pannorum. *J Nat Prod* 72: 59-62

Parish T, Stoker NG (2002) The common aromatic amino acid biosynthesis pathway is essential in Mycobacterium tuberculosis. *Microbiology (Reading)* 148: 3069-3077

Park SW, Casalena DE, Wilson DJ, Dai R, Nag PP, Liu F, Boyce JP, Bittker JA, Schreiber SL, Finzel BC *et al* (2015) Target-based identification of whole-cell active inhibitors of biotin biosynthesis in Mycobacterium tuberculosis. *Chemistry & biology* 22: 76-86

Park Y, Pacitto A, Bayliss T, Cleghorn LA, Wang Z, Hartman T, Arora K, Ioerger TR, Sacchettini J, Rizzi M *et al* (2017) Essential but Not Vulnerable: Indazole Sulfonamides Targeting Inosine Monophosphate Dehydrogenase as Potential Leads against Mycobacterium tuberculosis. *ACS Infect Dis* 3: 18-33

Pavelka MS, Jr., Chen B, Kelley CL, Collins FM, Jacobs WR, Jr. (2003) Vaccine efficacy of a lysine auxotroph of Mycobacterium tuberculosis. *Infect Immun* 71: 4190-4192

Pethe K, Bifani P, Jang J, Kang S, Park S, Ahn S, Jiricek J, Jung J, Jeon HK, Cechetto J *et al* (2013) Discovery of Q203, a potent clinical candidate for the treatment of tuberculosis. *Nature medicine* 19: 1157-1160

Pieren M, Abaigar Gutierrez-Solana A, Antonijoan Arbos RM, Boyle GW, Davila M, Davy M, Gitzinger M, Husband L, Martinez-Martinez MS, Mazarro DO *et al* (2024) First-in-human study of alpibectir (BVL-GSK098), a novel potent anti-TB drug. *J Antimicrob Chemother* 79: 1353-1361

Poce G, Bates RH, Alfonso S, Cocozza M, Porretta GC, Ballell L, Rullas J, Ortega F, De Logu A, Agus E *et al* (2013) Improved BM212 MmpL3 inhibitor analogue shows efficacy in acute murine model of tuberculosis infection. *PLoS One* 8: e56980

Portevin D, De Sousa-D'Auria C, Houssin C, Grimaldi C, Chami M, Daffe M, Guilhot C (2004) A polyketide synthase catalyzes the last condensation step of mycolic acid biosynthesis in mycobacteria and related organisms. *Proc Natl Acad Sci U S A* 101: 314-319

Portevin D, de Sousa-D'Auria C, Montrozier H, Houssin C, Stella A, Laneelle MA, Bardou F, Guilhot C, Daffe M (2005) The acyl-AMP ligase FadD32 and AccD4-containing acyl-CoA carboxylase are required for the synthesis of mycolic acids and essential for mycobacterial growth: identification of the carboxylation product and determination of the acyl-CoA carboxylase components. *J Biol Chem* 280: 8862-8874

Prosser GA, Rodenburg A, Khoury H, de Chiara C, Howell S, Snijders AP, de Carvalho LP (2016) Glutamate Racemase Is the Primary Target of beta-Chloro-d-Alanine in Mycobacterium tuberculosis. *Antimicrobial agents and chemotherapy* 60: 6091-6099

Puckett S, Trujillo C, Wang Z, Eoh H, Ioerger TR, Krieger I, Sacchettini J, Schnappinger D, Rhee KY, Ehrt S (2017) Glyoxylate detoxification is an essential function of malate synthase required for carbon assimilation in Mycobacterium tuberculosis. *Proc Natl Acad Sci U S A* 114: E2225-E2232

Qu D, Ge P, Botella L, Park SW, Lee HN, Thornton N, Bean JM, Krieger IV, Sacchettini JC, Ehrt S *et al* (2024) Mycobacterial biotin synthases require an auxiliary protein to convert dethiobiotin into biotin. *Nature communications* 15: 4161

Quemard A, Sacchettini JC, Dessen A, Vilcheze C, Bittman R, Jacobs WR, Jr., Blanchard JS (1995) Enzymatic characterization of the target for isoniazid in Mycobacterium tuberculosis. *Biochemistry* 34: 8235-8241

Quigley J, Peoples A, Sarybaeva A, Hughes D, Ghiglieri M, Achorn C, Desrosiers A, Felix C, Liang L, Malveira S *et al* (2020) Novel Antimicrobials from Uncultured Bacteria Acting against Mycobacterium tuberculosis. *mBio* 11

Rani N, Rajmani RS, Surolia A (2025) Identification of an Isoxazole Derivative as an Antitubercular Compound for Targeting the FadD Enzymes of Mycobacterium tuberculosis. *J Med Chem* 68: 270-286

Rani N, Surolia A (2024) Targeted suppression of MEP pathway genes DXS, IspD and IspF to explore the mycobacterial metabolism and survival. *Int J Biol Macromol* 272: 132727

Reddy BK, Landge S, Ravishankar S, Patil V, Shinde V, Tantry S, Kale M, Raichurkar A, Menasinakai S, Mudugal NV *et al* (2014) Assessment of Mycobacterium tuberculosis pantothenate kinase vulnerability through target knockdown and mechanistically diverse inhibitors. *Antimicrobial agents and chemotherapy* 58: 3312-3326

Riccardi G, Pasca MR, Chiarelli LR, Manina G, Mattevi A, Binda C (2013) The DprE1 enzyme, one of the most vulnerable targets of Mycobacterium tuberculosis. *Appl Microbiol Biotechnol* 97: 8841-8848

Richter A, Rudolph I, Mollmann U, Voigt K, Chung CW, Singh OMP, Rees M, Mendoza-Losana A, Bates R, Ballell L *et al* (2018) Novel insight into the reaction of nitro, nitroso and hydroxylamino benzothiazinones and of benzoxacinones with Mycobacterium tuberculosis DprE1. *Sci Rep* 8: 13473

Rigel NW, Gibbons HS, McCann JR, McDonough JA, Kurtz S, Braunstein M (2009) The Accessory SecA2 System of Mycobacteria Requires ATP Binding and the Canonical SecA1. *J Biol Chem* 284: 9927-9936

Rittershaus ESC, Baek SH, Krieger IV, Nelson SJ, Cheng YS, Nambi S, Baker RE, Leszyk JD, Shaffer SA, Sacchettini JC *et al* (2018) A Lysine Acetyltransferase Contributes to the Metabolic Adaptation to Hypoxia in Mycobacterium tuberculosis. *Cell Chem Biol* 25: 1495-1505 e1493

Rock JM, Hopkins FF, Chavez A, Diallo M, Chase MR, Gerrick ER, Pritchard JR, Church GM, Rubin EJ, Sassetti CM *et al* (2017) Programmable transcriptional repression in mycobacteria using an orthogonal CRISPR interference platform. *Nat Microbiol* 2: 16274

Rodionova IA, Schuster BM, Guinn KM, Sorci L, Scott DA, Li X, Kheterpal I, Shoen C, Cynamon M, Locher C *et al* (2014) Metabolic and bactericidal effects of targeted suppression of NadD and NadE enzymes in mycobacteria. *MBio* 5

Rozwarski DA, Grant GA, Barton DH, Jacobs WR, Jr., Sacchettini JC (1998) Modification of the NADH of the isoniazid target (InhA) from Mycobacterium tuberculosis. *Science* 279: 98-102

Rudraraju RS, Daher SS, Gallardo-Macias R, Wang X, Neiditch MB, Freundlich JS (2022) Mycobacterium tuberculosis KasA as a drug target: Structure-based inhibitor design. *Front Cell Infect Microbiol* 12: 1008213

Sacco E, Covarrubias AS, O'Hare HM, Carroll P, Eynard N, Jones TA, Parish T, Daffe M, Backbro K, Quemard A (2007) The missing piece of the type II fatty acid synthase system from Mycobacterium tuberculosis. *Proc Natl Acad Sci U S A* 104: 14628-14633

Saridakis E, Vishwakarma R, Lai-Kee-Him J, Martin K, Simon I, Cohen-Gonsaud M, Coste F, Bron P, Margeat E, Boudvillain M (2022) Cryo-EM structure of transcription termination factor Rho from Mycobacterium tuberculosis reveals bicyclomycin resistance mechanism. *Commun Biol* 5: 120

Sassetti CM, Rubin EJ (2003) Genetic requirements for mycobacterial survival during infection. *Proceedings of the National Academy of Sciences of the United States of America* 100: 12989-12994

Schafer AB, Steenhuis M, Jim KK, Neef J, O'Keefe S, Whitehead RC, Swanton E, Wang B, Halbedel S, High S *et al* (2023) Dual Action of Eeyarestatin 24 on Sec-Dependent Protein Secretion and Bacterial DNA. *ACS Infect Dis* 9: 253-269

Schmitt EK, Riwanto M, Sambandamurthy V, Roggo S, Miault C, Zwingelstein C, Krastel P, Noble C, Beer D, Rao SP *et al* (2011) The natural product cyclomarin kills Mycobacterium tuberculosis by targeting the ClpC1 subunit of the caseinolytic protease. *Angew Chem Int Ed Engl* 50: 5889-5891

Schmitz KR, Handy EL, Compton CL, Gupta S, Bishai WR, Sauer RT, Sello JK (2023) Acyldepsipeptide Antibiotics and a Bioactive Fragment Thereof Differentially Perturb Mycobacterium tuberculosis ClpXP1P2 Activity in Vitro. *ACS Chem Biol* 18: 724-733

Schroeder EK, de Souza N, Santos DS, Blanchard JS, Basso LA (2002) Drugs that inhibit mycolic acid biosynthesis in Mycobacterium tuberculosis. *Curr Pharm Biotechnol* 3: 197-225

Segers K, Anne J (2011) Traffic jam at the bacterial sec translocase: targeting the SecA nanomotor by small-molecule inhibitors. *Chem Biol* 18: 685-698

Sengupta S, Ghosh S, Nagaraja V (2008) Moonlighting function of glutamate racemase from Mycobacterium tuberculosis: racemization and DNA gyrase inhibition are two independent activities of the enzyme. *Microbiology (Reading)* 154: 2796-2803

Shao M, McNeil M, Cook GM, Lu X (2020) MmpL3 inhibitors as antituberculosis drugs. *Eur J Med Chem* 200: 112390

Sharma P, Jiang Q, Li SG, Ocke E, Tsotetsi K, Sukheja P, Singh P, Suryavanshi S, Morrison E, Thadkapally S *et al* (2025) Evolution of Small Molecule Inhibitors of Mycobacterium tuberculosis Menaquinone Biosynthesis. *Journal of medicinal chemistry* 68: 5774-5803

Sharma R, Hartman TE, Beites T, Kim JH, Eoh H, Engelhart CA, Zhu L, Wilson DJ, Aldrich CC, Ehrt S *et al* (2023) Metabolically distinct roles of NAD synthetase and NAD kinase define the essentiality of NAD and NADP in Mycobacterium tuberculosis. *mBio* 14: e0034023

Shirude PS, Shandil R, Sadler C, Naik M, Hosagrahara V, Hameed S, Shinde V, Bathula C, Humnabadkar V, Kumar N *et al* (2013) Azaindoles: noncovalent DprE1 inhibitors from scaffold morphing efforts, kill Mycobacterium tuberculosis and are efficacious in vivo. *J Med Chem* 56: 9701-9708

Shirude PS, Shandil RK, Manjunatha MR, Sadler C, Panda M, Panduga V, Reddy J, Saralaya R, Nanduri R, Ambady A *et al* (2014) Lead optimization of 1,4-azaindoles as antimycobacterial agents. *J Med Chem* 57: 5728-5737

Singh A, Jain S, Gupta S, Das T, Tyagi AK (2003) mymA operon of Mycobacterium tuberculosis: its regulation and importance in the cell envelope. *FEMS Microbiol Lett* 227: 53-63

Singh A, Ottavi S, Krieger I, Planck K, Perkowski A, Kaneko T, Davis AM, Suh C, Zhang D, Goullieux L *et al* (2024) Redirecting raltitrexed from cancer cell thymidylate synthase to Mycobacterium tuberculosis phosphopantetheinyl transferase. *Sci Adv* 10: eadj6406

Singh A, Zwerneman LT, Planck K, Kaneko T, Perkowski AJ, Suh CM, Alex A, Bean J, Burns-Huang K, Zhang D *et al* (2025) Thioquinazolinones as Antituberculosis Agents Targeting Phosphopantetheinyl Transferase. *Journal of medicinal chemistry* 68: 14645-14698

Singh S, Goswami N, Tyagi AK, Khare G (2019a) Unraveling the role of the transcriptional regulator VirS in low pH-induced responses of Mycobacterium tuberculosis and identification of VirS inhibitors. *J Biol Chem* 294: 10055-10075

Singh V, Donini S, Pacitto A, Sala C, Hartkoorn RC, Dhar N, Keri G, Ascher DB, Mondesert G, Vocat A *et al* (2017) The Inosine Monophosphate Dehydrogenase, GuaB2, Is a Vulnerable New Bactericidal Drug Target for Tuberculosis. *ACS Infect Dis* 3: 5-17

Singh V, Grzegorzewicz AE, Fienberg S, Muller R, Khonde LP, Sanz O, Alfonso S, Urones B, Drewes G, Bantscheff M *et al* (2022) 1,3-Diarylpyrazolyl-acylsulfonamides Target HadAB/BC Complex in Mycobacterium tuberculosis. *ACS Infect Dis* 8: 2315-2326

Singh V, Pacitto A, Donini S, Ferraris DM, Boros S, Illyes E, Szokol B, Rizzi M, Blundell TL, Ascher DB *et al* (2019b) Synthesis and Structure-Activity relationship of 1-(5-isoquinolinesulfonyl)piperazine analogues as inhibitors of Mycobacterium tuberculosis IMPDH. *Eur J Med Chem* 174: 309-329

Sivaramakrishnan S, de Montellano PR (2013) The DosS-DosT/DosR Mycobacterial Sensor System. *Biosensors (Basel)* 3: 259-282

Slayden RA, Lee RE, Armour JW, Cooper AM, Orme IM, Brennan PJ, Besra GS (1996) Antimycobacterial action of thiolactomycin: an inhibitor of fatty acid and mycolic acid synthesis. *Antimicrob Agents Chemother* 40: 2813-2819

Smith CM, Baker RE, Proulx MK, Mishra BB, Long JE, Park SW, Lee HN, Kiritsy MC, Bellerose MM, Olive AJ *et al* (2022) Host-pathogen genetic interactions underlie tuberculosis susceptibility in genetically diverse mice. *Elife* 11

Smith JM, Warrington NV, Vierling RJ, Kuhn ML, Anderson WF, Koppisch AT, Freel Meyers CL (2014) Targeting DXP synthase in human pathogens: enzyme inhibition and antimicrobial activity of butylacetylphosphonate. *J Antibiot (Tokyo)* 67: 77-83

Soni H, Tyagi S, Mane K, Shelke AM, Kumar P, Kaya F, Alland D, Zimmerman M, Freundlich JS, Nuermberger EL (2025) The KasA inhibitor JSF-3285 improves the sterilizing activity of bedaquiline-pretomanid-containing regimens in a mouse model of tuberculosis. *Antimicrob Agents Chemother* 69: e0013025

Soto R, Perez-Herran E, Rodriguez B, Duma BM, Cacho-Izquierdo M, Mendoza-Losana A, Lelievre J, Aguirre DB, Ballell L, Cox LR *et al* (2018) Identification and characterization of aspartyl-tRNA synthetase inhibitors against Mycobacterium tuberculosis by an integrated whole-cell target-based approach. *Sci Rep* 8: 12664

Stanley RE, Blaha G, Grodzicki RL, Strickler MD, Steitz TA (2010) The structures of the anti-tuberculosis antibiotics viomycin and capreomycin bound to the 70S ribosome. *Nat Struct Mol Biol* 17: 289-293

Stanley SA, Kawate T, Iwase N, Shimizu M, Clatworthy AE, Kazyanskaya E, Sacchettini JC, Ioerger TR, Siddiqi NA, Minami S *et al* (2013) Diarylcoumarins inhibit mycolic acid biosynthesis and kill Mycobacterium tuberculosis by targeting FadD32. *Proc Natl Acad Sci U S A* 110: 11565-11570

Stec J, Onajole OK, Lun S, Guo H, Merenbloom B, Vistoli G, Bishai WR, Kozikowski AP (2016) Indole-2-carboxamide-based MmpL3 Inhibitors Show Exceptional Antitubercular Activity in an Animal Model of Tuberculosis Infection. *J Med Chem* 59: 6232-6247

Steenhuis M, Koningstein GM, Oswald J, Pick T, O'Keefe S, Koch HG, Cavalie A, Whitehead RC, Swanton E, High S *et al* (2021) Eeyarestatin 24 impairs SecYEG-dependent protein trafficking and inhibits growth of clinically relevant pathogens. *Mol Microbiol* 115: 28-40

Stevens CM, Babii SO, Pandya AN, Li W, Li Y, Mehla J, Scott R, Hegde P, Prathipati PK, Acharya A *et al* (2022) Proton transfer activity of the reconstituted Mycobacterium tuberculosis MmpL3 is modulated by substrate mimics and inhibitors. *Proc Natl Acad Sci U S A* 119: e2113963119

Stokes SS, Vemula R, Pucci MJ (2020) Advancement of GyrB Inhibitors for Treatment of Infections Caused by Mycobacterium tuberculosis and Non-tuberculous Mycobacteria. *ACS Infect Dis* 6: 1323-1331

Su H, Lin K, Tiwari D, Healy C, Trujillo C, Liu Y, Ioerger TR, Schnappinger D, Ehrt S (2021) Genetic models of latent tuberculosis in mice reveal differential influence of adaptive immunity. *The Journal of experimental medicine* 218

Sugie Y, Inagaki S, Kato Y, Nishida H, Pang CH, Saito T, Sakemi S, Dib-Hajj F, Mueller JP, Sutcliffe J *et al* (2002) CJ-21,058, a new SecA inhibitor isolated from a fungus. *J Antibiot (Tokyo)* 55: 25-29

Sukheja P, Kumar P, Mittal N, Li SG, Singleton E, Russo R, Perryman AL, Shrestha R, Awasthi D, Husain S *et al* (2017) A Novel Small-Molecule Inhibitor of the Mycobacterium tuberculosis Demethylmenaquinone Methyltransferase MenG Is Bactericidal to Both Growing and Nutritionally Deprived Persister Cells. *mBio* 8

Sun S, Ginn J, Kochanczyk T, Arango N, Jiang X, Huggins DJ, Bean J, Michino M, Baxt L, Liverton N *et al* (2024) Indazole to 2-Cyanoindole Scaffold Progression for Mycobacterial Lipoamide Dehydrogenase Inhibitors Achieves Extended Target Residence Time and Improved Antibacterial Activity. *Angew Chem Int Ed Engl* 63: e202407276

Suresh M, Naicker K, Solanki J, Ezirim SA, Turcio R, Tochukwu IG, Lakhdari K, Attah EI (2023) Ligand-based pharmacophore modelling, virtual screening and docking studies to identify potential compounds against FtsZ of Mycobacterium tuberculosis. *Indian J Tuberc* 70: 430-444

Swanson S, Ioerger TR, Rigel NW, Miller BK, Braunstein M, Sacchettini JC (2015) Structural Similarities and Differences between Two Functionally Distinct SecA Proteins, Mycobacterium tuberculosis SecA1 and SecA2. *J Bacteriol* 198: 720-730

Tam PC, Maillard AP, Chan KK, Duong F (2005) Investigating the SecY plug movement at the SecYEG translocation channel. *EMBO J* 24: 3380-3388

Tantry SJ, Markad SD, Shinde V, Bhat J, Balakrishnan G, Gupta AK, Ambady A, Raichurkar A, Kedari C, Sharma S *et al* (2017) Discovery of Imidazo[1,2-a]pyridine Ethers and Squaramides as Selective and Potent Inhibitors of Mycobacterial Adenosine Triphosphate (ATP) Synthesis. *Journal of medicinal chemistry* 60: 1379-1399

Telenti A, Philipp WJ, Sreevatsan S, Bernasconi C, Stockbauer KE, Wieles B, Musser JM, Jacobs WR, Jr. (1997) The emb operon, a gene cluster of Mycobacterium tuberculosis involved in resistance to ethambutol. *Nature medicine* 3: 567-570

Temrikar ZH, Kodidela S, Kumar S, Liu J, Robertson GT, Lee RE, Hickey AJ, Gonzalez-Juarrero M, Meibohm B (2023) Characterization of spectinamide 1599 efficacy against different mycobacterial phenotypes. *Tuberculosis (Edinb)* 140: 102342

Thongdee P, Hanwarinroj C, Pakamwong B, Kamsri P, Punkvang A, Leanpolchareanchai J, Ketrat S, Saparpakorn P, Hannongbua S, Ariyachaokun K *et al* (2022) Virtual Screening Identifies Novel and Potent Inhibitors of Mycobacterium tuberculosis PknB with Antibacterial Activity. *J Chem Inf Model* 62: 6508-6518

Tiwari D, Park SW, Essawy MM, Dawadi S, Mason A, Nandakumar M, Zimmerman M, Mina M, Ho HP, Engelhart CA *et al* (2018) Targeting protein biotinylation enhances tuberculosis chemotherapy. *Sci Transl Med* 10

Trivedi OA, Arora P, Sridharan V, Tickoo R, Mohanty D, Gokhale RS (2004) Enzymic activation and transfer of fatty acids as acyl-adenylates in mycobacteria. *Nature* 428: 441-445

Tufariello JM, Chapman JR, Kerantzas CA, Wong KW, Vilcheze C, Jones CM, Cole LE, Tinaztepe E, Thompson V, Fenyo D *et al* (2016) Separable roles for Mycobacterium tuberculosis ESX-3 effectors in iron acquisition and virulence. *Proceedings of the National Academy of Sciences of the United States of America* 113: E348-357

Veenendaal AK, van der Does C, Driessen AJ (2004) The protein-conducting channel SecYEG. *Biochim Biophys Acta* 1694: 81-95

Velappan AB, Datta D, Ma R, Rana S, Ghosh KS, Hari N, Franzblau SG, Debnath J (2020) 2-Aryl benzazole derived new class of anti-tubercular compounds: Endowed to eradicate mycobacterium tuberculosis in replicating and non-replicating forms. *Bioorg Chem* 103: 104170

Venugopal A, Bryk R, Shi S, Rhee K, Rath P, Schnappinger D, Ehrt S, Nathan C (2011) Virulence of Mycobacterium tuberculosis depends on lipoamide dehydrogenase, a member of three multienzyme complexes. *Cell host & microbe* 9: 21-31

Vieira TF, Martins FG, Moreira JP, Barbosa T, Sousa SF (2021) In Silico Identification of Possible Inhibitors for Protein Kinase B (PknB) of Mycobacterium tuberculosis. *Molecules* 26

Volynets GP, Usenko MO, Gudzera OI, Starosyla SA, Balanda AO, Syniugin AR, Gorbatiuk OB, Prykhod'ko AO, Bdzhola VG, Yarmoluk SM *et al* (2022) Identification of dual-targeted Mycobacterium tuberculosis aminoacyl-tRNA synthetase inhibitors using machine learning. *Future Med Chem* 14: 1223-1237

Wallis RS, Dawson R, Friedrich SO, Venter A, Paige D, Zhu T, Silvia A, Gobey J, Ellery C, Zhang Y *et al* (2014) Mycobactericidal activity of sutezolid (PNU-100480) in sputum (EBA) and blood (WBA) of patients with pulmonary tuberculosis. *PLoS One* 9: e94462

Wang S, Wang K, Song K, Lai ZW, Li P, Li D, Sun Y, Mei Y, Xu C, Liao M (2024) Structures of the Mycobacterium tuberculosis efflux pump EfpA reveal the mechanisms of transport and inhibition. *Nature communications* 15: 7710

Wang T, Bemis G, Hanzelka B, Zuccola H, Wynn M, Moody CS, Green J, Locher C, Liu A, Gao H *et al* (2017a) Mtb PKNA/PKNB Dual Inhibition Provides Selectivity Advantages for Inhibitor Design To Minimize Host Kinase Interactions. *ACS Med Chem Lett* 8: 1224-1229

Wang X, Ahn YM, Lentscher AG, Lister JS, Brothers RC, Kneen MM, Gerratana B, Boshoff HI, Dowd CS (2017b) Design, synthesis, and evaluation of substituted nicotinamide adenine dinucleotide (NAD(+)) synthetase inhibitors as potential antitubercular agents. *Bioorg Med Chem Lett* 27: 4426-4430

Warapande V, Meng F, Bozan A, Graff DE, Fromer JC, Mughal K, Mohideen FK, Shivangi, Paruchuri S, Johnston ML *et al* (2025) Identification of Antituberculars with Favorable Potency and Pharmacokinetics through Structure-Based and Ligand-Based Modeling. *bioRxiv*

Wellington S, Nag PP, Michalska K, Johnston SE, Jedrzejczak RP, Kaushik VK, Clatworthy AE, Siddiqi N, McCarren P, Bajrami B *et al* (2017) A small-molecule allosteric inhibitor of Mycobacterium tuberculosis tryptophan synthase. *Nat Chem Biol* 13: 943-950

Wetzel J, Dallow J, Davis E, Pearson WH, Daems S, Govaerts M, Hereijgers J, Sprangers J, Truebody B, Maes V *et al* (2026) Menaquinone depletion resensitises bedaquiline-resistant tuberculosis. *bioRxiv*: 2026.2002.2010.702511

Wilburn KM, Montague CR, Qin B, Woods AK, Love MS, McNamara CW, Schultz PG, Southard TL, Huang L, Petrassi HM *et al* (2022) Pharmacological and genetic activation of cAMP synthesis disrupts cholesterol utilization in Mycobacterium tuberculosis. *PLoS pathogens* 18: e1009862

Williams JT, Giletto M, Haiderer ER, Aleiwi B, Krieger-Burke T, Ellsworth E, Abramovitch RB (2024) The Mycobacterium tuberculosis MmpL3 inhibitor MSU-43085 is active in a mouse model of infection. *Microbiol Spectr* 12: e0367723

Williams JT, Haiderer ER, Coulson GB, Conner KN, Ellsworth E, Chen C, Alvarez-Cabrera N, Li W, Jackson M, Dick T *et al* (2019) Identification of New MmpL3 Inhibitors by Untargeted and Targeted Mutant Screens Defines MmpL3 Domains with Differential Resistance. *Antimicrob Agents Chemother* 63

Wilson C, Ray P, Zuccotto F, Hernandez J, Aggarwal A, Mackenzie C, Caldwell N, Taylor M, Huggett M, Mathieson M *et al* (2022) Optimization of TAM16, a Benzofuran That Inhibits the Thioesterase Activity of Pks13; Evaluation toward a Preclinical Candidate for a Novel Antituberculosis Clinical Target. *J Med Chem* 65: 409-423

Wilson R, Kumar P, Parashar V, Vilcheze C, Veyron-Churlet R, Freundlich JS, Barnes SW, Walker JR, Szymonifka MJ, Marchiano E *et al* (2013) Antituberculosis thiophenes define a requirement for Pks13 in mycolic acid biosynthesis. *Nat Chem Biol* 9: 499-506

Wisedchaisri G, Wu M, Sherman DR, Hol WG (2008) Crystal structures of the response regulator DosR from Mycobacterium tuberculosis suggest a helix rearrangement mechanism for phosphorylation activation. *J Mol Biol* 378: 227-242

Wlodarchak N, Teachout N, Beczkiewicz J, Procknow R, Schaenzer AJ, Satyshur K, Pavelka M, Zuercher W, Drewry D, Sauer JD *et al* (2018) In Silico Screen and Structural Analysis Identifies Bacterial Kinase Inhibitors which Act with beta-Lactams To Inhibit Mycobacterial Growth. *Mol Pharm* 15: 5410-5426

Wolucka BA (2008) Biosynthesis of D-arabinose in mycobacteria - a novel bacterial pathway with implications for antimycobacterial therapy. *FEBS J* 275: 2691-2711

Wolucka BA, McNeil MR, de Hoffmann E, Chojnacki T, Brennan PJ (1994) Recognition of the lipid intermediate for arabinogalactan/arabinomannan biosynthesis and its relation to the mode of action of ethambutol on mycobacteria. *The Journal of biological chemistry* 269: 23328-23335

Woong Park S, Klotzsche M, Wilson DJ, Boshoff HI, Eoh H, Manjunatha U, Blumenthal A, Rhee K, Barry CE, 3rd, Aldrich CC *et al* (2011) Evaluating the sensitivity of Mycobacterium tuberculosis to biotin deprivation using regulated gene expression. *PLoS pathogens* 7: e1002264

Wu H, Huang MY, Xue ZJ, Zhong SY, Shi JQ, Chen NP, Qian CD (2025) Targeting type II NADH dehydrogenase in tuberculosis treatment: A review. *Int J Biol Macromol* 310: 143541

Xu J, Wang JX, Zhou JM, Xu CL, Huang B, Xing Y, Wang B, Luo R, Wang YC, You XF *et al* (2017a) A novel protein kinase inhibitor IMB-YH-8 with anti-tuberculosis activity. *Sci Rep* 7: 5093

Xu Y, Ehrt S, Schnappinger D, Beites T (2023) Synthetic lethality of Mycobacterium tuberculosis NADH dehydrogenases is due to impaired NADH oxidation. *mBio* 14: e0104523

Xu Z, Meshcheryakov VA, Poce G, Chng SS (2017b) MmpL3 is the flippase for mycolic acids in mycobacteria. *Proc Natl Acad Sci U S A* 114: 7993-7998

Zaunbrecher MA, Sikes RD, Jr., Metchock B, Shinnick TM, Posey JE (2009) Overexpression of the chromosomally encoded aminoglycoside acetyltransferase eis confers kanamycin resistance in Mycobacterium tuberculosis. *Proc Natl Acad Sci U S A* 106: 20004-20009

Zhan W, Hsu HC, Morgan T, Ouellette T, Burns-Huang K, Hara R, Wright AG, Imaeda T, Okamoto R, Sato K *et al* (2019) Selective Phenylimidazole-Based Inhibitors of the Mycobacterium tuberculosis Proteasome. *Journal of medicinal chemistry* 62: 9246-9253

Zhang H, Chen Y, Zhang Y, Qiao L, Chi X, Han Y, Lin Y, Si S, Jiang J (2023a) Identification of anti-Mycobacterium tuberculosis agents targeting the interaction of bacterial division proteins FtsZ and SepFe. *Acta Pharm Sin B* 13: 2056-2070

Zhang H, Hsu HC, Kahne SC, Hara R, Zhan W, Jiang X, Burns-Huang K, Ouellette T, Imaeda T, Okamoto R *et al* (2021) Macrocyclic Peptides that Selectively Inhibit the Mycobacterium tuberculosis Proteasome. *Journal of medicinal chemistry* 64: 6262-6272

Zhang J, Lair C, Roubert C, Amaning K, Barrio MB, Benedetti Y, Cui Z, Xing Z, Li X, Franzblau SG *et al* (2023b) Discovery of natural-product-derived sequanamycins as potent oral anti-tuberculosis agents. *Cell* 186: 1013-1025 e1024

Zhang L, Zhao Y, Gao Y, Wu L, Gao R, Zhang Q, Wang Y, Wu C, Wu F, Gurcha SS *et al* (2020) Structures of cell wall arabinosyltransferases with the anti-tuberculosis drug ethambutol. *Science (New York, NY)* 368: 1211-1219

Zhang M, Li K, Zhang C, Gai J, Yu D (2009) Identification and characterization of class 1 DXS gene encoding 1-deoxy-D-xylulose-5-phosphate synthase, the first committed enzyme of the MEP pathway from soybean. *Mol Biol Rep* 36: 879-887

Zhao H, Gao Y, Li W, Sheng L, Cui K, Wang B, Fu L, Gao M, Lin Z, Zou X *et al* (2022) Design, Synthesis, and Biological Evaluation of Pyrrole-2-carboxamide Derivatives as Mycobacterial Membrane Protein Large 3 Inhibitors for Treating Drug-Resistant Tuberculosis. *J Med Chem* 65: 10534-10553

Zheng H, Williams JT, Aleiwi B, Ellsworth E, Abramovitch RB (2020) Inhibiting Mycobacterium tuberculosis DosRST Signaling by Targeting Response Regulator DNA Binding and Sensor Kinase Heme. *ACS Chem Biol* 15: 52-62

Zheng J, Rubin EJ, Bifani P, Mathys V, Lim V, Au M, Jang J, Nam J, Dick T, Walker JR *et al* (2013) para-Aminosalicylic acid is a prodrug targeting dihydrofolate reductase in Mycobacterium tuberculosis. *J Biol Chem* 288: 23447-23456

Zhu D, Johannsen S, Masini T, Simonin C, Haupenthal J, Illarionov B, Andreas A, Awale M, Gierse RM, van der Laan T *et al* (2022) Discovery of novel drug-like antitubercular hits targeting the MEP pathway enzyme DXPS by strategic application of ligand-based virtual screening. *Chem Sci* 13: 10686-10698
